# Searching for drug leads targeted to the hydrophobic cleft of dengue virus capsid protein

**DOI:** 10.1080/14756366.2021.2004591

**Published:** 2021-12-11

**Authors:** Liliane O. Ortlieb, Ícaro P. Caruso, Nathane C. Mebus-Antunes, Andrea T. Da Poian, Elaine da C. Petronilho, José Daniel Figueroa-Villar, Claudia J. Nascimento, Fabio C. L. Almeida

**Affiliations:** a Department of Chemistry, Military Institute of Engineering (IME), Rio de Janeiro, Brazil; b Institute of Medical Biochemistry Leopoldo de Meis (IBqM) and National Center for Structural Biology and Bioimaging (CENABIO), Federal University of Rio de Janeiro (UFRJ), Rio de Janeiro, Brazil; c Multiuser Center for Biomolecular Innovation (CMIB) and Department of Physics, Institute of Biosciences, Letters and Exact Sciences (IBILCE), São Paulo State University (UNESP), São José do Rio Preto, Brazil; d Institute of Medical Biochemistry Leopoldo de Meis (IBqM), Federal University of Rio de Janeiro (UFRJ), Rio de Janeiro, Brazil; e Department of Natural Sciences, Institute of Biosciences, Federal University of the State of Rio de Janeiro (UNIRIO), Rio de Janeiro, Brazil

**Keywords:** Dengue virus, DENVC, NMR, drug-ligand interaction, fluorescence

## Abstract

We synthesised and screened 18 aromatic derivatives of guanylhydrazones and oximes aromatic for their capacity to bind to dengue virus capsid protein (DENVC). The intended therapeutic target was the hydrophobic cleft of DENVC, which is a region responsible for its anchoring in lipid droplets in the infected cells. The inhibition of this process completely suppresses virus infectivity. Using NMR, we describe five compounds able to bind to the α1-α2 interface in the hydrophobic cleft. Saturation transfer difference experiments showed that the aromatic protons of the ligands are important for the interaction with DENVC. Fluorescence binding isotherms indicated that the selected compounds bind at micromolar affinities, possibly leading to binding-induced conformational changes. NMR-derived docking calculations of ligands showed that they position similarly in the hydrophobic cleft. Cytotoxicity experiments and calculations of *in silico* drug properties suggest that these compounds may be promising candidates in the search for antivirals targeting DENVC.

## 1. Introduction

The dengue virus (DENV) is a mosquito-borne virus that belongs to the *Flavivirus* genus, together with other important human pathogens, such as Zika, yellow fever, West Nile, and Japanese encephalitis viruses. The incidence of DENV is increasing over the years by the mosquito vector spreading, which can be associated with urbanisation, global warming, population growth, and an increasing number of international travels, together with a decrease in effective means for mosquito control^1^. The disease caused by DENV may vary from a mild fever to life-threatening severe diseases, known as dengue haemorrhagic fever (DHF) and dengue shock syndrome (DSS), which are characterised by an increase in vascular endothelial permeability that leads to plasma leakage, and may evolve to a fatal hypovolemic shock^2^.

DENV capsid protein (DENVC) forms a symmetric dimer in solution, presenting 8 intertwined α-helices (4 per subunit)^3^. DENVC has an essential role in the viral assembly. It is involved with the packaging of the viral genome forming the nucleocapsid (NC) core^4^. The protein is dominated by quaternary contacts involving two pairs of antiparallel helices (α2-α2' and α4-α4') that form most of the dimer interface. The α4-α4' exposed surface has the highest density of positive charges and it is the putative RNA binding site. The α2-α2' is nonpolar and along with α1 and α1' form a concave-shaped hydrophobic cleft, that interacts with the viral membrane. The dynamics, size, and orientation of α1 and α1' regulate the exposure of the hydrophobic surface^5^^,^^6^. Among flaviviruses, α2-α2' is the most conserved region of protein C, helping in the formation of a conserved hydrophobic surface (π-stacked Phe53/Phe53’, Phe47, Leu54, and Leu57) and a conserved aromatic backbone (π-stacked Phe56/Phe84’ and Phe56’/Phe84)^6^. Samsa and cols^7^ showed that DENVC associates with lipid droplets (LD) during viral replication, being this event essential to the virus assembly and infectivity. The interaction to LD involves the hydrophobic cleft of the DENVC and the conserved segment 14–23 of the intrinsically disordered N-terminal^8^^,^^9^. Mutations in hydrophobic amino acid residues at the hydrophobic cleft completely abolished the virus infectivity, which makes it an important unexplored therapeutical target^7^.

Some antiviral compounds were identified as being able of inhibiting *in vitro* and *in vivo* DENV replication. Different targets were used in those studies, such as viral envelope (E) protein, inhibiting viral-induced membrane fusion^10^; NS2B-NS3, inhibiting viral protease activity^11^; NS4B, possibly inhibiting its interaction with the viral NS3 helicase domain^12^; NS5 methyltransferase activity^13^ or RNA polymerase activity^14^^,^^15^, inhibiting viral RNA synthesis; and virus-specific RNA translation^16^. Regarding DENVC, the small compound ST-148 was identified as a ligand of C protein^17^^,^^18^, with a proposed mechanism of capsid assembly inhibition through its binding to the protein hydrophobic cleft, leading to the formation of a kissing tetramer (dimer of dimers)^19^. Despite some of these ligands having shown valid antiviral activity against DENV replication, until now, to our knowledge, there are no interaction studies by NMR and no approved antiviral drug for the treatment of DENV infections.

**Figure 1.**
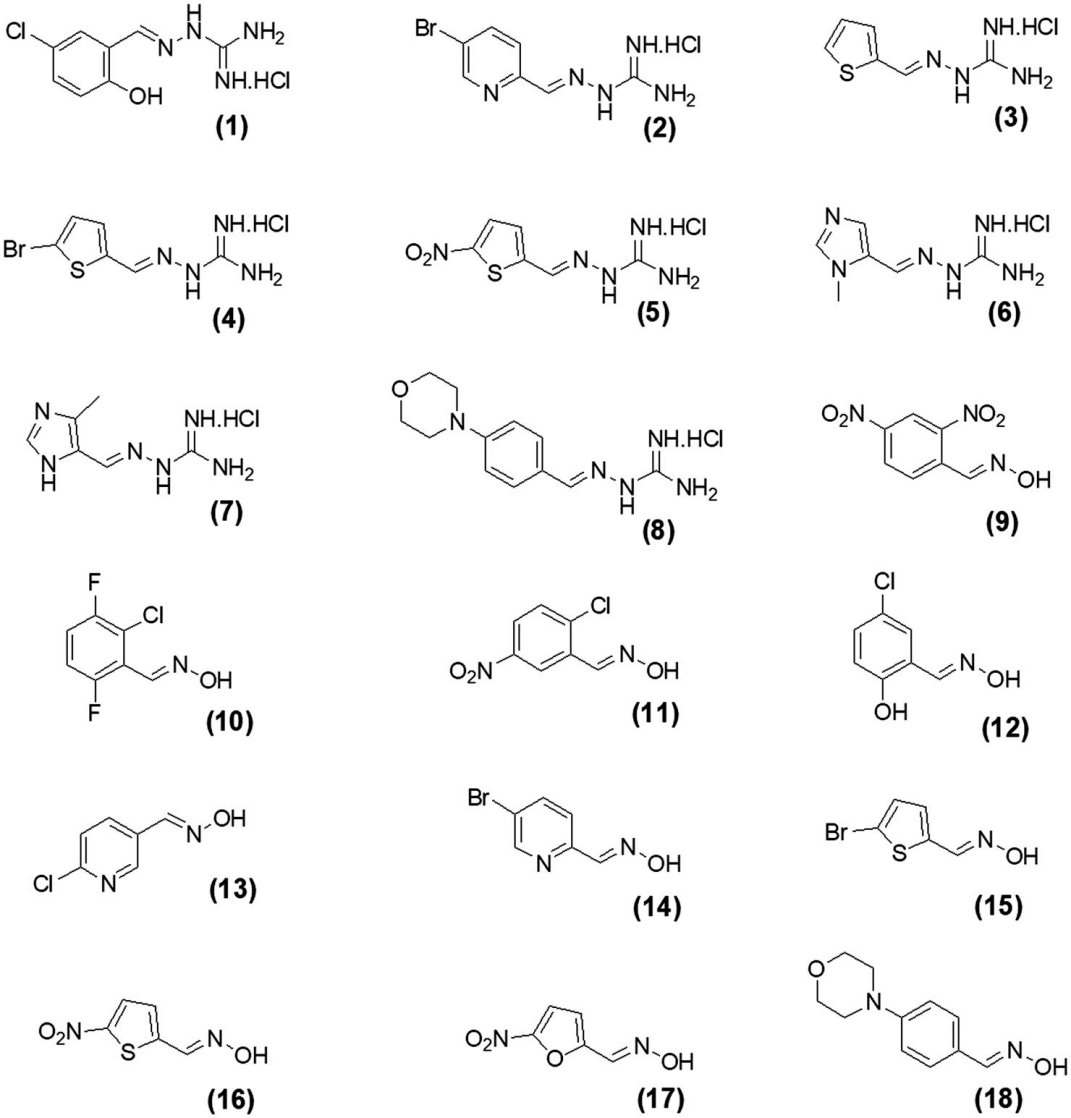
Organic compounds used in this work: (**1**) 5-chloro-salicylaldehyde-guanylhydrazone, (**2**) 5-bromo-pyridine-2-guanylhydrazone, (**3**) thiophene-2-guanylhydrazone, (**4**) 5-bromo-thiophene-2-guanylhydrazone, (**5**) 5-nitro-thiophene-2-guanylhydrazone, (**6**) 1-methyl-imidazole-5-guanylhydrazone, (**7**) 4-methyl-imidazole-5-guanylhydrazone, (**8**) 4–(4-morpholinyl)benzyl-guanylhydrazone, (**9**) 2,4-dinitro-benzyloxime, (**10**) 2-chloro-3,6-difluoro-benzyloxime, (**11**) 2-chloro-5-nitro-benzyloxime, (**12**) 2-hydroxy-5-chloro-benzyloxime, (**13**) 6-chloro-pyridine-3-oxime, (**14**) 5-bromo-pyridine-2-oxime, (**15**) 5-bromo-thiophene-2-oxime, (**16**) 5-nitro-thiophene-2-oxime, (**17**) 5-nitro-furfural-2-oxime, and (**18**) 4–(4-morphonylyl)benzyloxime.

In this work, we screened by NMR 18 aromatic compounds (Figure 1, derivatives of guanylhydrazones and oximes) for their capacity to bind DENVC. The intended target was the hydrophobic cleft. We selected 5 leads that bind to the hydrophobic cleft at the interface between α1/α1’ and α2/α2’. We used ligand-based NMR methods (saturation transfer difference, STD, and transverse relaxation, *T*_2_) and protein-based ^15 ^N-HSQC for screenings. *In silico* calculations were also performed to rationalise the results. Structural models of the protein-ligand complexes generated by NMR-derived molecular docking showed that the selected compounds could bind to the two symmetric pockets in the hydrophobic cleft. Pharmacokinetics analyses showed the tested compounds are promising drugs, with low cytotoxic effects on two different cell lines (Huh7 and A549). We successfully addressed the hydrophobic cleft of DENVC as a structural target for the development of potential antiviral compounds.

## 2. Materials and methods

### 2.1. Compounds selection

The selection of the compounds used in this work (Figure 1) was based on the fact that aromatic and amphipathic compounds are expected to interact with the nonpolar exposed residues and the aromatic backbone in DENVC hydrophobic cleft. In addition, it has been taken into account fact that derivatives of guanylhydrazones and oximes are widely studied due to their broad biological activity, as antitumor^20^^,^^21^, antibacterial^22^^,^^23^, antifungal^24^^,^^25^, antiviral^26^^,^^27^, antiprotozoal^28^^,^^29^, and anti-inflammatory^30^^,^^31^ drugs.

### 2.2. Synthesis and characterisation of the organic compounds

All reagents were purchased from Sigma-Aldrich (Brazil) and the solvents from VETEC (Brazil), used without further purification. Reactions were monitored by TLC using DC Alufolien Kieselgel 60 F254 (Merck, Darmstadt, Germany). Melting points (MP) were determined on a Melt-Temp II with a previously calibrated thermometer. Infra-red spectra (IR) were obtained on a Shimadzu Prestige 21. For the characterisation of compounds, all NMR experiments (^1^H NMR, ^13 ^C NMR, ATP, gHSQC, gHMBC) were performed at 298 K on a 14.1 T Premium COMPACT™ (600 MHz for proton, software VNMRJ version 3.2) spectrometer using a 5 mm NMR probe and dimethylsulfoxide-*d_6_* (DMSO-*d_6_*) as solvent and reference. The data of yield, melting point, IR and NMR spectra (Figures S1–S54), and spectral assignment are available in the Supplementary Information.

#### 2.2.1. General procedure for the synthesis of guanilhydrazones (1–8):

Aminoguanidine hydrochloride (1.2 mmols) dissolved in 20 ml of 95% ethanol, the corresponding aldehyde (1 mmol) and 2 drops of HCl (0.6 M) were placed in a round bottom flask. The solution was kept under reflux and stirring. The solid obtained after eliminating the solvent under vacuum was solubilised in distilled water and extracted with dichloromethane (5 × 20 ml). The product was recrystallized from ethanol^32^.

#### 2.2.2. General procedure for the synthesis of oximes (9–18):

Hydroxylamine hydrochloride (4 mmols) dissolved in a mixture of 10 ml of ethanol and 3 ml of water was placed in a round bottom flask. The corresponding aldehyde (2 mmols) was added to the solution, which was kept under stirring. The solid obtained after eliminating the solvent under vacuum was washed with distilled cool water. The oximes were recrystallized from ethanol or methanol^32^. The compounds **1**, **3**, **12**, **15**, **16**, and **17** were synthesised as previously reported^33–38^. The synthesis routes were the same as published, optimising the quantities of the reagents and time of reaction.

### 2.3. DENEC expression and purification

DENVC from serotype 2, strain New Guinea, was expressed in *Escherichia coli* BL21(DE3)plysS cells with pET3a (Novagen) expression plasmid in M9 minimal medium, as previously described^3^^,^^39^. For expression of the ^15 ^N-labelled protein, the minimal medium containing ^15^NH_4_Cl 1 g.L^−1^ (Sigma Aldrich) was used. The DENVC was purified using a heparin column (GE Healthcare LifeSciences) at 5 ml/min flow rate and the bound protein was eluted with an increasing NaCl concentration gradient (0.5–2.0 M). The fractions containing the DENVC were confirmed by 15% SDS-PAGE. The DENVC was dialysed against phosphate-buffered saline (PBS) with a Centricon instrument (Millipore) and stored at −20 °C.

### 2.4. Ligand-protein interaction studies by NMR

Stock solutions of compounds **1**–**8** were prepared in H_2_O. Due to the low solubility in water, stock solutions of compounds **9**–**18** were prepared in DMSO-*d_6_*. For the NMR experiments, samples were prepared using the necessary amount of stock solution in 55 mM PBS, 200 mM NaCl, 2 mM EDTA, 10% (v/v) D_2_O (Cambridge Isotope Laboratories) as buffer solution at pH 6.0.

All experiments were acquired using the samples in the presence and absence of DENVC, with the final concentration of 10 μM for the protein and 1 mM for ligands (100-fold excess). For compounds **9**–**18**, the final concentration of DMSO-*d_6_* was 5% (v/v). All experiments were performed at 308 K, using a 5 mm NMR probe. Spectral data were processed with Topspin 2.1 (Biospin; Bruker).

Initially, the saturation-transfer difference (STD) experiments were performed in pools of 4 or 5 compounds, using STDdiff pulse sequence, 8 scans, 4 dummy scans, off-resonance irradiation at −6000 Hz, and on-resonance irradiation at 124.7, 746, and 2688 Hz, on a Bruker Avance DRX 400 MHz (Bruker Biospin, Germany). For those compounds that showed interaction, new STD spectra were acquired for each compound separately under the same previously described conditions on a Bruker Ascend 500 MHz spectrometer (Bruker Biospin, Germany).

The ^1^H NMR chemical shift variations were recorded using zgesgp pulse sequence (excitation sculpting for water suppression)^40^ with 32 scans and 8 dummy scans. DOSY experiments were acquired using bipolar gradients (stebpgp pulse sequence) with 128 scans, 4 dummy scans, 20 ms of diffusion time (big delta), and 10 ms of gradient pulse (little delta). Both experiments were performed on a Bruker Avance DRX 400 MHz (Bruker Biospin, Germany).

The diffusion coefficients calculations were calculated from the adjustment of curves using the following fitting equation, where *A* and *A_0_* are the areas of the NMR signal in the presence and absence of external gradient pulses, respectively; *D* is the diffusion coefficient; *γ* is the gyromagnetic ratio of the observed nucleus; *g* is the gradient strength; Δ is the diffusion time; *δ* is the length of the gradient.

(1) $$A=A_{0}e\mathrm{xp}[-D\cdot \gamma^{2}\cdot g^{2}\cdot \delta^{2}\cdot (\Delta -\frac{\delta }{3})]$$

Relaxation times were acquired on a Bruker Ascend 500 MHz spectrometer (Bruker Biospin, Germany) and performed in triplicate with a recycle delay of 10 s. *T*_1_ relaxation time was measured using the inversion recovery pulse sequence and *T*_2_ relaxation time by Carr-Purcell-Meiboom-Gill (CPMG) pulse sequence.

In addition, protein-ligand studies for determining the binding sites of the protein were performed only for those ligands that showed interaction by STD. HSQC ^1^H-^15^N spectra were acquired using the uniformly labelled ^15 ^N DENVC. The experiments were performed in the presence and absence of the ligands. For the first titration point, a 200 µM DENVC solution was used. For the next four titration points, it was used a molar excess of the ligands: 1, 3, 6, and 9 relatives to the protein. Samples were prepared in PBS buffer (55 mM NaH_2_PO_4_/Na_2_HPO_4_, 200 mM NaCl, 5 mM EDTA, pH 6.0) with 10% (v/v) D_2_O (Cambridge Isotope Laboratories), and 5% (v/v) DMSO-*d_6_* (Cambridge Isotope Laboratories). Spectra were acquired on a Bruker Avance III 18.6 Tesla (800 MHz for hydrogen) spectrometer with a 5 mm probe at 308 K using the hsqcetf3gpsi pulse sequence, water suppression by water flip back and gradients^41–44^, 8 scans, 16 dummy scans, 1024 × 256 dot spectral window. The experiments were processed using the CcpNmr Analysis software^45^ and were assigned according to NMR data (PDB ID code 1R6R)^46^ obtained from the Biological Magnetic Resonance Data Bank (BMRB)^47^.

### 2.5. Fluorescence analysis

**Figure 2.**
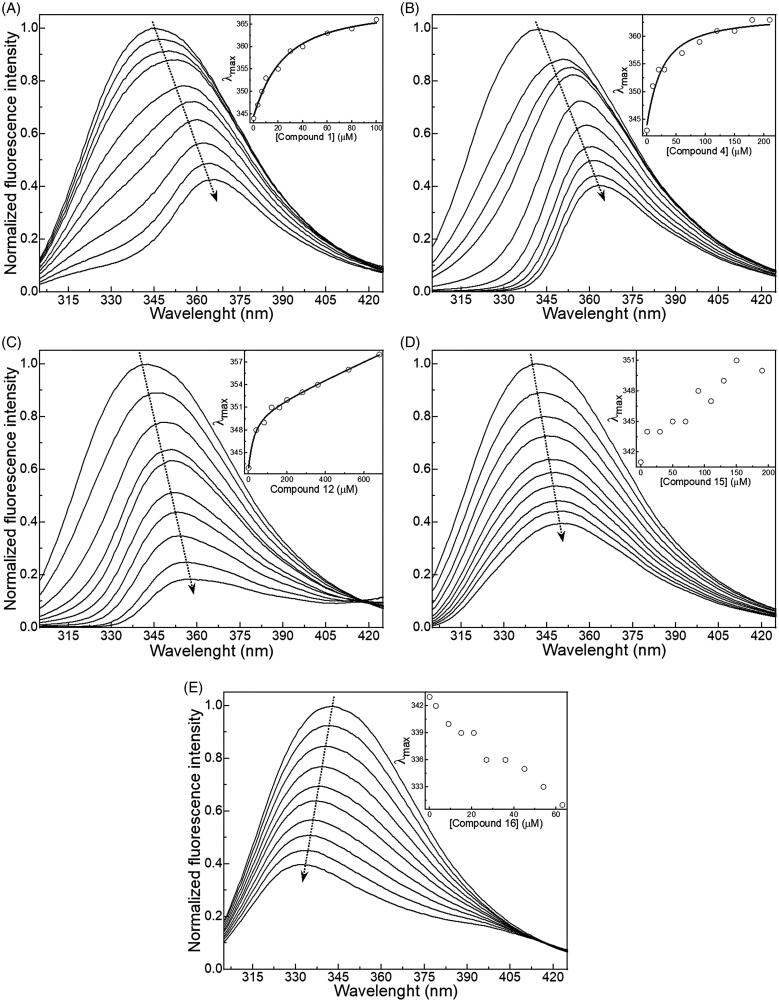
Effect of intrinsic fluorescence on the relative intensity of the signal of DENVC (10 μM) obtained by titration of compounds with excitation at 280 nm and scanning emission between 305 and 425 nm in the absence and presence of compound: (A) **1**, (B) **4**, (C) **12**, (D) **15**, and (E) **16**. The insets show the change in the maximum emission wavelength ($\lambda_{\max},$ open circle) as a function of the selected compound concentration. The black line in the insets denotes the better fitting to the experimental data.

Fluorescence measurements were taken in a Varian Cary Eclipse Fluorescence Spectrophotometer at 308 K. One scan was performed on a 10 µM DENVC sample in PBS buffer using an excitation wavelength of 280 nm and emission detected from 300 to 420 nm with slits set to 5 nm (excitation) and 10 nm (emission). Stock solutions of compounds **1** and **4** were prepared in H_2_O and for compounds **12**, **15,** and **16**, due to their low solubility in water, in DMSO-*d_6_*. The protein sample was titrated by adding aliquots of the ligand stock solution in different concentrations, according to each protocol, as described in Figure 2.

A total of 10 titration points was acquired for each inhibitor, compound **1**: apo, 3.33, 6.67, 10, 20, 30, 40, 60, 80, and 100 µM; compound **4**: apo, 10, 20, 30, 60, 90, 120, 150, 180, and 210 µM; compound **12**: apo, 40, 80, 120, 160, 200, 280, 360, 520, and 680 µM; compound **15**: apo, 10, 30, 50, 70, 90, 110, 130, 150, and 190 µM; and compound **16**: apo, 3, 9, 15, 21, 27, 36, 45, 54, and 63 µM.

Before each measurement, the samples were placed to reach thermal equilibrium for 10 min. Each experiment was performed in triplicate and the solvent (blank) with each compound was subtracted from the mean of three means of three replicate samples. The values of maximum emission wavelength ($\lambda_{\max}$) were used to build the binding isotherms for the DENVC/selected compound interactions. The program Origin 2021 was employed to fit the following equation to the experimental data^48^:

(2) $$\lambda_{\max}=\lambda_{\max}^{0}+(\frac{\lambda_{\max}^{\mathrm{sat}}-\lambda_{\max}^{0}}{2\cdot [P_{T}]})[(K_{d}+[L_{T}]+[P_{T}])-\sqrt{{\left(K_{d}+[L_{T}]+[P_{T}]\right)}^{2}-4\cdot [L_{T}]\cdot [P_{T}]}]$$

where $\lambda_{\max}^{0}$ and $\lambda_{\max}^{\mathrm{sat}}$ are the initial and saturation maximum emission wavelength, respectively, $K_{d}$ is the dissociation constant, $\left[L_{T}\right]$ is the total concentration of the selected compound, and $\left[P_{T}\right]$ is the total concentration of the DENVC. For compound **12**, it was added a linear contribution to Equation (2) to perform the fitting.

### 2.6. Pharmacokinetic and toxicological properties

*In silico* pharmacokinetic and toxicological properties (molecular weight (g/mol), relative polar surface area (PSA) (Å), lipophilicity (c-LogP), water solubility (c-LogS), donor sites (nOHNH), hydrogen bonding acceptors (nOH), number of rotatable bonds, and toxicity), using DataWarrior software^49^, were calculated for the five compounds that showed interaction by STD-NMR experiment.

### 2.7. Molecular docking

The DENVC structure used for the computational simulations was downloaded from Protein Data Bank (PDB) under access code 1R6R^46^. The molecular structures of the compounds were obtained by structural optimisation calculations from the semi-empirical PM6 method using the Gaussian 09 program^50^. DENVC and compounds were prepared using AutoDockTools program^51^ for molecular docking simulations, merging non-polar hydrogen atoms, and adding atom types. The rigid root of the compounds was generated automatically, setting all possible rotatable bonds defined as active by torsions. The molecular docking calculations were performed in triplicate by using AutoDock Vina^52^, applying a total of 8 exhaustiveness. The coordinates of the centre of the conformational search box were defined to enrol the whole hydrophobic cleft (α1/α1’ and α2/α2’) in DENVC since ^1^H-^15^N HSQC experiments indicate that all compounds bind to this protein region. The box dimensions were 40 × 30 × 20 Å on the three coordinate axes. Following docking calculations, the lowest energy structural models of DENVC/compound complexes were analysed by PLIP webserver^53^ for characterising the protein/ligand non-covalent interactions, such as hydrophobic contacts, hydrogen bond, π-cation interaction, and halogen bonds. Structural conformation of the constructed models was displayed using PyMOL^54^.

### 2.8. Cytotoxicity assay

Stock solutions (200 mM) of the lyophilised form of the compounds **1**, **4**, **12**, **15**, and **16** were prepared in dimethyl sulfoxide (DMSO) and then stored at −20 °C. For the experimental procedures, the stock solutions were diluted in PBS keeping a final concentration of 5% DMSO. Each solution was used to treat the cells at the established concentrations. Human hepatocarcinoma cell line (Huh7) and human epithelial lung cells (A549) were cultured in Dulbecco’s modified Eagle’s medium (DMEM), supplemented with 10% foetal bovine serum (FBS) (Invitrogen, USA), 100 U/mL penicillin, 100 g/mL streptomycin, 0.22% sodium bicarbonate, and 0.2% HEPES (pH 7.4), in a CO_2_ humid incubation chamber, at 37 °C. The cytotoxicity of compounds was evaluated *in vitro* using a 3–(4,5-dimethylthiazol-2-yl)-2,5-diphenyl tetrazolium bromide (MTT) (USB, Ohio, USA) assay. Monolayers with 6 × 10^4^ cells per well of the Huh7 and A549 cell lines were prepared in, 48-well, cell culture plates. The cells were treated with increasing concentrations of compounds and the times of 24 and 48 h after treatment were analysed. After this time, the cells were incubated for 40 min with 0.5 mg/mL of MTT at 37 °C. Next, the solution was removed and precipitated formazan was diluted in isopropyl alcohol with 40 mM HCl. Absorbance was measured for each well at 570 nm (compounds-treated and control) and 650 nm (background). The percentage of cell viability was calculated as follows: 100% x (absorbance of treated cells) – (background)/(absorbance of untreated cells) – (background).

Statistical analyses were performed using GraphPad Prism 8.0.2 (GraphPad Software, Inc.). Results are presented as means ± standard errors (SEM) and were compared by two-way analysis of variance (ANOVA) and Dunnett's multiple comparisons test *p* values of ≤0.05 were considered significant.

## 3. Results and discussion

### 3.1. Synthesis and characterisation of the organic compounds

Eighteen compounds were used in this study, 8 aromatic guanylhydrazones and 10 aromatic oximes (Figure 1). We chose aromatic and amphipathic compounds to target the interaction with the hydrophobic cleft (α1/α1’ and α2/α2’) in DENVC. We expect the aromatic compounds to interact with the nonpolar exposed surface and with the aromatic backbone at the hydrophobic cleft.

Compounds **1**–**8** were synthesised in the one-step reaction between the correspondent aldehydes and aminoguanidine hydrochloride, using ethanol (95%) as the solvent, HCl as the catalyst, and heating under reflux for 3 − 23 h, generating monocationic compounds obtained in 70–97% yields. The mechanism for those reactions is based on the nucleophilic attack of the –NH_2_ group of aminoguanidine to the –C = O group of the aldehyde, followed by the loss of molecule water and the formation of a double bond, leading to the corresponding guanylhydrazone (Supplementary Information, Scheme S1).

The oximes **9**–**18** were prepared by the reaction of hydroxylamine hydrochloride with the corresponding aldehydes, using ethanol (95%) and distilled water as solvents^26^^,^^55^^,^^56^. After stirring, the product was vacuum filtered and washed with cold distilled water, leading to compounds obtained in 50–98% yields. The time of stirring (1–36 h) and the yield for each compound is described in Supplementary Material with the spectral assignment data. The mechanism for forming oximes is similar to that of guanylhydrazones, based on the nucleophilic attack of the –NH_2_ group of hydroxylamine to the –C=O group of the aldehyde, followed by loss of water and formation of the double bond (Supplementary Information, Scheme S2). Compounds **2**, **4**, **5–11**, **13**, **14**, and **18** are new unpublished agents. Although compounds **1, 3**, **12**, **15**, **16**, and **17** have already been reported in the literature, they have never been tested as DENVC inhibitors. All compounds were characterised by infra-red spectroscopy (IR) and NMR (Supplementary Information, Figures S1–S54).

### 3.2. Ligand-protein interaction studies

**Figure 3.**
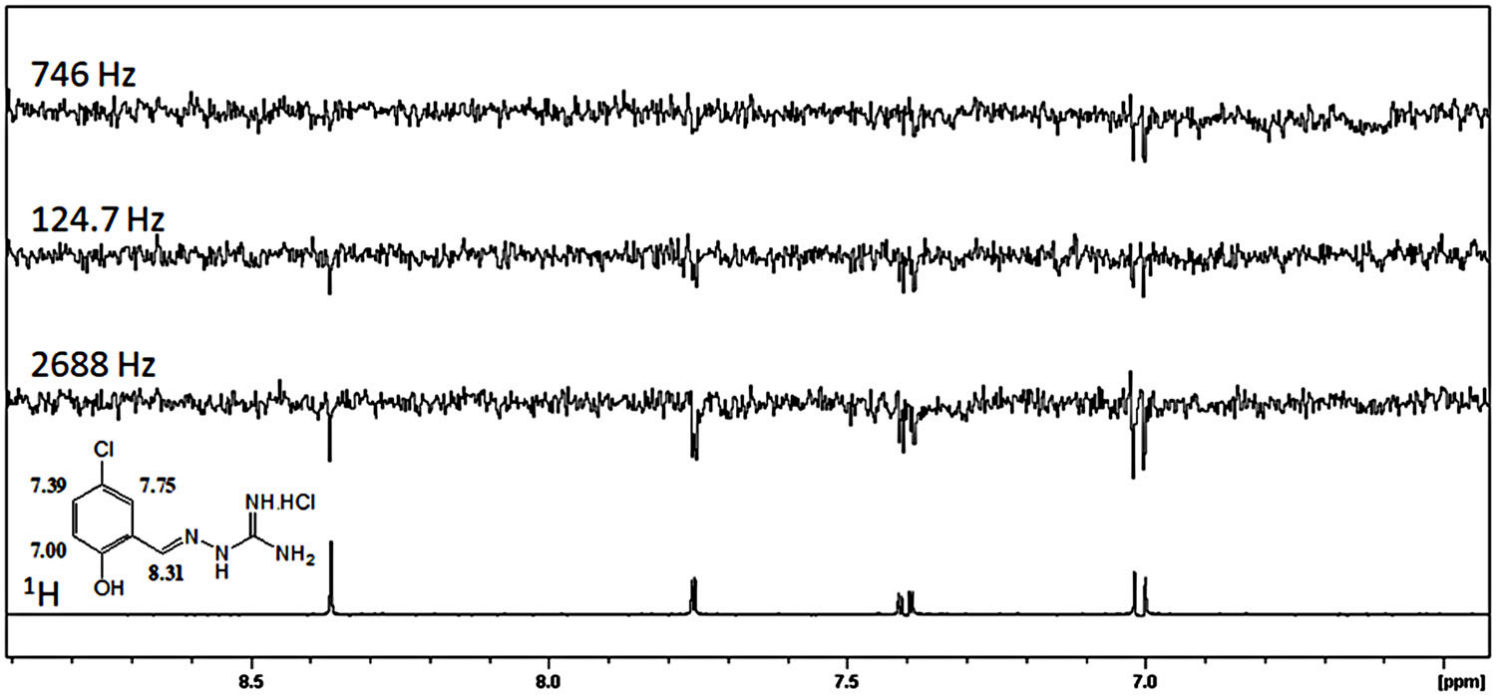
STD spectrum of 1 mM of compound **1** and 10 μM of DENVC in PBS buffer H_2_O/D_2_O (90%/10%) at different frequencies of irradiation (746, 124.7, and 2688 Hz). At the bottom, the reference ^1^H-NMR spectrum of the compound.

To identify the compounds able to bind DENVC, we performed NMR STD experiments. We made pools of 4–5 compounds. The pools were divided according to the observed proton NMR chemical shifts, avoiding overlaps of the signals of the different compounds (Figures S55–S58). The STD spectra for pools 1–4 (Figures S59–S62) showed that compounds **1**, **4**, **11**, **12**, **15,** and **16** binds to DENVC. For these selected ligands, we ran STD experiments for each compound. Compound **11** showed an STD weak signal near the noise (Figure S61) and thus it was not selected for further analysis. The analysis of the compound **1** STD spectrum revealed that all hydrogens are involved in the binding site of DENVC (Figure 3). Similar results were observed for compounds **4**, **12**, **15**, and **16** (Figures S63–S66).

To investigate the intermolecular interaction between DENVC and the selected compounds, we also analysed relaxation parameters^57^. We measured proton relaxation times *T*_1_ and *T*_2_ for the selected compounds in the presence and absence of DENVC (Table 1). Changes in *T*_1_ and *T*_2_ values for compounds **1**, **4**, **12**, **15**, and **16** after the addition of the protein corroborate the STD experiments, showing that all compounds interact with DENVC. As expected for binding, *T*_2_ of all compounds increased in the presence of the protein, with the exception of hydrogen 1 of compounds **1** and **16**. This change could be a result of the presence of conformational exchange at this site in the free ligand that is reduced upon binding. Interestingly, it occurred in the same site for both compounds. The aromatic hydrogens of all compounds presented major *T*_2_ changes, suggesting the aromaticity of these compounds is important for the interaction.

**Table 1.** *T*_1_ and *T*_2_ values for 1 mM of compounds **1**, **4**, **12**, **15**, and **16** in the absence and the presence of 10 uM of DENVC.

Compound		Hydrogen number	*T*_1 ligand_ (s)	*T*_1 ligand+protein_ (s)	*T*_2 ligand_ (ms)	*T*_2 ligand+protein_ (ms)
(1)	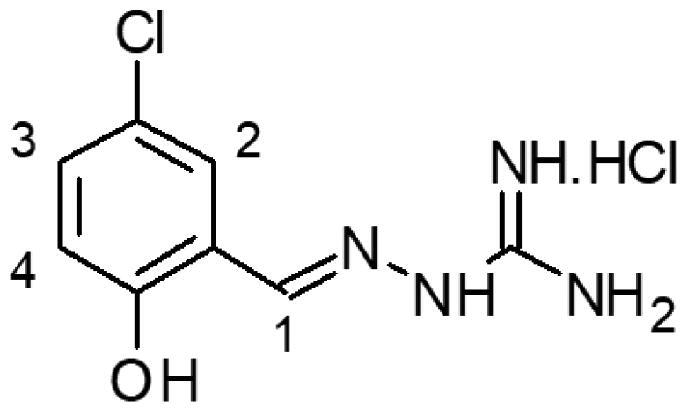	1	1.11	1.69	390.46	441.42
		2	3.09	3.14	602.32	416.99
		3	3.30	2.94	639.53	431.33
		4	1.84	2.39	545.66	471.80
(4)	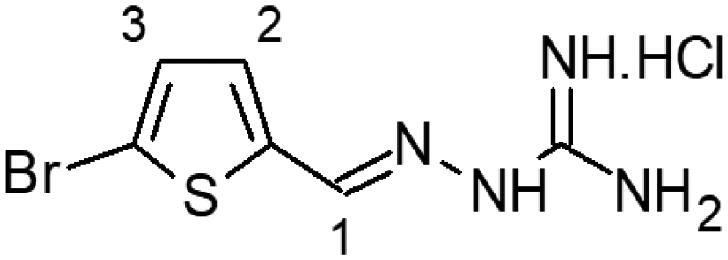	1	2.58	2.13	752.86	501.96
		2	4.60	3.96	1293.60	559.59
		3	3.23	2.84	1062.49	505.95
(12)	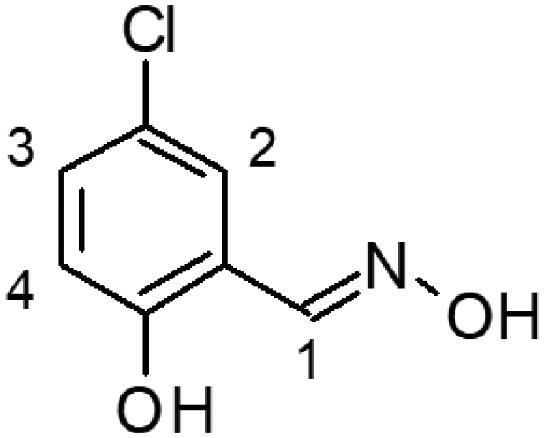	1	3.39	3.36	1186.65	840.66
		2	3.46	3.44	1298.42	787.54
		3	4.26	3.84	1350.42	758.63
		4	3.80	3.76	1312.56	863.21
(15)	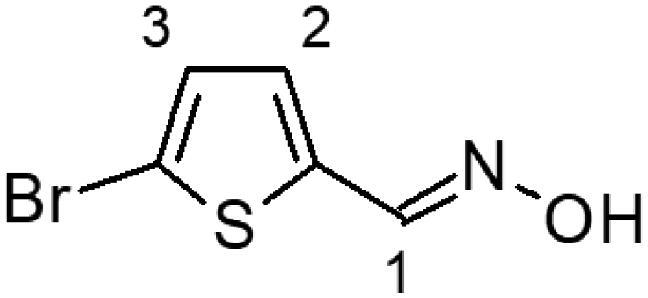	1	4.70	4.12	701.83	655.41
		2	4.19	3.47	1120.43	639.71
		3	4.70	4.12	1171.62	654.88
(16)	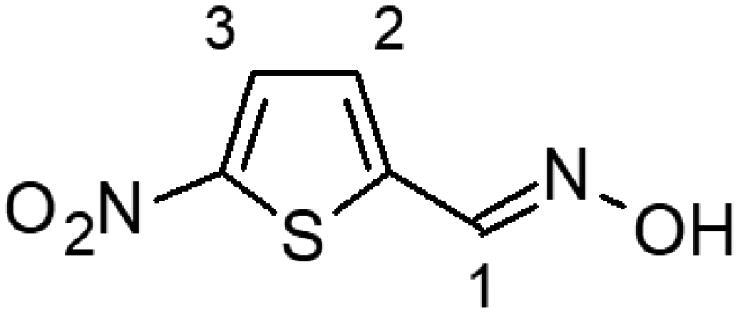	1	5.58	5.82	896.06	1040.06
		2	6.62	5.82	1982.16	1623.02
		3	4.68	4.17	1597.08	1347.14

We also measured the translational diffusion coefficient in the presence and absence of DENVC (Table 2). We observed a decrease in the diffusion coefficient for all compounds in the presence of the protein. Accordingly, such a decrease is expected when a small molecule binds to a high molecular weight biomolecule like a protein^58^.

**Table 2.** Diffusion coefficient (D) for 1 mM of compounds **1**, **4**, **12**, **15**, and **16** in the presence and absence of 10 μM of DENVC.

Compound	D_ligand_ (10^−10^ m^2^/s)	D_ligand+protein_ (10^−10^ m^2^/s)
**1**	7.07	6.26
**4**	7.30	6.95
**12**	7.81	7.17
**15**	7.78	7.22
**16**	7.98	7.80

**Figure 4.**
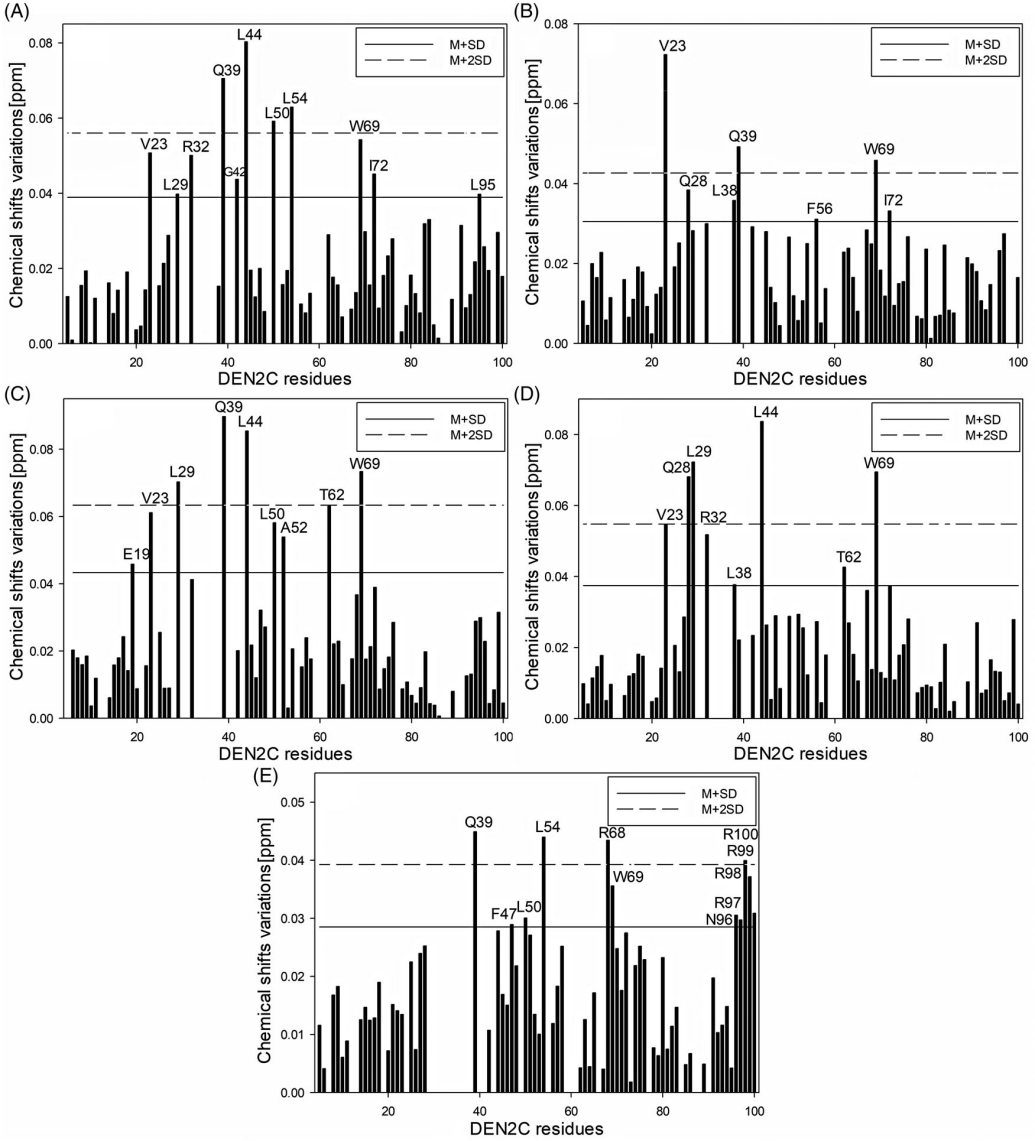
DENVC (200 µM) chemical shift perturbations (CSP) mapped by HSQC ^1^H-^15^N in the absence and presence of 1.8 mM of (A) compound **1**, (B) **4**, (C) **12**, (D) **15**, and (E) **16**. The solid line denotes the average CSP value (M) for all residues plus the standard deviation (SD), and the dashed line indicates the average plus twice standard deviation (2SD).

**Figure 5.**
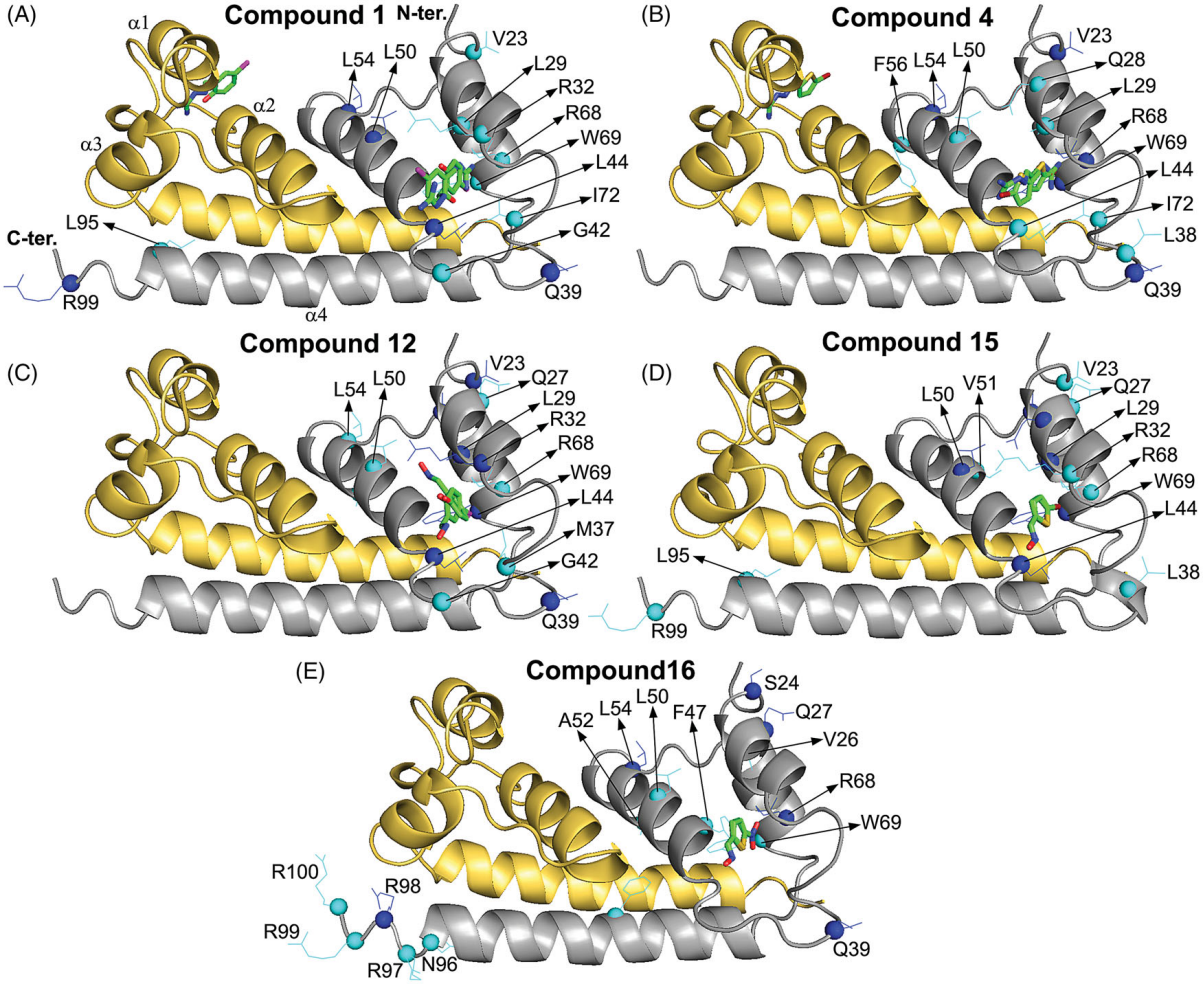
Analysis of the molecular docking results for the structural model of the complex of DENVC with compounds **1** (A), **4** (B), **12** (C), **15** (D), and **16** (E). All compounds are in the symmetric pockets of the hydrophobic cleft in DENVC. The protein is shown as a cartoon model with the monomers coloured in grey for chain A and golden yellow for chain B. The secondary structures of α-helix of the chain B are indicated as α1, α2, α3, and α4. The compounds are denoted as a stick model with carbon, oxygen, nitrogen, sulphur, chlorine, and bromine coloured in green, red, blue, yellow, magenta, and dark red, respectively. The residues with changes in chemical shift (CSP) and intensity higher than M + SD and M + 2SD are denoted as cyan and blue lines/spheres, respectively.

To identify the DENVC residues involved in the binding, we mapped the changes in the signal intensity and chemical shift perturbation (CSP) for all residues by ^1^H-^15^N-HSQC experiments in the presence and absence of the selected compounds (**1**, **4**, **12**, **15**, and **16)** (Figures 4 and S67). The residues showing the highest changes according to the average plus one standard deviation (M + SD) and the average plus two standard deviations (M + 2SD) (Table S1) were selected and represented along with the three-dimensional structure of the DENVC (Figure 5). When we mapped the CSP values and changes in intensity in the protein structure, we observed perturbations at α1, α2, and α3 helices, indicating that the compounds interact in the hydrophobic cleft, the targeted region.

The residues identified by ^1^H-^15^N-HSQC experiments were: for compound **1**, V23, L29, and R32 in α1, Q39, G42, and L44 in the α1–α2 loop, L50 and L54 in α2, R68 and W69 in α3, I72 in the α3–α4 loop, L95 in α4, and R99 in the C-terminal region; for compound **4,** V23, Q28, and L29 in α1, L38, Q39, and L44 in the α1–α2 loop, L50, L54, and F56 in α2, R68 and W69 in α3, and I72 in the α3–α4 loop; for compound **12**, E19 in the N-terminal region, V23, Q27, L29, and R32 in α1, M37, Q39, G42, and L44 in the α1–α2 loop, L50, A52, and L54 in α2, T62 in the α2–α3 loop, and R68 and W69 in α3; for compound **15**, V23, Q27, Q28, L29, and R32 in α1, L38 and L44 in the loop α1–α2, L50 and V51 in α2, T62 in the α2–α3 loop, R68 and W69 in α3, L95 in α4, and R99 in the C-terminal region; and for compound **16**, S24, V26, and Q27 in α1, Q39 and F47 in the α1–α2 loop, L50, A52, and L54 in α2, R68 and W69 in α3, F84 and N96 in α4, and R97, R98, R99, and R100 in the C-terminal region. Residues L50 in α2, the core of the hydrophobic cleft, and R68 and W69 in α3 stand out since they are perturbed in all compounds. Compound **16** promoted singular changes in CSP values of the C-terminal residues. It is worth mentioning that W69, with the side chain in the α2–α3 interface, can be used as a fluorescent probe for investigating the DENVC/selected compound interaction.

We used the intrinsic tryptophan fluorescence of DENVC to measure the binding affinity of each of the selected compounds. All compounds led to fluorescence quenching as a consequence of protein binding. For compounds **1**, **4**, **12**, and **15**, the binding resulted in a redshift of the tryptophan fluorescence spectra, indicating the exposure of W69 to a more polar environment. Conversely, for compound **16**, the binding led to a blue shift in the spectrum, meaning that, in this case, W69 is buried in a more hydrophobic environment^59^ (Figure 2). Interestingly, compound **16** showed significant CSP values in the C-terminal region (Figure 5(E)). The results suggest a specific interaction of these compounds with DENVC, inducing a conformational change in the protein. The dissociation constants ($K_{d}$) were calculated using the change in the maximum emission wavelength ($\lambda_{\max}$) as a function of the ligand concentration (inset in Figure 2). Compounds **1**, **4**, and **12** presented a hyperbolic binding isotherm, displaying $K_{d}$ of 18 ± 4, 20 ± 7, and 23 ± 8 µM, respectively. Compounds **15** and **16** displayed an almost linear isotherm in the measured concentration ranges, probably because they presented affinities in the millimolar range.

**Figure 6.**
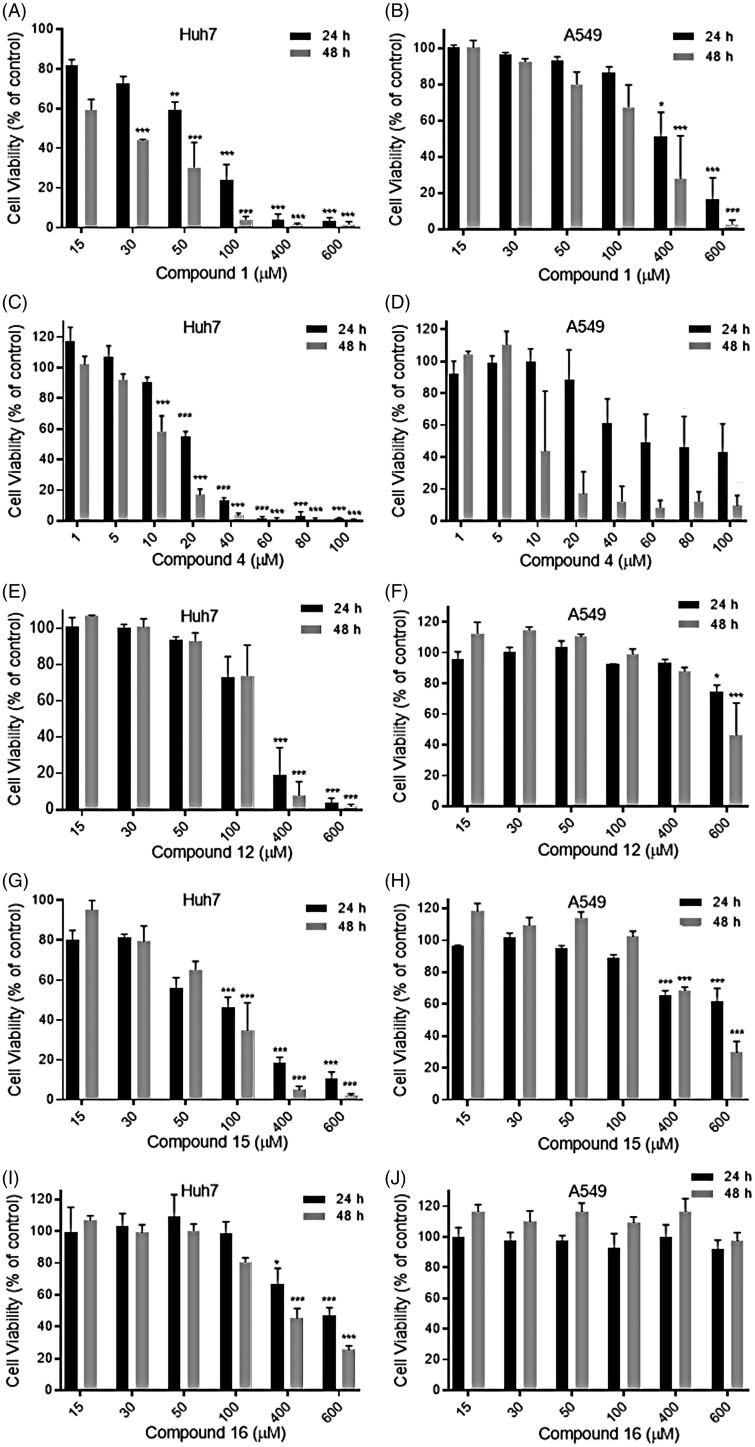
Cytotoxicity of the studied compounds in Huh7 and A549 cells by the MTT assay. The schematic figures of each panel illustrate the cell viability after treatment with increasing concentrations of the respective compounds (**1**, **4**, **12**, **15**, and **16**) in Huh7 (A, C, E, G, and I) and A549 (B, D, F, H, and J) cells, after 24 (black column) and 48 h (grey column). The results are presented as a percentage of the cell viability of the treated cells in relation to the untreated cell maintained in DMEM medium with 0.5% DMSO. Data are represented as mean ± SEM of the results of at least three independent experiments. For statistical analysis, each treated condition was compared with the untreated control at the respective times. Asterisks indicate significant differences between untreated cells and treated cells as follows: *P ≤ 0.05; **P ≤ 0.01; ****P ≤ 0.0001.

The ^1^H-^15^N HSQC experiments indicated that all selected compounds bind to the hydrophobic cleft in DENVC. These experimental results drove the docking calculation of the compounds in the protein binding site. Figure 6 shows the structural models of the DENVC/compounds complexes determined from the docking calculations. The docking poses of the compounds correspond to the lowest energy conformers for the triplicate calculations. It is possible to observe in Figure 5 that the compounds interacted in the two symmetric pockets of the hydrophobic cleft in DENVC. These symmetric pockets are formed by the α1–α2 loop and α3 on both chains of the dimer. An analysis of the non-covalent interactions involved in the structural models revealed the occurrence of hydrophobic contacts and hydrogen bonds in all DENVC/compound complexes, while π-cation interactions appeared exclusively to the compounds **1** and **4** and halogen bonds to the compound **15** (Table S2–S6). In at least two of the three replicas, compound **1** presented hydrophobic interactions with P43 and L46 and formed hydrogen bonds with L29, R41, and L44 (Table S2). The same hydrophobic contacts were observed for compound **4** (Table S3), but the hydrogen bond was only recurrent with L29. Compound **12** showed the highest number of hydrophobic interactions, being formed with L29, R32, F33, L44, L46, and F47, while only one hydrogen bond was formed with R41 (Table S4). Compounds **15** and **16** established hydrogen bonds with R41 and L44 in two of three docking runs (Tables S5 and S6). The hydrophobic contacts were remarkably similar for these compounds, being formed with L29, R32, and L46, except F47 that was observed only for compound **16**. It is possible to see that R41 and L46 are important residues for the binding of all compounds in the symmetric pockets of DENVC hydrophobic cleft, the former involved in hydrogen bonds, and the last in hydrophobic interaction. It is worth mentioning that R41 also is the main residue responsible for the formation of π-cation interactions with compounds **1** and **4** (Tables S2 and S3). Interestingly, the halogen bond is significant and a recurring interaction in triplicate docking calculations, which is established between the R68 and bromine atom of compound **15** (Table S5).

### 3.3. Pharmacokinetic and toxicological properties

The calculated pharmacokinetic parameters (Table 3) show that the 5 selected compounds are candidates as fragment leads. The solubility (cLogS) for all compounds was found in an acceptable range (<4)^60^. The cLogP parameter, which is directly related to a more favourable drug-likeness profile when ≤5^61^^,^^62^, shows that all compounds meet this rule. They all have molecular weights ≤500 g/mol, which means that transportation and absorption are easier than heavy molecules^62^. Regarding the polar surface area criterium (PSA ≤140 Å^2^ for oral bioavailability^62^ or 90 Å^2^ for the cellular permeability^61^, all compounds showed acceptable results, with exception of compounds **1**, **4**, and **16** for cellular permeability. The results for donor sites (nOHNH) and hydrogen bonding acceptors (nOH), below 10 and 5, respectively, show that all compounds meet the rule for oral bioavailability^62^ and for crossing the blood-brain barrier (central nervous system, CNS)^61^. They all have rotatable bond ≤5, which is related to the binding potency and penetration into the membrane^62^. Thus, the results showed that none of the studied compounds presented more than three violations to Lipinski’s rule, suggesting they are suitable candidates for oral drugs.

**Table 3.** *In silico* pharmacokinetic parameters and toxicological properties of compounds.

Compound	MW	PSA	cLogP	cLogS	nOH	nOHNH	RBs	Toxicological
**1**	249.10	94.49	1.00	−2.43	4	5	2	NT, NM, NR, NI
**4**	283.58	102.50	1.53	−2.86	3	4	2	NT, NM, NR, NI
**12**	171.58	52.82	2.47	−2.76	2	3	1	NT, NM, NR, NI
**15**	206.06	60.83	3.01	−3.18	1	2	1	NT, NM, NR, NI
**16**	173.17	106.65	1.35	−2.94	1	5	2	NT, NM, NR, NI

MW: molecular weight (g/mol); PSA: polar surface area in Å^2^; cLogP: bipartition coefficient; cLogS: solubility; nOH: hydrogen bonding donors; nOHNH: hydrogen bonding acceptors; RBs: rotatable bonds; NT: non-tumorigenic; NM: non-mutagenic; NR: non-reproductive effective; NI: non-irritant.

The toxicity risk calculations search for substructures within the chemical structure being indicative of specific toxicity according to a reference database (Registry of Toxic Effects of Chemical Substances database – RTECS), which covers compounds of different toxicity classes^49^^,^^63^. The absence of risky fragments suggests a low risk concerning the toxicity class under investigation^63^. For these studies, the toxicity classes related to mutagenic, tumorigenic, or irritant effects or being associated with reproductive effects are considered. The results for all compounds showed they do not present any toxicological risks for those classes. It is important to emphasise that these results do not eliminate the need for traditional toxicological tests.

We evaluated the cytotoxicity of the compounds on Huh7 and A549 cells. Both cell lines were chosen because they are widely used in studies of DENV infection and replication. The MTT assay was performed after 24 and 48 h of treatment with increasing concentrations of each compound (Figure 6, AJ). Cell lines maintained in culture medium with 0.5% DMSO were used as controls since this is the final DMSO concentration in the medium after the addition of the compounds. The viability results for cell treatment with each concentration of the compounds are expressed as a percentage related to cells treated with only DMSO, which did not significantly affect the cell viability. In general, the compounds were more toxic to Huh7 cells than to A549 cells. Compound **1** was very toxic to HuH7 cells even at low concentrations, leading to about 50% loss of viability at 30 µM after 48 h (Figure 6(A)). On the other hand, for A549, the compound was toxic only at much higher concentrations, such as 400 µM (Figure 6(B)). The most cytotoxic compound for both cells studied was compound **4**, with concentrations above 20 µM being highly toxic (Figure 6(C–D)). On the other hand, compounds **12**, **15**, and **16** showed much more promising results (Figure 6(E–J)). Compounds **15** were toxic only at concentrations above 100 or 400 µM to HuH7 or A549, respectively (Figure 6(G–H)), while compounds **12** and **16** were toxic to HuH7 only at concentrations above 400 µM (Figure 6(E,I)), with even better results to A549 (Figure 6(F,J)). Compound **16** was non-toxic to A549 cells (Figure 6(J)) at all the concentrations tested and showed low cytotoxicity up to 100 µM in Huh7 cells, with cell viability of 98% and 80% after 24 and 48 h treatment, respectively (Figure 6(I)). Therefore, among the tested compounds, compound 16 may be seen as the best candidate to explore a possible antiviral activity against DENV in future trials.

## 4. Conclusions

Here we presented a targeted screen of small aromatic compounds to the hydrophobic cleft (α1-α1' and α2-α2') of DENVC. The strategy was successful, enabling the description of new fragment leads that binds to the hydrophobic cleft, and this new series of compounds may be used as promising leads for dengue therapy. The advantages of these compounds include physical properties compatible with desirable pharmacokinetic parameters, such as low toxicity, simple synthetic procedure, and evidence of binding at a micromolar concentration to DENVC. Remarkably, STD-selected compounds elicited conformational changes upon DENVC binding. For compounds **1**, **4**, **12**, and **15**, the tryptophan residue (W69) is more solvent-exposed, possibly making the hydrophobic core looser, and for compound **16**, W69 is less solvent-exposed, hidden in a more rigid hydrophobic core. NMR-derived docking calculations suggest that the selected compounds are located in asymmetric binding sites formed by α1, α2, and α3 in the hydrophobic cleft, where W69 is at the centre. These studies should allow the development of antiviral analogs targeting the hydrophobic cleft of the DENVC and possibly blocking its interaction with lipid droplets.

## Supplementary Material

Supplemental Material
