# Supplementary material for: Searching for drug leads targeted to the hydrophobic cleft of dengue virus capsid protein
Source: J Enzyme Inhib Med Chem. 2021 Dec 11;37(1):287–98. doi: 10.1080/14756366.2021.2004591 (PMC8667904; doi:10.1080/14756366.2021.2004591)
Supplement: Supplemental Material [file IENZ_A_2004591_SM5094.pdf]

Supporting Information for:

Searching for drug leads targeted to the hydrophobic cleft of dengue virus capsid protein

Liliane O. Ortlieb[a,c], Ícaro P. Caruso[b,c], Nathane C. Mebus-Antunes[d], Andrea T. Da Poian[d], Elaine da C. Petronilho[a], José Daniel Figueroa-Villar[a], Claudia J. Nascimento[e,*], Fabio C. L. Almeida[c,*]

[a]Department of Chemistry, Military Institute of Engineering (IME), 22290-270, Rio de Janeiro, RJ, Brazil;

[b]Multiuser Center for Biomolecular Innovation (CMIB) and Department of Physics, Institute of Biosciences, Letters and Exact Sciences (IBILCE), São Paulo State University (UNESP), 15054-000, São José do Rio Preto, SP, Brazil;

[c]Institute of Medical Biochemistry Leopoldo de Meis (IBqM) and National Center for Structural Biology and Bioimaging (CENABIO), Federal University of Rio de Janeiro (UFRJ), 21941-590, Rio de Janeiro, RJ, Brazil;

[d]Institute of Medical Biochemistry Leopoldo de Meis (IBqM), Federal University of Rio de Janeiro (UFRJ), 21941-590, Rio de Janeiro, RJ, Brazil;

[e]Department of Natural Sciences, Institute of Biosciences, Federal University of the State of Rio de Janeiro (UNIRIO), 22.290-240, Rio de Janeiro, RJ, Brazil.

*Correspondence should be addressed to F.C.L.A. and C.J.N.

e-mail: falmeida@bioqmed.ufrj.br and claudia.nascimento@unirio.br

Tel.: +55-21-31042326

The data of yield, melting point, reaction time, and spectral assignment for each compound are presented below. Chemical shifts are given in ppm (δ) and coupling constants (J) values in Hertz. Abbreviations for ^{1}H NMR data are as follows: s (singlet); d (doublet); dd (doublet of doublets) and b (broad signal).

5-chloro-salicylaldehyde-guanylhydrazone (1): Yield 91.1% (reflux and stirring for 21 h); White solid, mp: 275-276 °C (Lit. 286-287) [1]. IR (KBr) ν_{max}/cm^{-1}: 3429, 3321, 3145, 1683, 1627, 1276, 659; ^{1}H NMR (600 MHz, DMSO-d_6): δ/ppm 6.98 (d, J = 8.7 Hz, 1H), 7.27 (dd, J = 8.7 e 2.6 Hz, 1H), 7.78 (b, 4H), 8.07 (d, J = 2.6 Hz, 1H), 8.38 (s, 1H), 10.57 (s, 1H), 12.08 (s, 1H); ^{13}C NMR (150 MHz, DMSO-d_6): δ/ppm 118.01, 121.38, 123.36, 125.53, 131.19, 141.64, 155.31, 155.44.

5-bromo-pyridine-2-guanylhydrazone (2): Yield 96.4% (reflux and stirring for 4 h); White solid, mp: 225-227 °C. IR (KBr) ν_{max}/cm^{-1}: 3433, 3161, 1676, 1612, 1526, 1475, 707; ^{1}H NMR (600 MHz, DMSO-d_6): δ/ppm 8.02 (s, 4H); 8.17 (s, 1H); 8.18 (dd, *J* = 2.2 e 8.5 Hz, 1H); 8.28 (d, *J* = 8.5 Hz, 1H); 8.74 (d, *J* = 2.2 Hz, 1H); 12.44 (s, 1H); ^{13}C NMR (150 MHz, DMSO-d_6): δ/ppm 121.31; 122.34; 139.56; 145.71; 150.12; 151.24; 155.41.

Thiophene-2-guanylhydrazone (3): Yield 70% (reflux and stirring for 8 h); Pale yellow solid, mp: 250-252°C (Lit. 107-109) [2]. IR (KBr) ν_{max}/cm^{-1}: 3268, 3158, 1670, 1620, 1543. ^{1}H NMR (600 MHz, DMSO-d_6): δ/ppm 7.14 (d, *J* = 3.8 Hz, 1H); 7.54 (d, *J* = 3.8 Hz, 1H); 7.69 (s, 4H); 7.72 (d, *J* = 3.8 Hz, 1H); 8.41 (s, 1H); 12.11 (s, 1H). ^{13}C NMR (150 MHz, DMSO-d_6): δ/ppm 127.93; 129.84; 131.72; 137.57; 142.19; 155.11.

5-bromo-thiophene-2-guanylhydrazone (4): Yield 86.5% (reflux and stirring for 6 h); White solid, mp: 148-149 °C. IR (KBr) ν_{max}/cm^{-1}: 3398, 3072, 1662, 1546, 1203, 705. ^{1}H NMR (600 MHz, DMSO-d_6): δ/ppm 7.27 (d, J = 3.9 Hz, 1H), 7.37 (d, J = 3.9 Hz, 1H), 7.73 (s, 4H), 8.33 (s, 1H), 12.22 (s, 1H); ^{13}C-coupled NMR (150 MHz, DMSO-d_6): δ/ppm 115.61, 131.39, 132.22, 139.57, 141.19, 155.08.

5-nitro-thiophene-2-guanylhydrazone (5): Yield 97% (reflux and stirring for 3 h); Yellow solid, mp: 246-248 °C. IR (KBr) ν_{max}/cm^{-1}: 3387, 3176, 1683, 1618, 1527, 1489, 1340. ^{1}H NMR (600 MHz, DMSO-d_6): δ/ppm 7.62 (d, *J* = 3.9 Hz, 1H); 7.93 (s, 4H); 8.11 (d, *J* = 3.9 Hz, 1H); 8.44 (s, 1H); 12.56 (s, 1H). ^{13}C NMR (150 MHz, DMSO-d_6): δ/ppm 130.22; 140.36; 145.08; 151.56; 155.20.

1-methyl-imidazole-5-guanylhydrazone (6): Yield 87% (reflux and stirring for 23 h); White solid, mp: 234-236 °C. IR (KBr) ν_{max}/cm^{-1}: 3431, 3157, 1672, 1629, 1500, 1219. ^{1}H NMR (600 MHz, DMSO-d_6): δ/ppm 3.86 (s, 3H), 7.54 (s, 1H), 7.65 (s, 4H), 8.01 (s, 1H), 8.21 (s, 1H), 12.12 (s, 1H).; ^{13}C NMR (150 MHz, DMSO-d_6): δ/ppm 34.20; 126.57; 133.20; 137.71; 141.77, 155.21.

4-methyl-imidazole-5-guanylhydrazone (7): Yield 84.4% (reflux and stirring for 16 h); White solid, mp: 253-255 °C. IR (KBr) ν_{max}/cm^{-1}: 3469, 3360, 3143, 1687, 1604, 1560. ^{1}H NMR (600 MHz, DMSO-

d$_6$): δ/ppm 2.28 (s, 3H), 7.51 (s, 1H), 7.69 (s, 4H), 7.84 (s, 1H), 8.14 (s, 1H) 11.82 (s, 1H); ^{13}C NMR (150 MHz, DMSO-*d*$_6$): δ/ppm 11.44; 130.78; 132.24; 134.88; 136.32; 155.27.

4-(4-morpholinyl)benzyl-guanylhydrazone (8): Yield 86.7% (reflux and stirring for 22 h); Brown solid, mp: 269-270 °C. IR (KBr) ν_{max}/cm^{-1}: 3454, 3170, 1680, 1616, 1587, 1232, 1186. ^{1}H NMR (600 MHz, DMSO-*d*$_6$): δ/ppm 3.20 (t, *J* = 4.9 Hz, 4H); 3.72 (t, *J* = 4.9 Hz, 4H); 6.97 (d, *J* = 8.9 Hz, 2H); 7.68 (d, *J* = 8.9 Hz, 2H); 7.68 (s, 4H); 8.03 (s, 1H); 11.83 (s, 1H); ^{13}C NMR (150 MHz, DMSO-*d*$_6$): δ/ppm 47.42; 65.88; 114.18; 123.66; 128.80; 146.92; 152.31; 155.19.

2,4-dinitro-benzyloxime (9): Yield 98% (reflux and stirring for 3 h); Brown solid, mp: 121-123 °C. IR (KBr) ν_{max}/cm^{-1}: 3232, 3079, 1605, 1528, 1444, 1337, 835, 743. ^{1}H NMR (600 MHz, DMSO-*d*$_6$): δ/ppm 8.14 (d, *J* = 2.3, 1H); 8.48 (s, 1H); 8.52 (dd, *J* = 2.3 e 8.6 Hz, 1H); 8.76 (d, *J* = 8.6, 1H); 12.31 (s, 1H).; ^{13}C NMR (150 MHz, DMSO-*d*$_6$): δ/ppm 120.20; 127.54; 129.76; 132.74; 143.90; 147.23.

2-chloro-3,6-difluoro-benzyloxime (10): Yield 63.5% (reflux and stirring for 1 h); White solid, mp: 138-140 °C. IR (KBr) ν_{max}/cm^{-1}: 3468, 3329, 1581, 1481, 991, 815. ^{1}H NMR (600 MHz, DMSO-*d*$_6$): δ/ppm 7.38 (td, 4.2 e 9.4 Hz, 1H), 7.51 (td, 4.6 e 8.9 Hz, 1H), 8.22 (s, 1H), 12.03 (s,1H); ^{13}C-coupled NMR (150 MHz, DMSO-*d*$_6$): δ/ppm 116.12; 117.38; 120.02; 120.69; 141.03; 154.20; 155.93.

2-chloro-5-nitro-benzyloxime (11): Yield 69.1% (reflux and stirring for 1 h); White solid, mp: 160-161 °C. IR (KBr) ν_{max}/cm^{-1}: 3479, 3105, 1606, 1568, 1529, 1361, 956, 738. ^{1}H NMR (600 MHz, DMSO-*d*$_6$): δ/ppm 7.80 (d, *J* = 8.8 Hz, 1H), 8.19 (dd, *J* = 8.8, e 2.7 Hz, 1H), 8.38 (s,1H), 8.50 (d, *J* = 2.7 Hz, 1H), 12.12 (s,1H); ^{13}C NMR (150 MHz, DMSO-*d*$_6$): δ/ppm 126.29; 130.02; 136.65; 136.94; 143.48; 148.52; 151.73.

2-hydroxi-5-chloro-benzyloxime (12): Yield 61% (reflux and stirring for 18 h); White solid, mp: 120-122 °C [3]. IR (KBr) ν_{max}/cm^{-1}: 3417, 3115, 1629, 1571, 1481, 817, 705, 646. ^{1}H NMR (600 MHz, DMSO-*d*$_6$): δ/ppm 6.90 (d, J = 8.6 Hz, 1H), 7.23 (dd, J = 2.4 e 8.6 Hz, 1H), 7.50 (d, J = 2.4 Hz, 1H), 8.28 (s, 1H), 10.30 (s, 1H), 11.50 (s, 1H); ^{13}C NMR (150 MHz, DMSO-*d*$_6$): δ/ppm 117.88, 120.19, 123.02, 126.37, 129.98, 145.74, 154.71.

6-chloro-pyridine-3-oxime (13): Yield 50% (reflux and stirring for 2 h); White solid, mp: 183-184 °C. IR (KBr) ν_{max}/cm^{-1}: 3155, 3076, 1587, 1560, 1496, 837, 642. ^{1}H NMR (600 MHz, DMSO-*d*$_6$): δ/ppm 7.54 (d, *J* = 8.3 Hz, 1H), 8.05 (dd, *J* = 2.3 e 8.3 Hz, 1H), 8.20 (s,1H), 8.57 (d, *J* = 2.3 Hz, 1H), 11.65 (s, 1H); ^{13}C NMR (150 MHz, DMSO-*d*$_6$): δ/ppm 124.53; 128.57; 136.38; 144.71; 148.01; 150.42.

5-bromo-pyridine-2-oxime (14): Yield 50% (reflux and stirring for 24 h); White solid, mp: 210-212 °C. IR (KBr) ν_{max}/cm^{-1}: 3172, 2879, 1575, 1502, 837, 705, 636. ^{1}H NMR (600 MHz, DMSO-*d*$_6$): δ/ppm 7.73 (d, *J* = 8.4 Hz, 1H), 8.05 (s,1H), 8.06 (dd, *J* = 2.3 e 8.4 Hz, 1H), 8.70 (d, *J* = 2.3 Hz, 1H), 11.84 (s,1H).; ^{13}C NMR (150 MHz, DMSO-*d*$_6$): δ/ppm 120.24; 121.39; 139.49; 148.06; 150.15; 150.89.

5-bromo-thiophene-2-oxime (15): Yield 80.6% (reflux and stirring for 36 h); White solid, mp: 148-149 °C [4]. IR (KBr) ν_{max}/cm^{-1}: 3192, 2825, 1627, 1413, 806, 669. ^{1}H NMR (600 MHz, DMSO-d_6): δ/ppm 7.26 (d, J = 4.0 Hz, 1H), 7.30 (d, J = 4.0 Hz, 1H), 7.82 (s, 1H), 12.13 (s, 1H); ^{13}C NMR (150 MHz, DMSO-d_6): δ/ppm 117.38, 129.39, 131.12, 132.43, 139.62.

5-nitro-thiophene-2-oxime (16): Yield 64% (reflux and stirring for 3 h); Yellow solid, mp: 130-131 °C (Lit. 130 °C) [5]. IR (KBr) ν_{max}/cm^{-1}: 3176, 3010, 1624, 1535, 1502, 1330, 823. ^{1}H NMR (600 MHz, DMSO-d_6): δ/ppm 7.52 (d, J = 4.3 Hz, 1H), 8.10 (s, 1H), 8.12 (d, J = 4.3 Hz, 1H), 13.12 (s, 1H). ^{13}C NMR (150 MHz, DMSO-d_6): δ/ppm 128.70, 129.97, 135.48, 139.67, 152.38.

5-nitro-furfural-2-oxime (17): Yield 60% (reflux and stirring for 5 h); Yellow solid, mp: 148150 °C [6]. IR (KBr) ν_{max}/cm^{-1}: 3176, 3032, 1635, 1575, 1533, 1354, 1251, 1029. ^{1}H NMR (600 MHz, DMSO-d_6): δ/ppm 7.40 (d, J = 3.8 Hz, 1H), 7.75 (d, J = 3.8 Hz, 1H), 7.77 (s, 1H), 12.71 (s,1H). ^{13}C NMR (150 MHz, DMSO-d_6): δ/ppm 114.29; 118.76; 134.30; 146.52; 150.43.

4-(4-morphonylyl)benzyloxime (18): Yield 52% (reflux and stirring for 2 h); White solid, mp: 193-195 °C. IR (KBr) ν_{max}/cm^{-1}: 3381, 3248, 1614, 1521, 1240, 1064, 821. ^{1}H NMR (600 MHz, DMSO-d_6): δ/ppm 3.13 (t, J = 4.7, 4H); 3.71 (t, J = 4.7, 4H); 6.93 (d, J = 8.6, 2H); 7.42 (d, J = 8.6, 2H); 7.99 (s, 1H); 10.80 (s, 1H).; ^{13}C NMR (150 MHz, DMSO-d_6): δ/ppm 47.72; 65.95; 114.56; 123.52; 127.36; 147.84; 151.60.

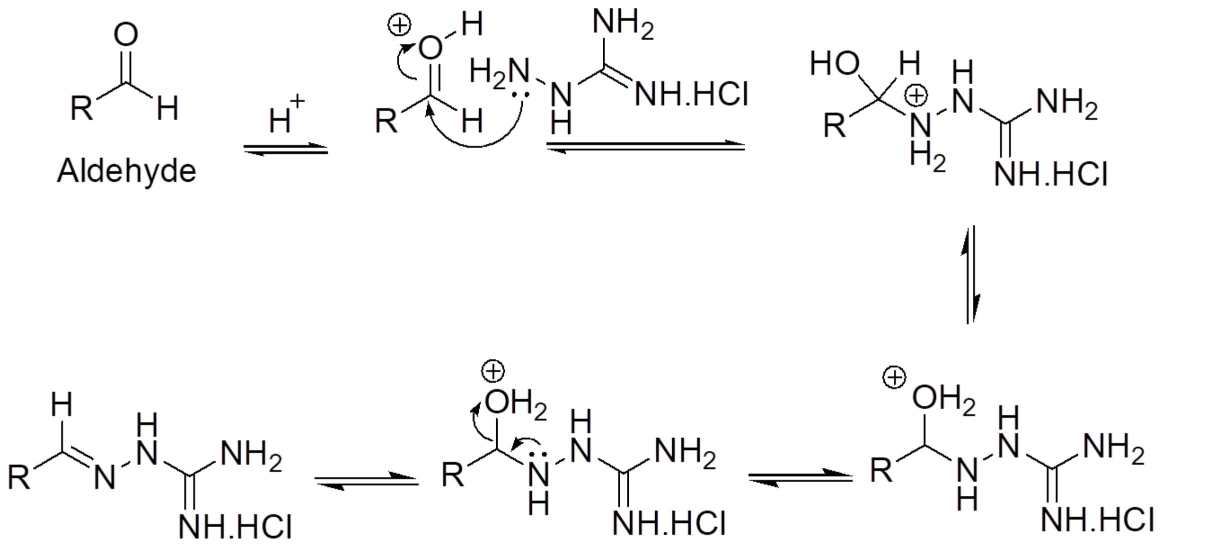

Scheme S1 – Proposed mechanism for the synthesis of the guanylhydrazones. Reagents and conditions (1 – 8): Aminoguanidine hydrochloride (1.2 mmols) dissolved in 20 mL of 95% ethanol, the corresponding aldehyde (1 mmol) and 2 drops of HCl (0.6 M). The solution was kept under reflux and stirring. The solid obtained after eliminating the solvent under vacuum was solubilized in distilled water and extracted with dichloromethane (5 x 20 mL). The product was recrystallized from ethanol.

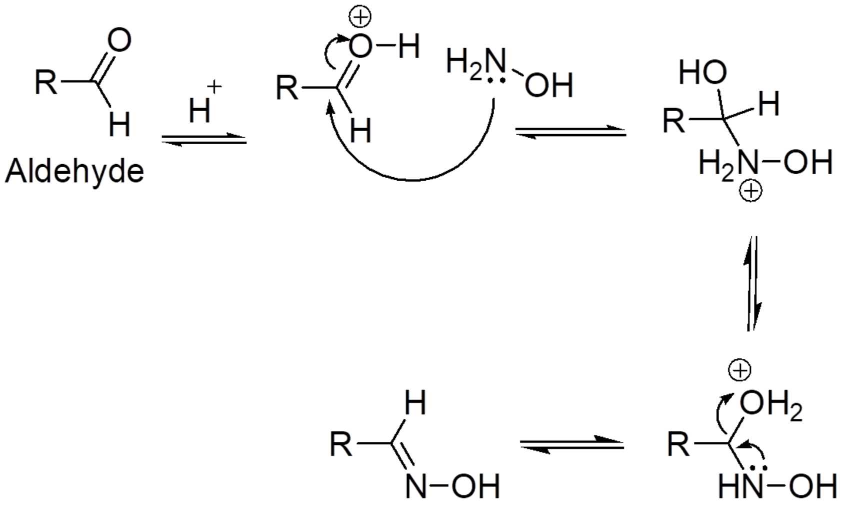

Scheme S2 – Proposed mechanism for the synthesis of the oximes. Reagents and conditions (9 – 18): Hydroxylamine hydrochloride (4 mmols) dissolved in a mixture of 10 mL of ethanol and 3 mL of water was placed in a round bottom flask. The corresponding aldehyde (2 mmols) was added to the solution, which was kept under stirring. The solid obtained after eliminating the solvent under vacuum was washed with distilled cool water. The oximes were recrystallized from ethanol or methanol.

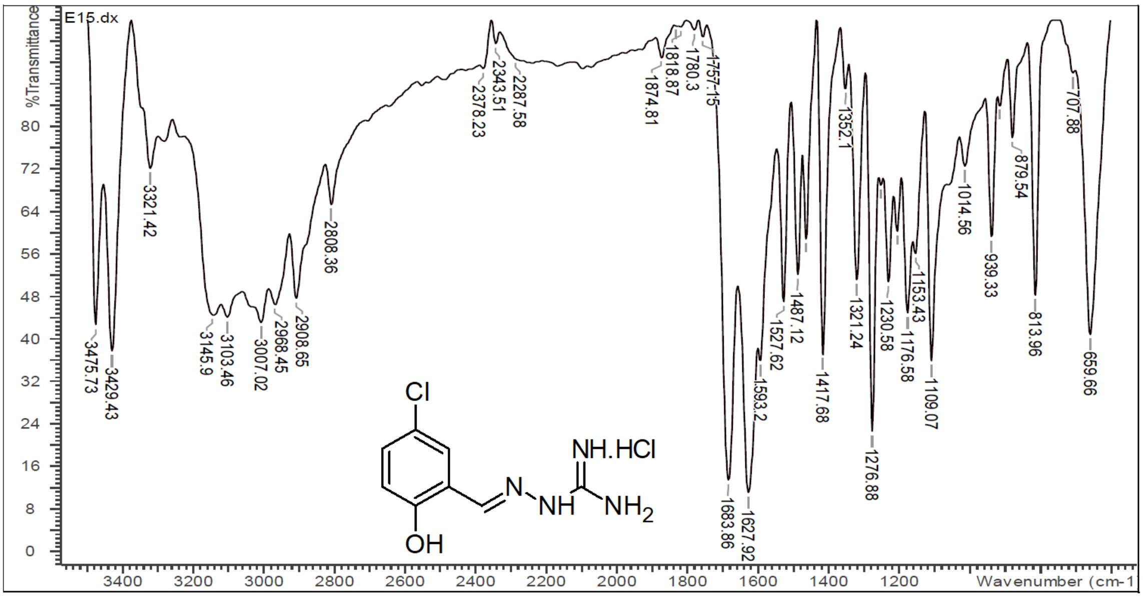

Figure S1 – IR spectrum of the compound 1.

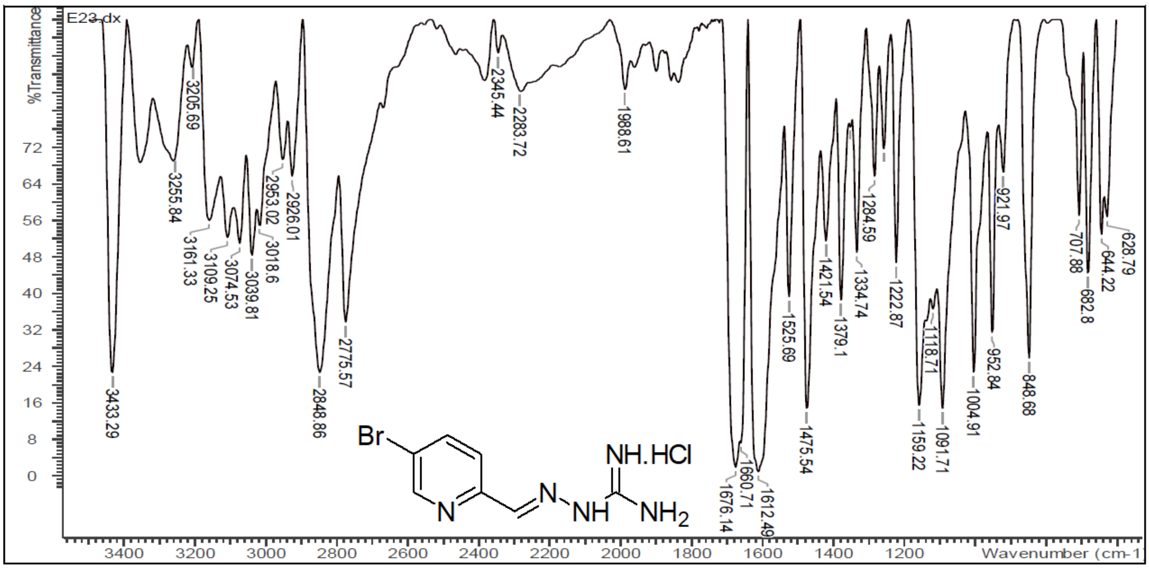

Figure S2 – IR spectrum of the compound 2.

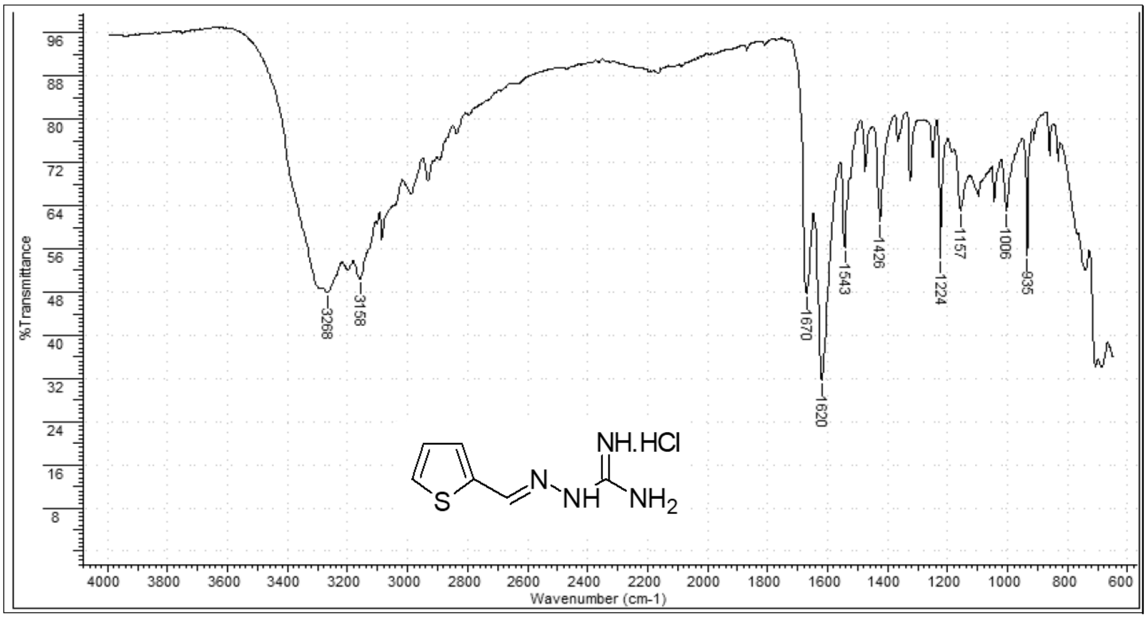

Figure S3 – IR spectrum of the compound 3.

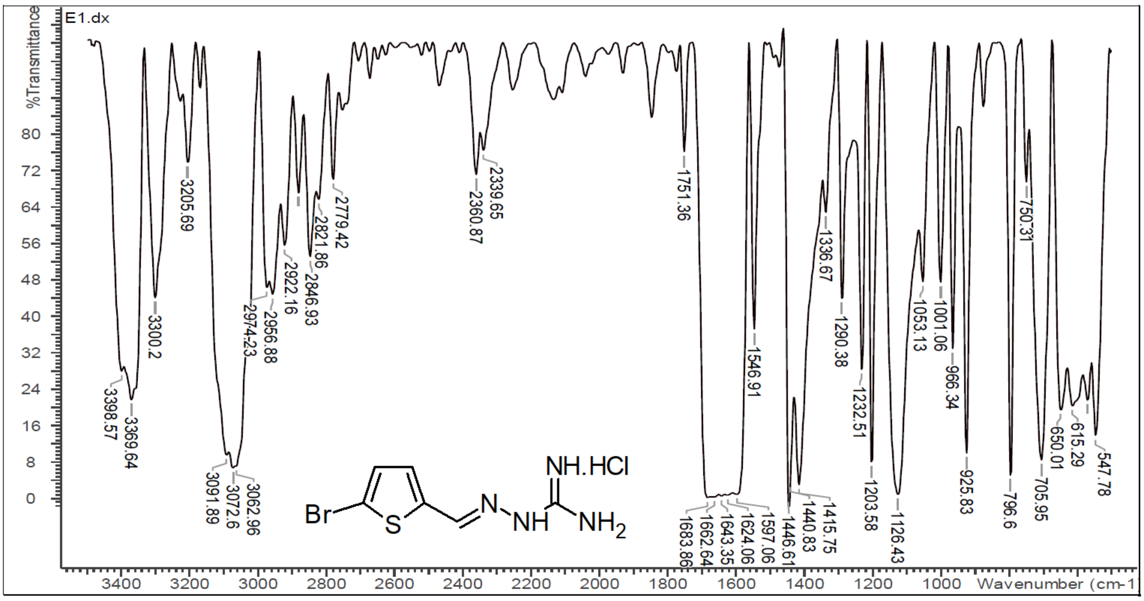

Figure S4 – IR spectrum of the compound 4.

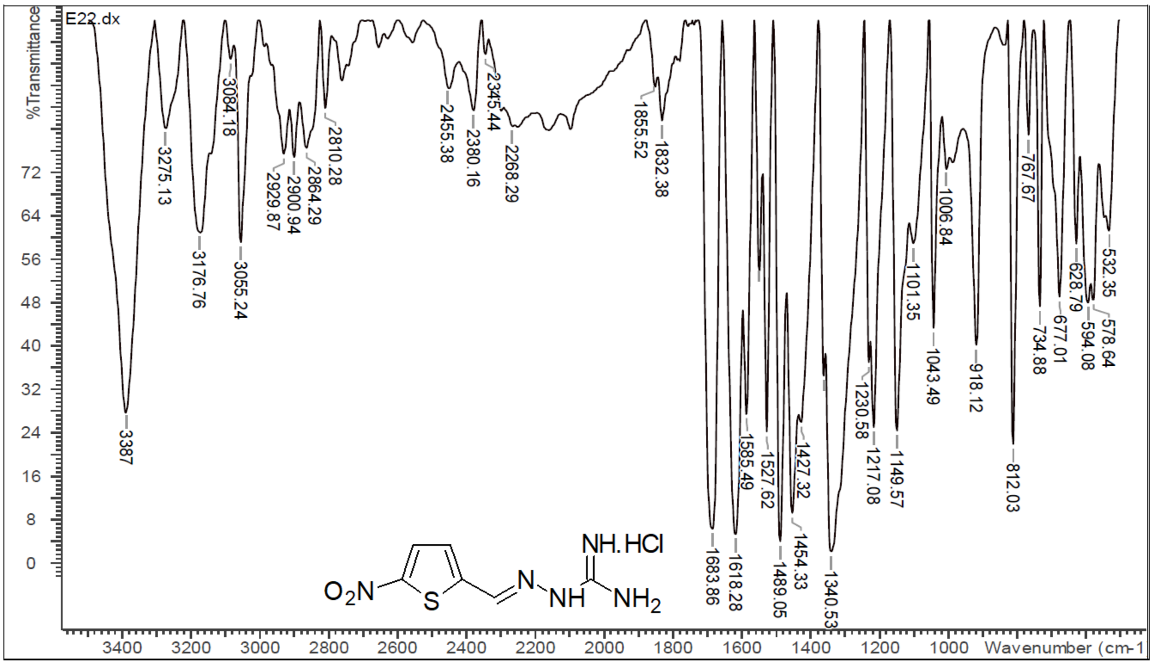

Figure S5 – IR spectrum of the compound 5.

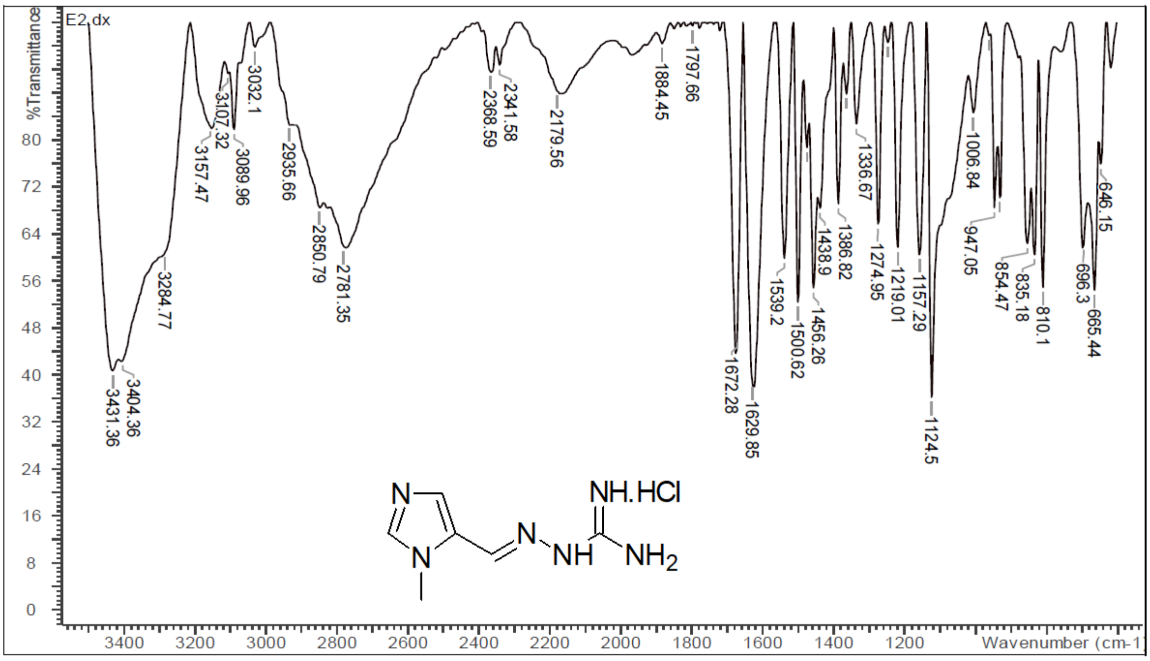

Figure S6 – IR spectrum of the compound 6.

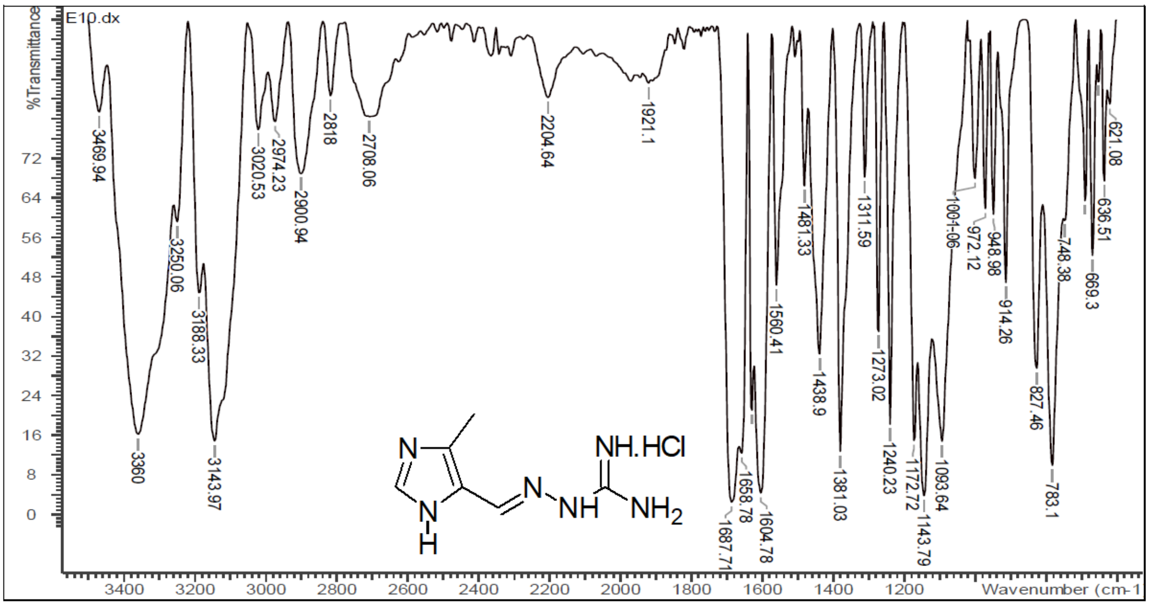

Figure S7 – IR spectrum of the compound 7.

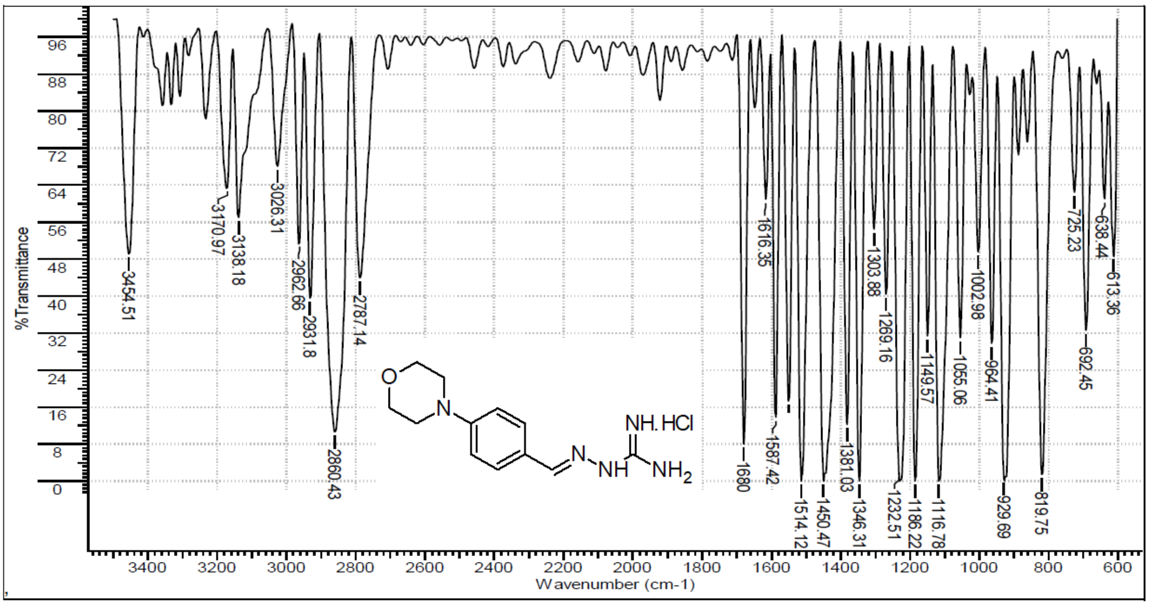

Figure S8 – IR spectrum of the compound 8.

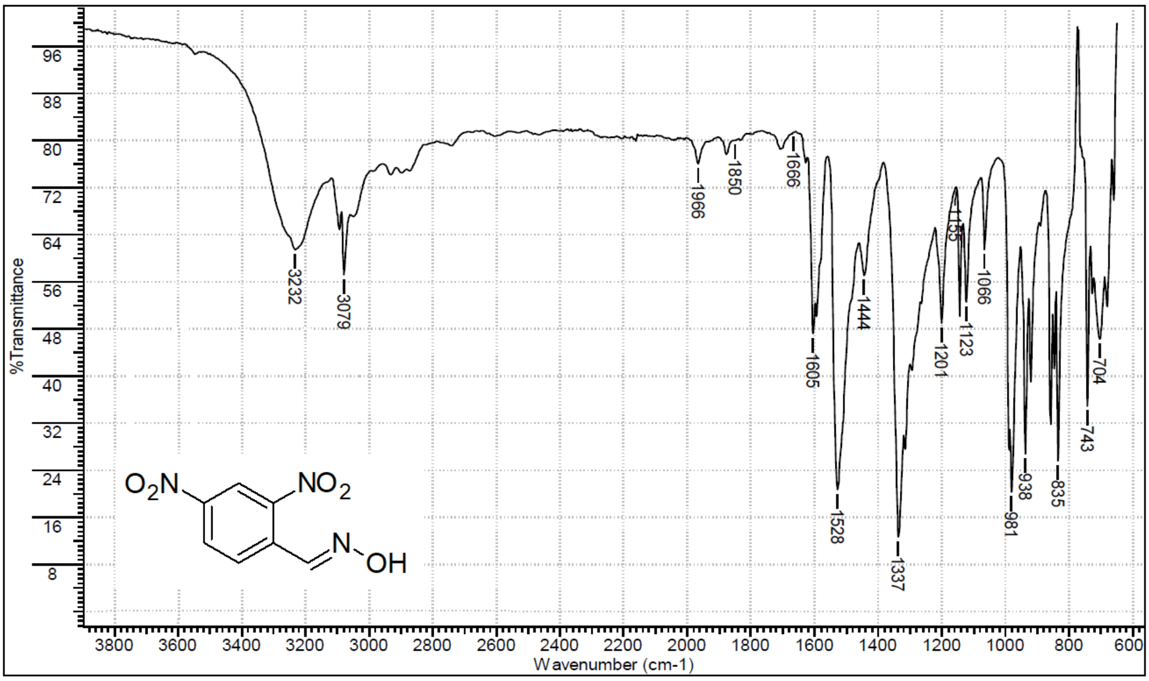

Figure S9 – IR spectrum of the compound 9.

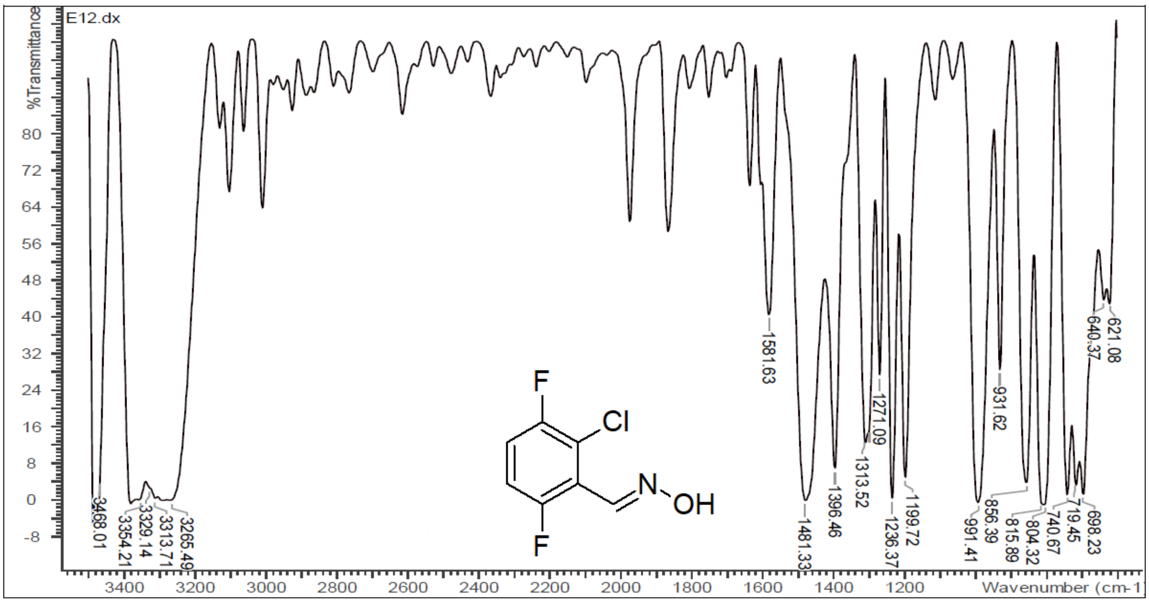

Figure S10 – IR spectrum of the compound 10.

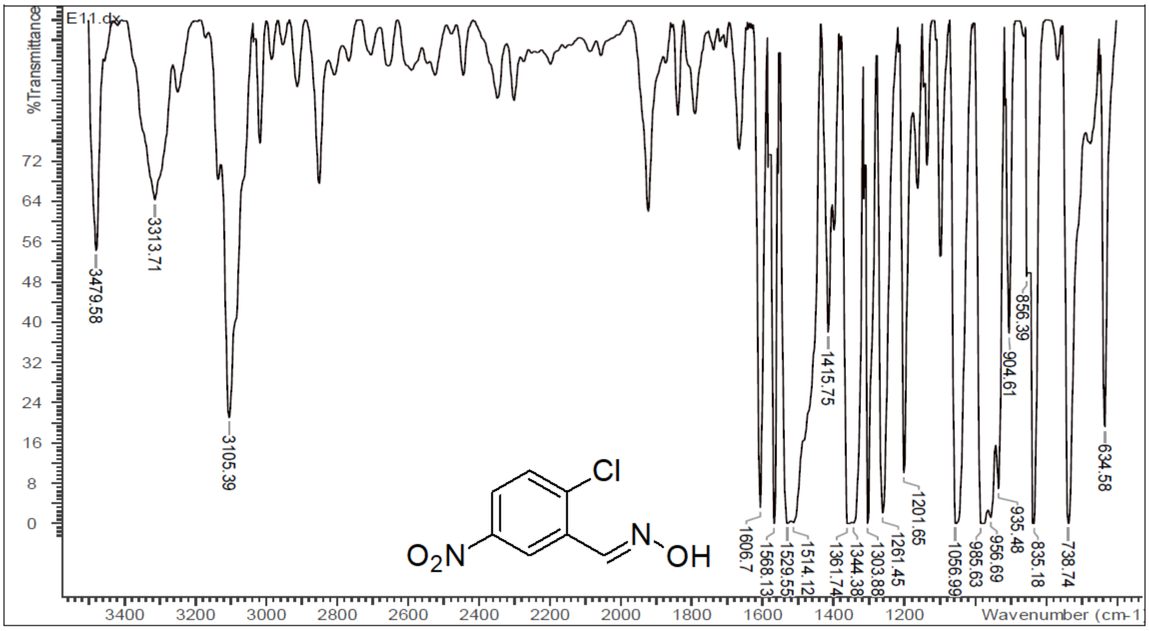

Figure S11 – IR spectrum of the compound 11.

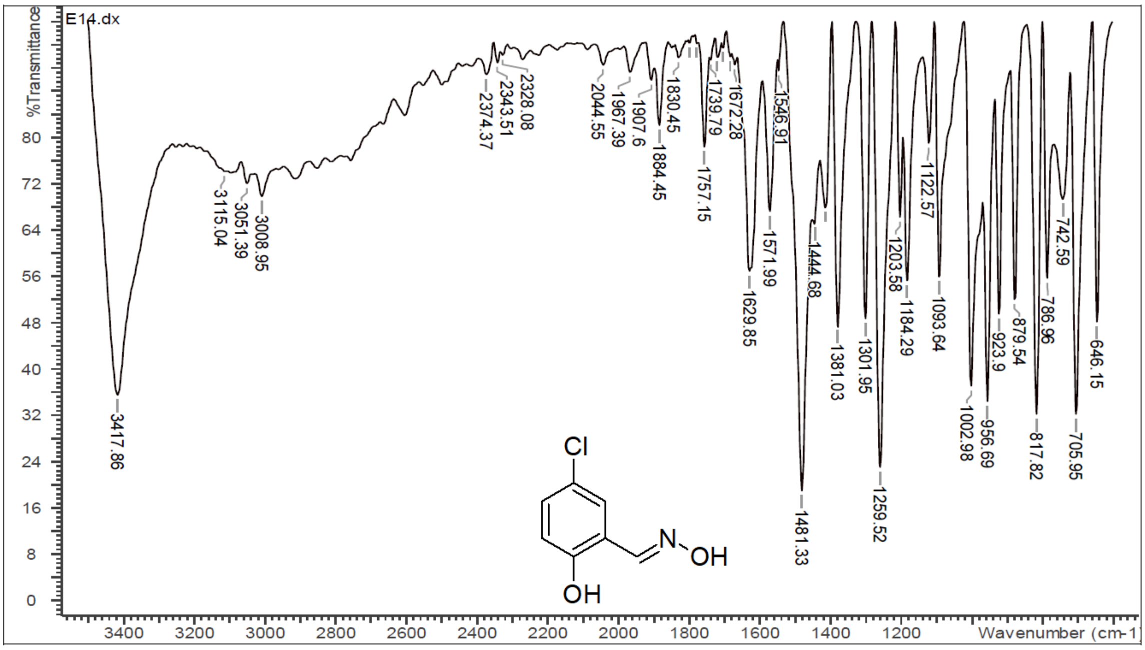

Figure S12 – IR spectrum of the compound 12.

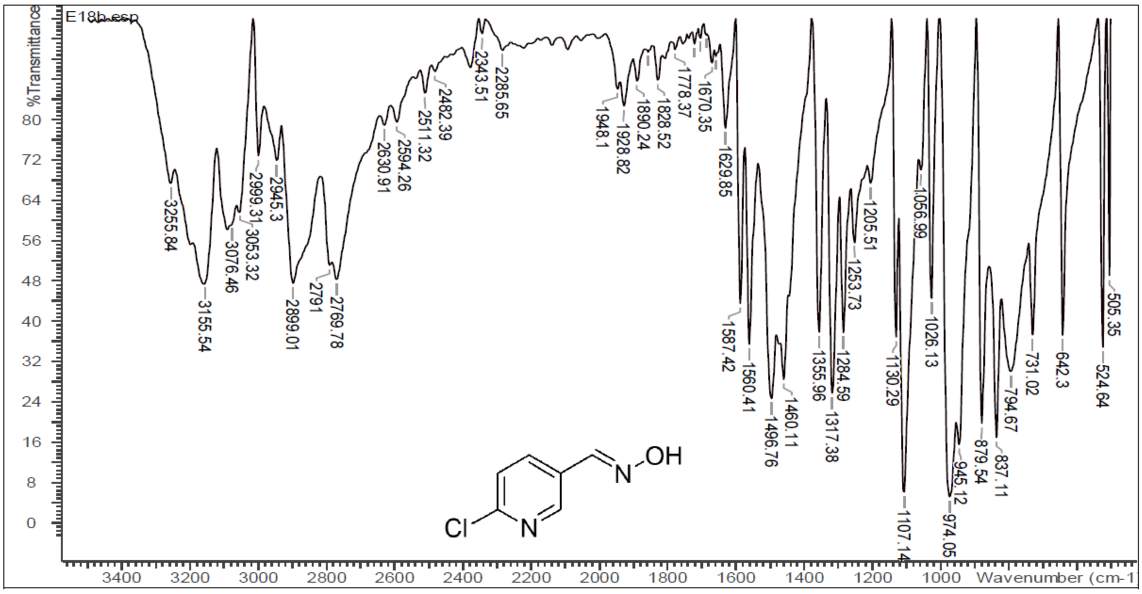

Figure S13 – IR spectrum of the compound 13.

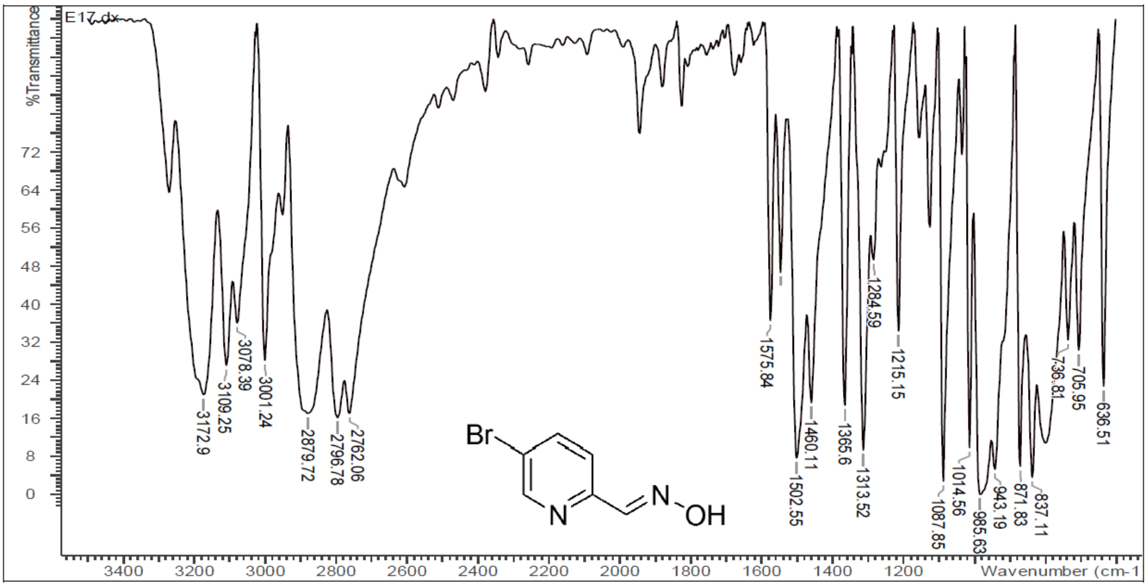

Figure S14 – IR spectrum of the compound 14.

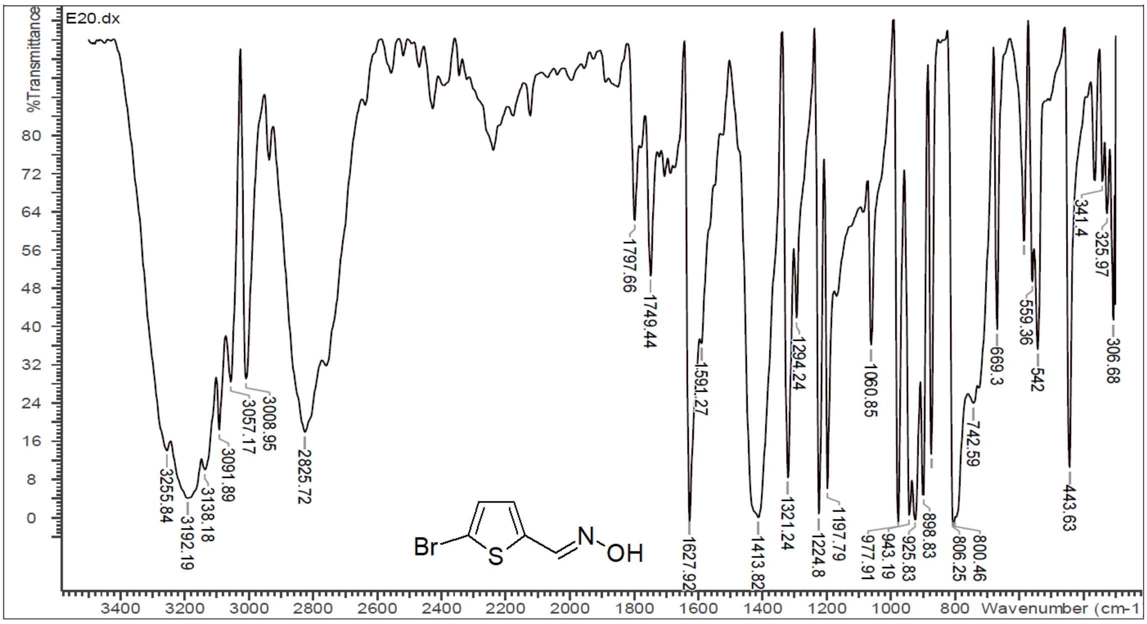

Figure S15 – IR spectrum of the compound 15.

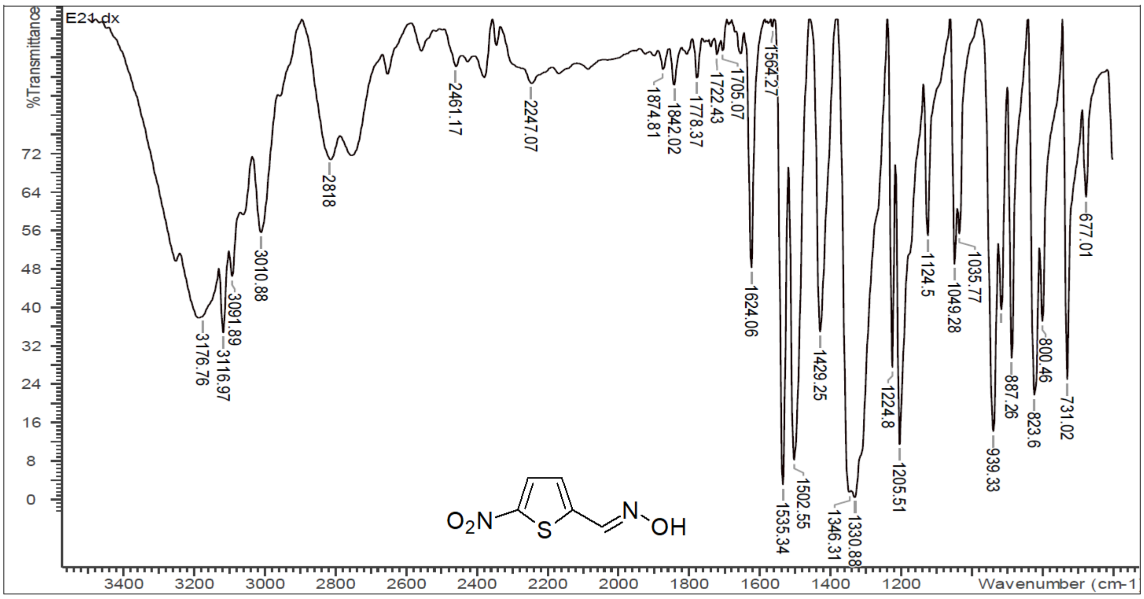

Figure S16 – IR spectrum of the compound 16.

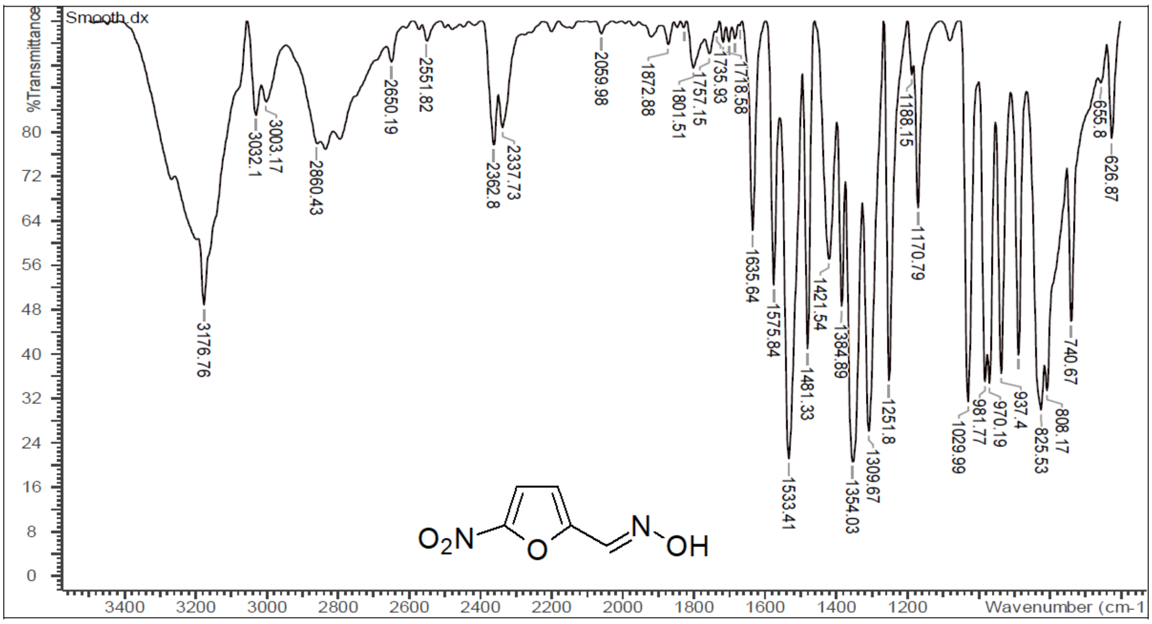

Figure S17 – IR spectrum of the compound 17.

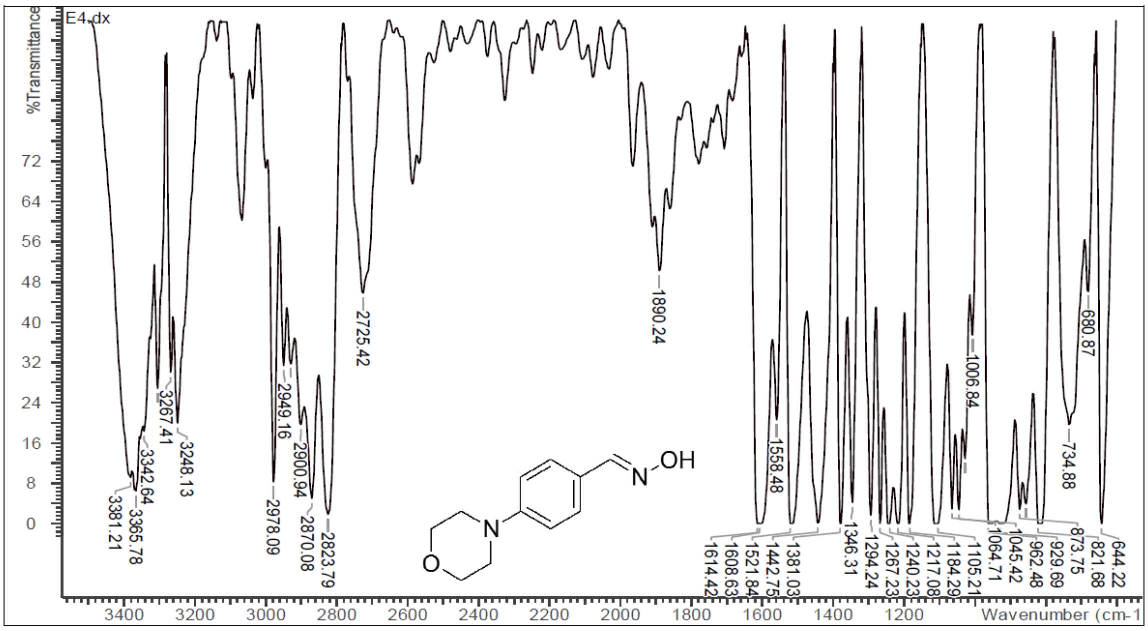

Figure S18 – IR spectrum of the compound 18.

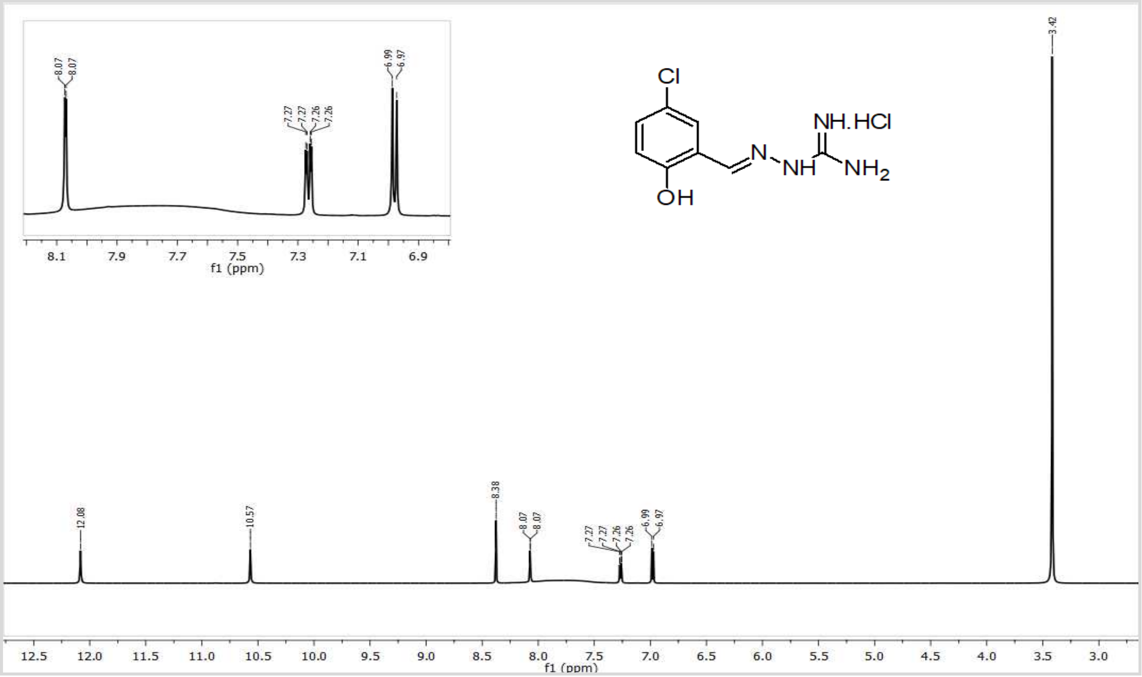

Figure S19 – ^{1}H NMR spectrum (600 MHz, DMSO-d_6) of the compound 1.

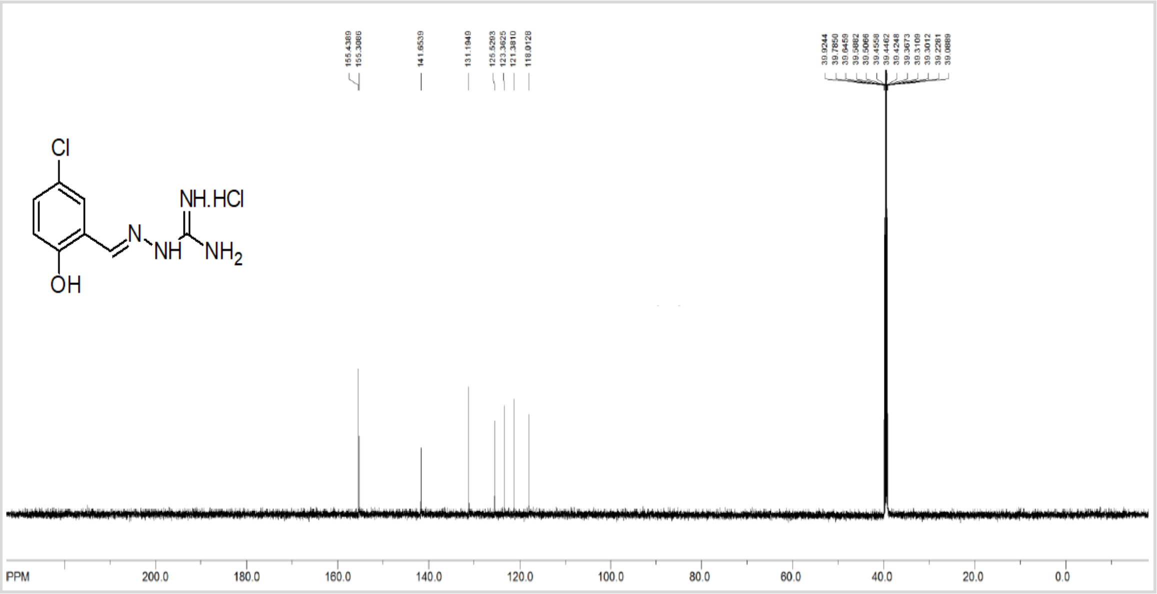

Figure S20 – ^{13}C NMR spectrum (150 MHz, DMSO-d_6) of the compound 1.

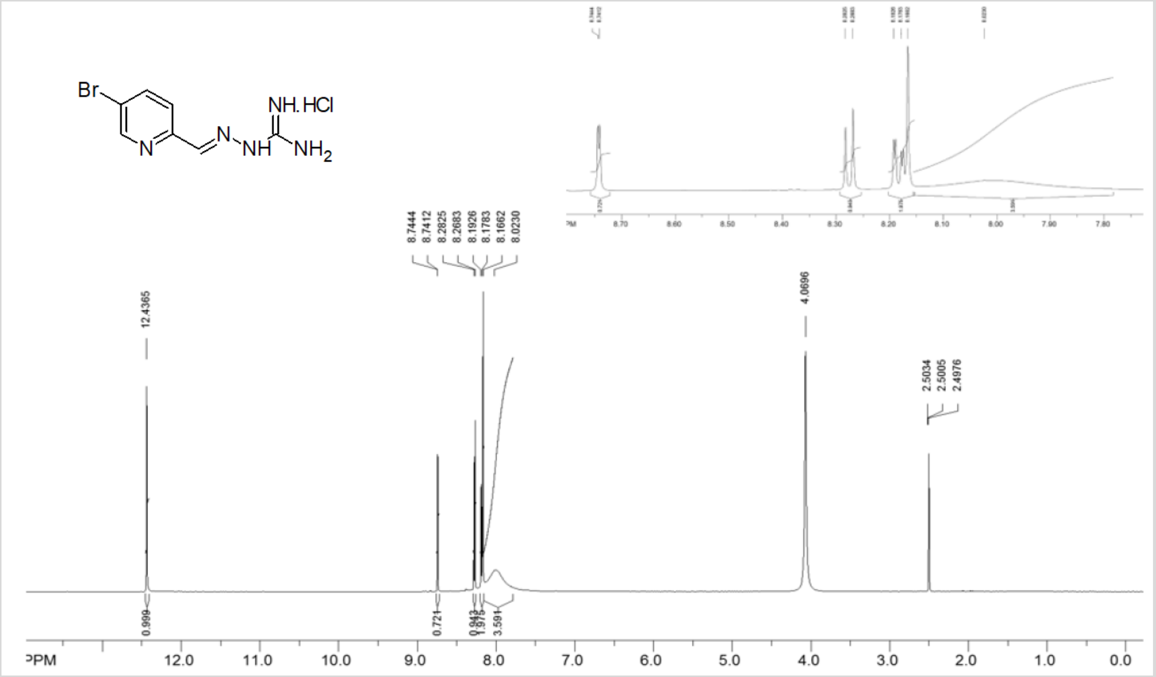

Figure S21 – ^{1}H NMR spectrum (600 MHz, DMSO-d_6) of the compound 2.

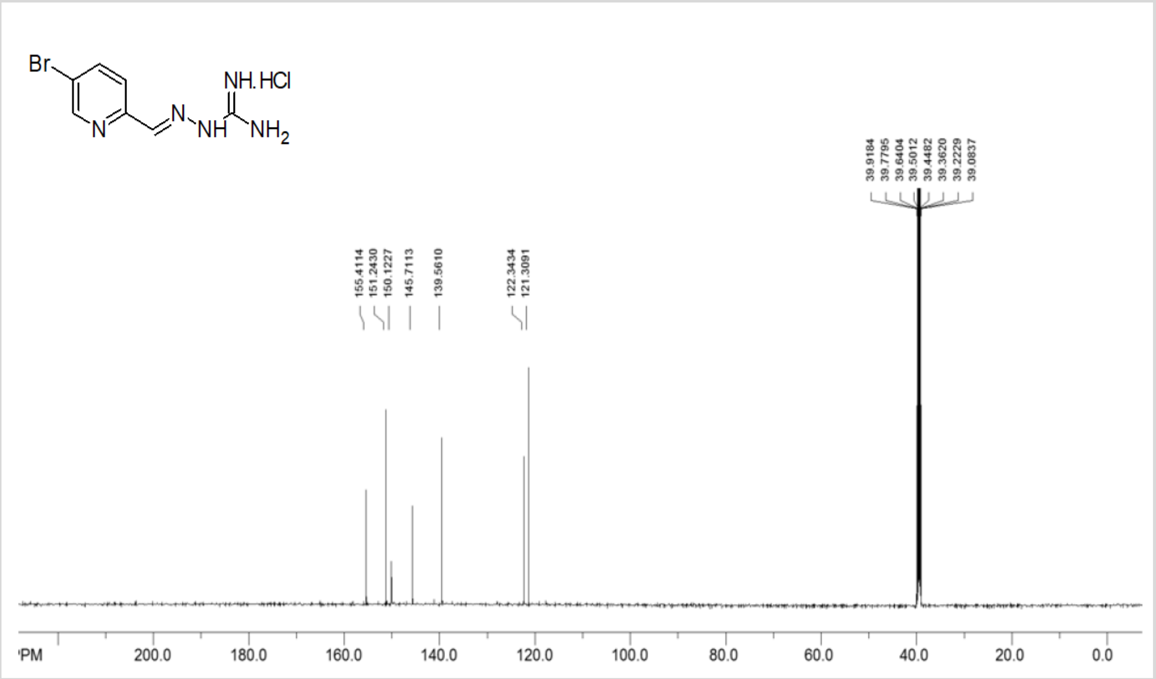

Figure S22 – ^{13}C NMR spectrum (150 MHz, DMSO-d_6) of the compound 2.

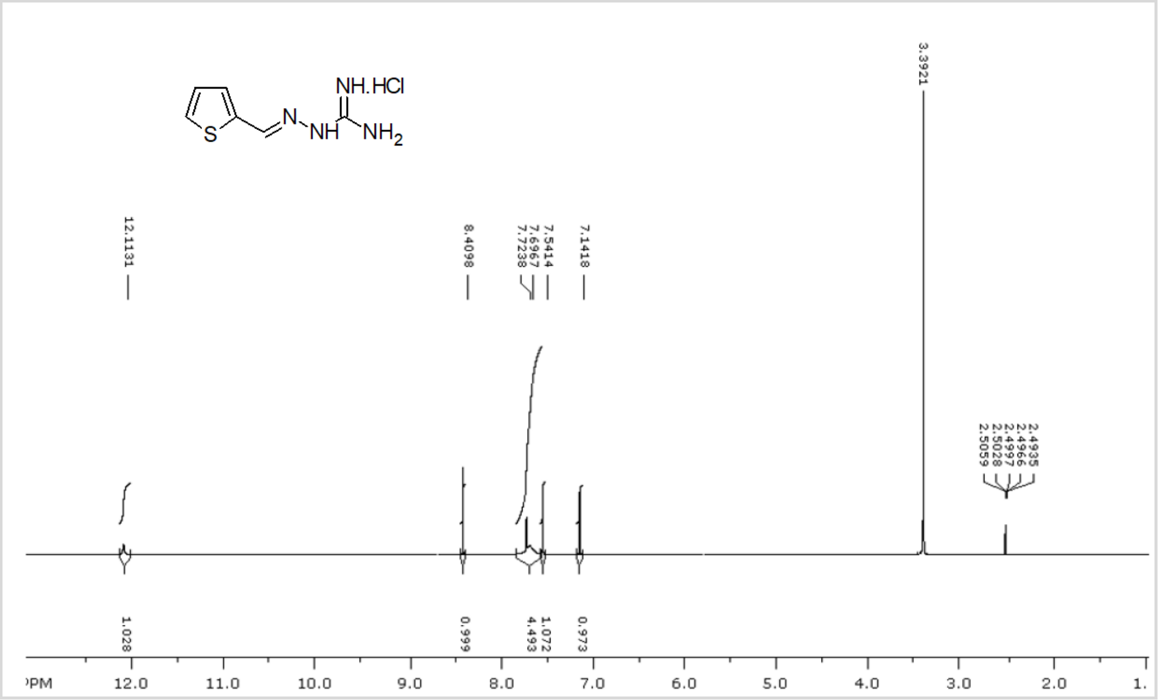

Figure S23 – ^{1}H NMR spectrum (600 MHz, DMSO-*d*$_6$) of the compound 3.

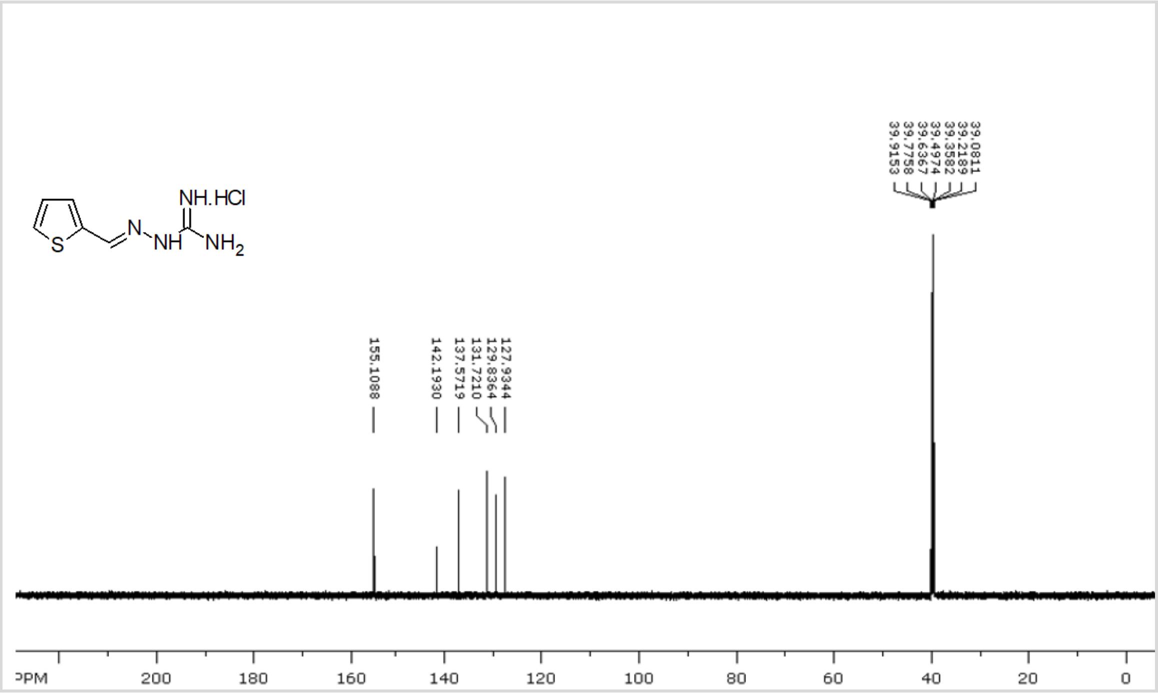

Figure S24 – ^{13}C NMR spectrum (150 MHz, DMSO-*d*$_6$) of the compound 3.

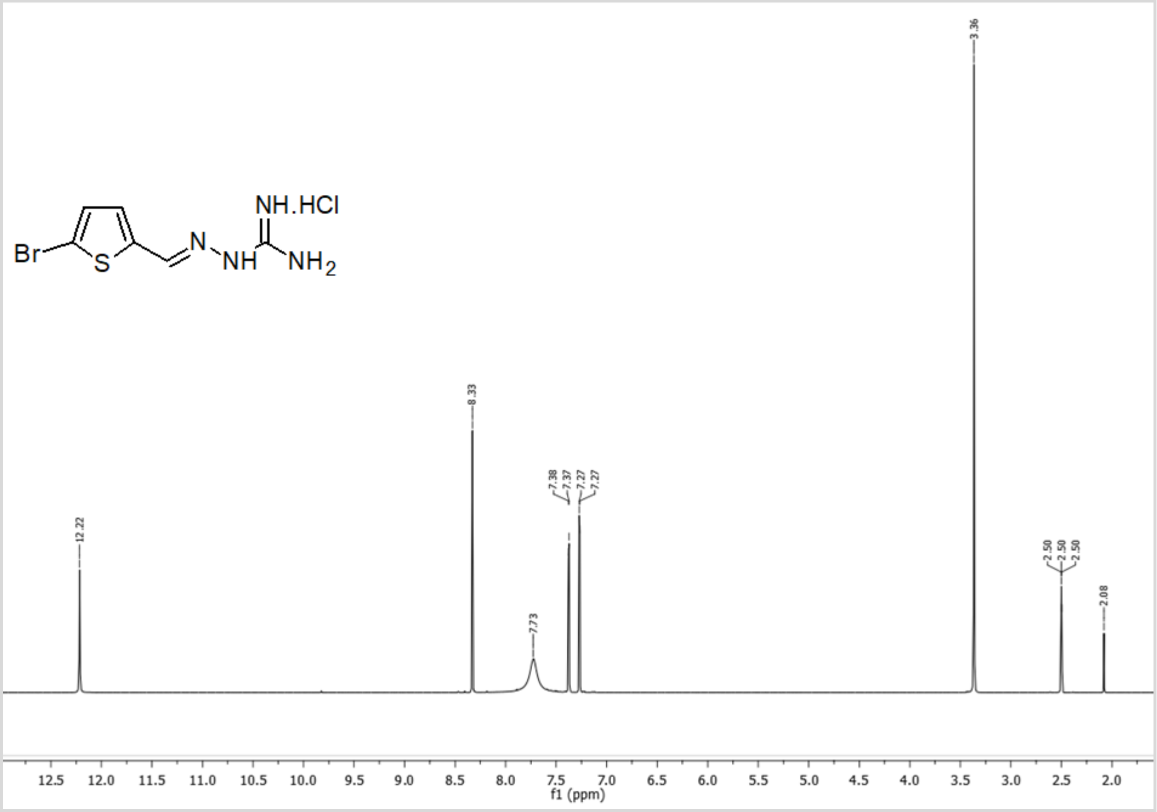

Figure S25 – ^{1}H NMR spectrum (600 MHz, DMSO-d_6) of the compound 4.

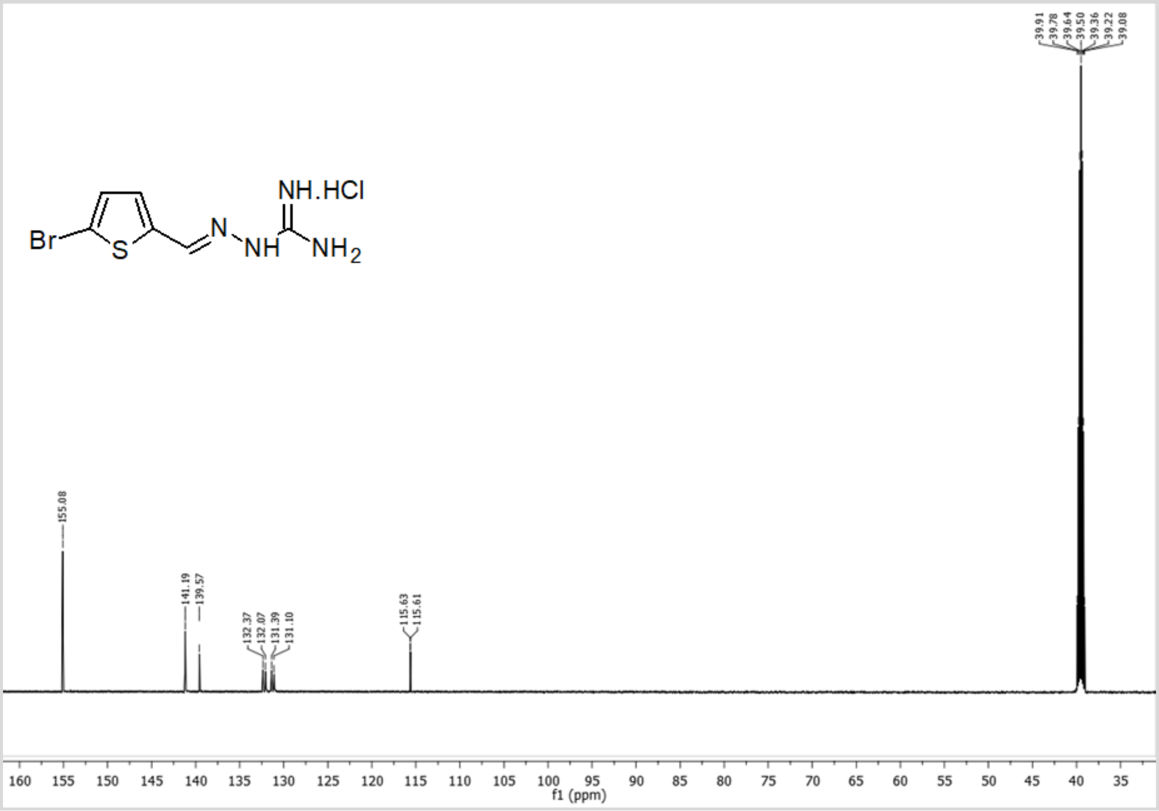

Figure S26 – ^{13}C-coupled NMR spectrum (150 MHz, DMSO-d_6) of the compound 4.

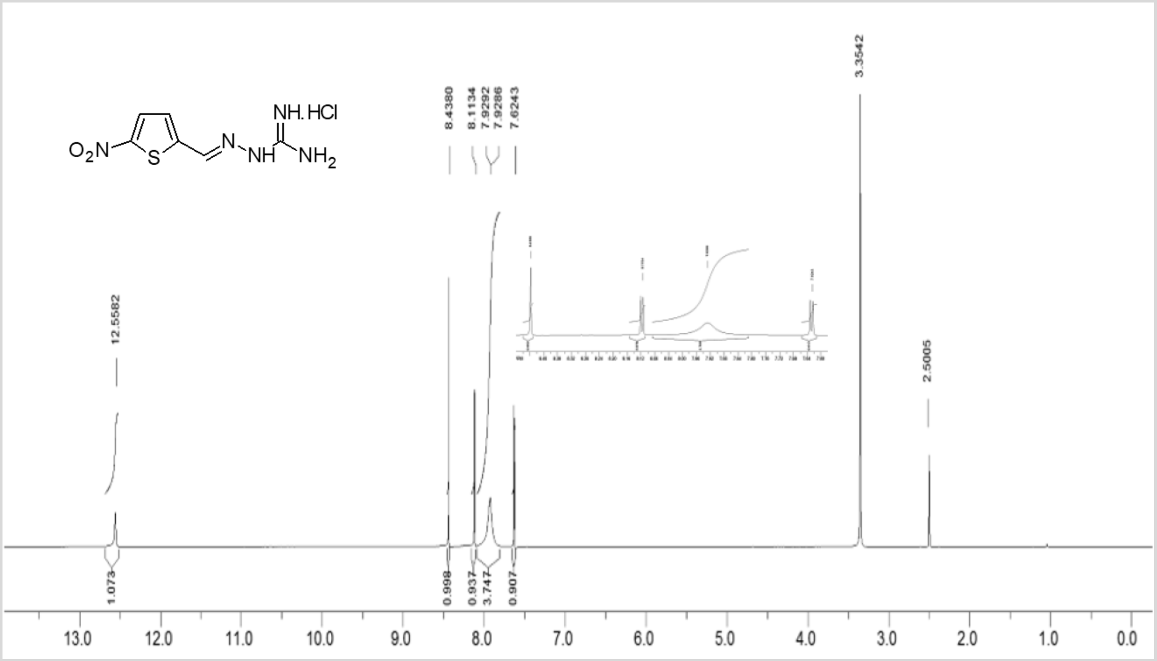

Figure S27 – ^{1}H NMR spectrum (600 MHz, DMSO-d_6) of the compound 5.

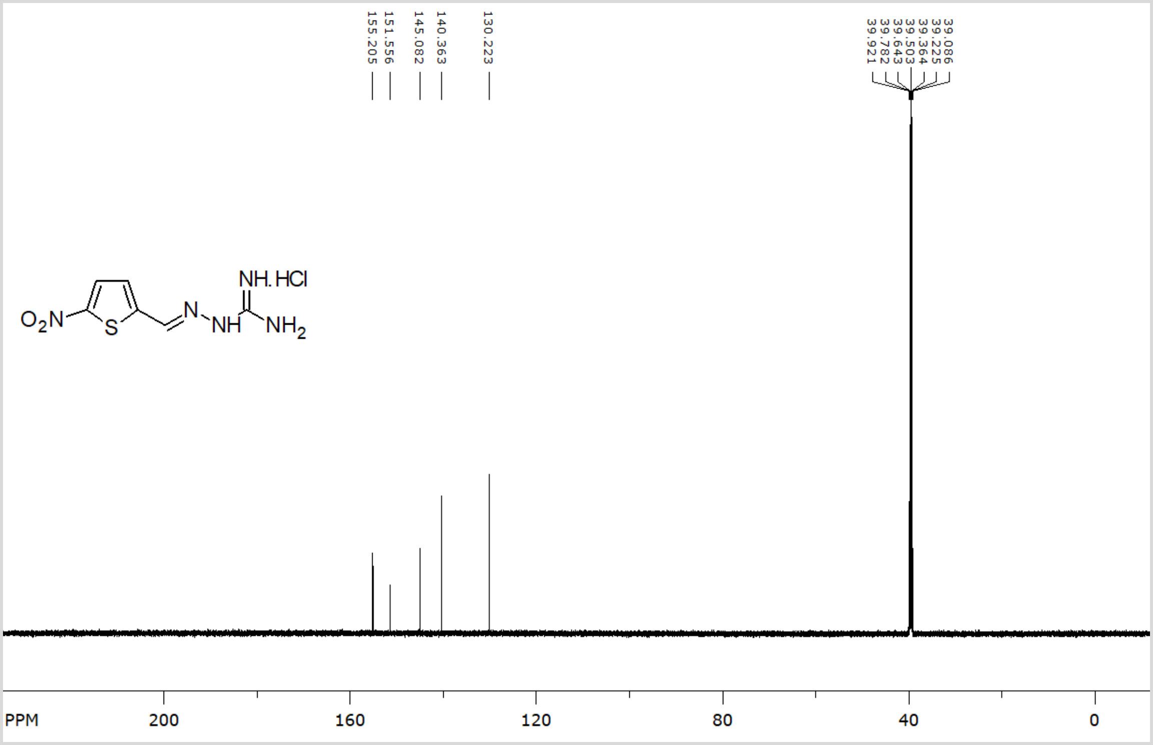

Figure S28 – ^{13}C NMR spectrum (150 MHz, DMSO-d_6) of the compound 5.

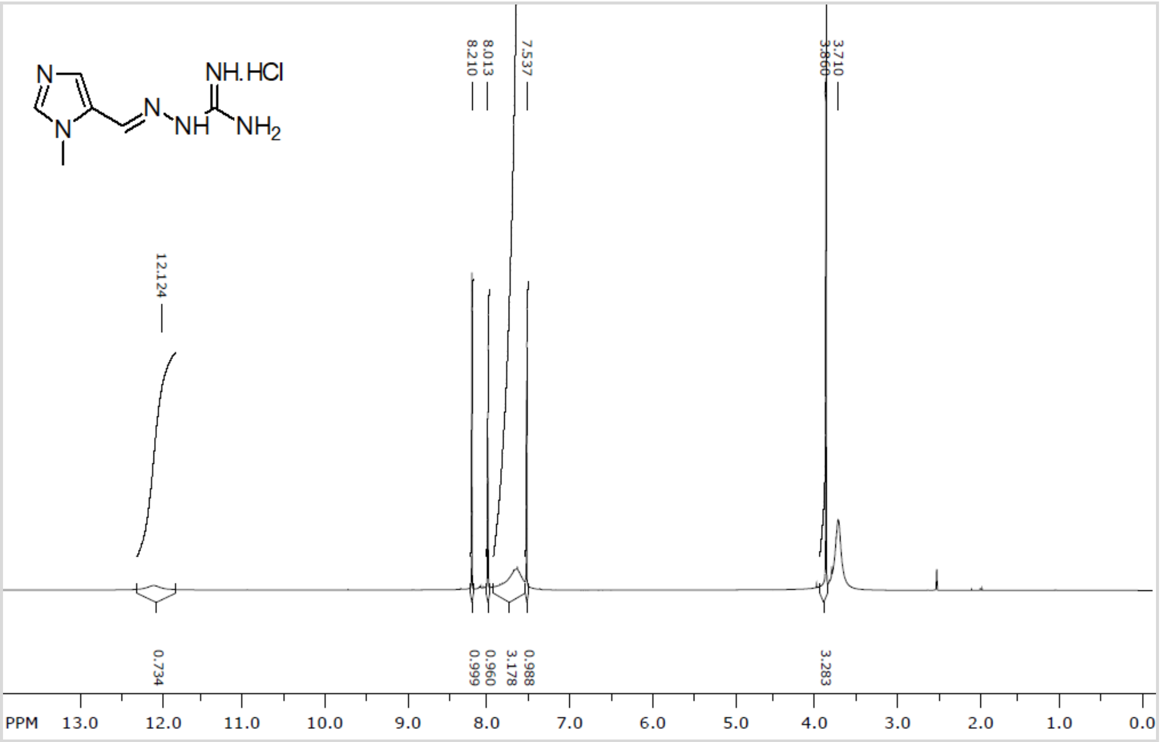

Figure S29 – ^{1}H NMR spectrum (600 MHz, DMSO-d_6) of the compound 6.

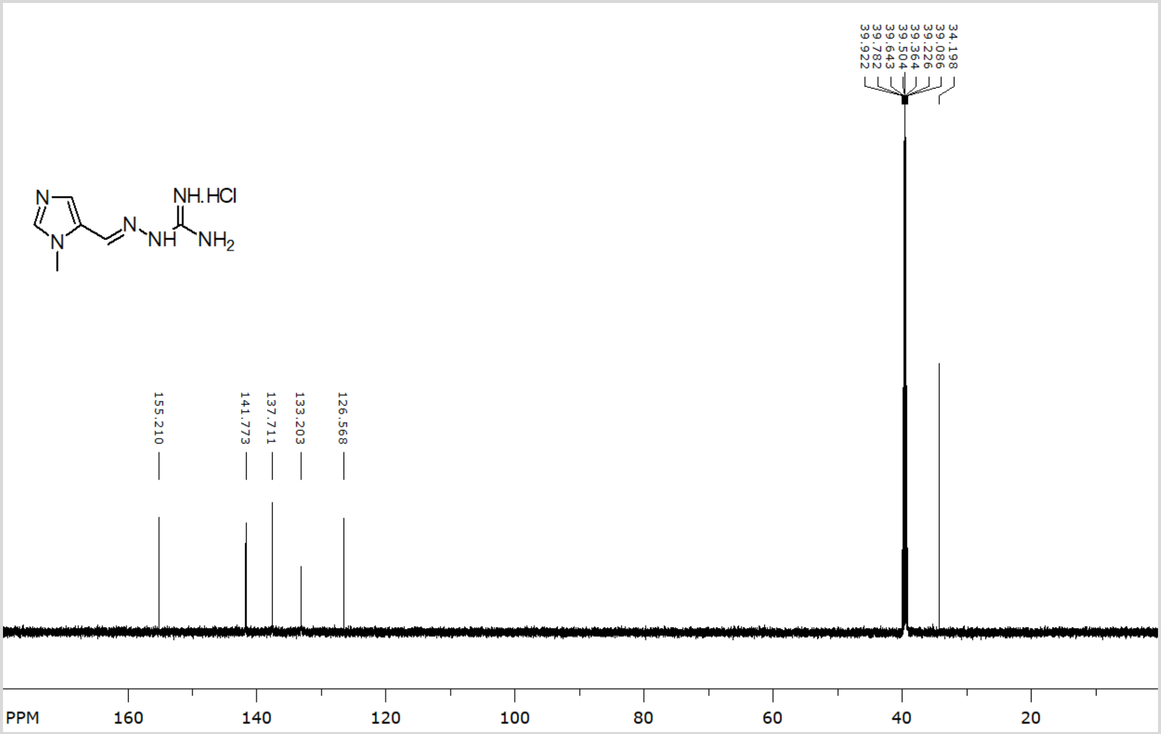

Figure S30 – ^{13}C NMR spectrum (150 MHz, DMSO-d_6) of the compound 6.

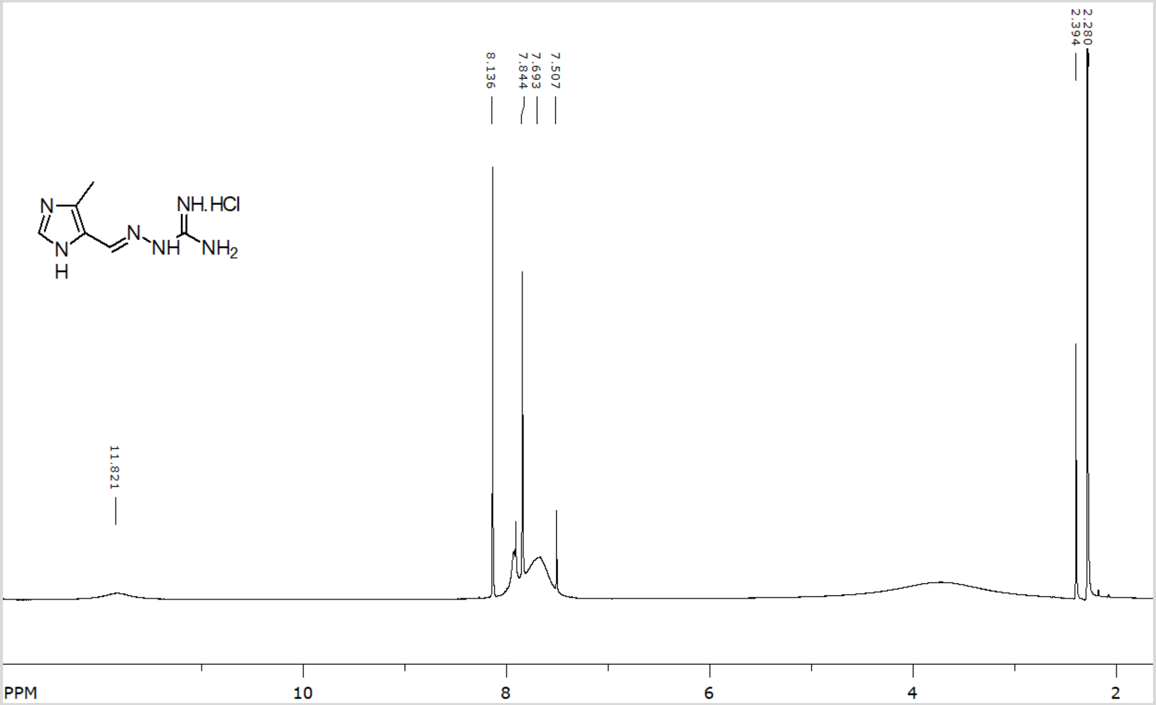

Figure S31 – ^{1}H NMR spectrum (600 MHz, DMSO-*d*$_6$) of the compound 7.

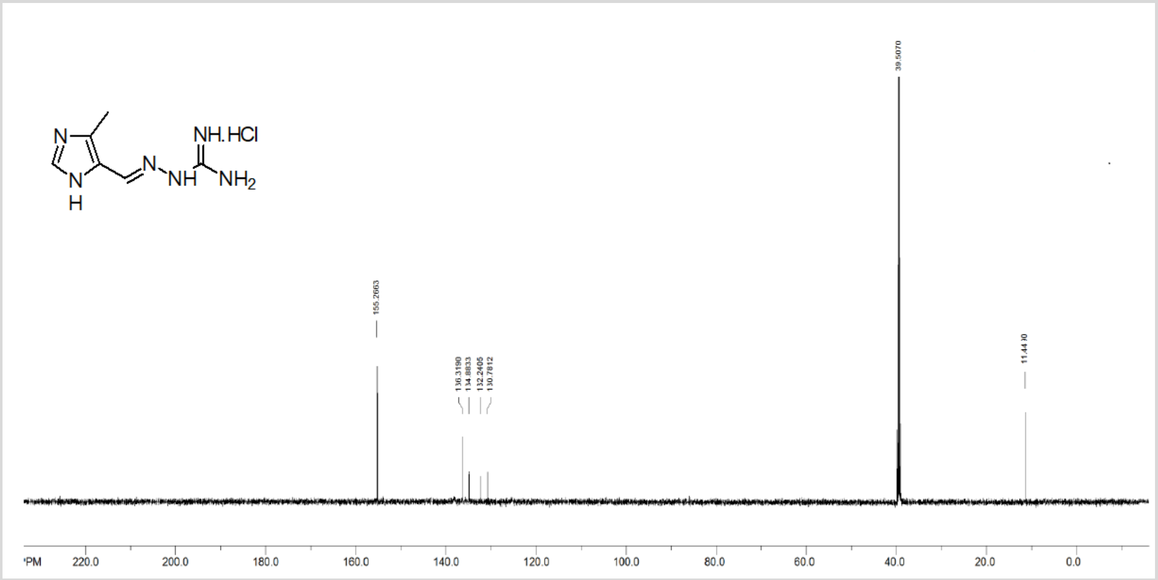

Figure S32 – ^{13}C NMR spectrum (150 MHz, DMSO-*d*$_6$) of the compound 7.

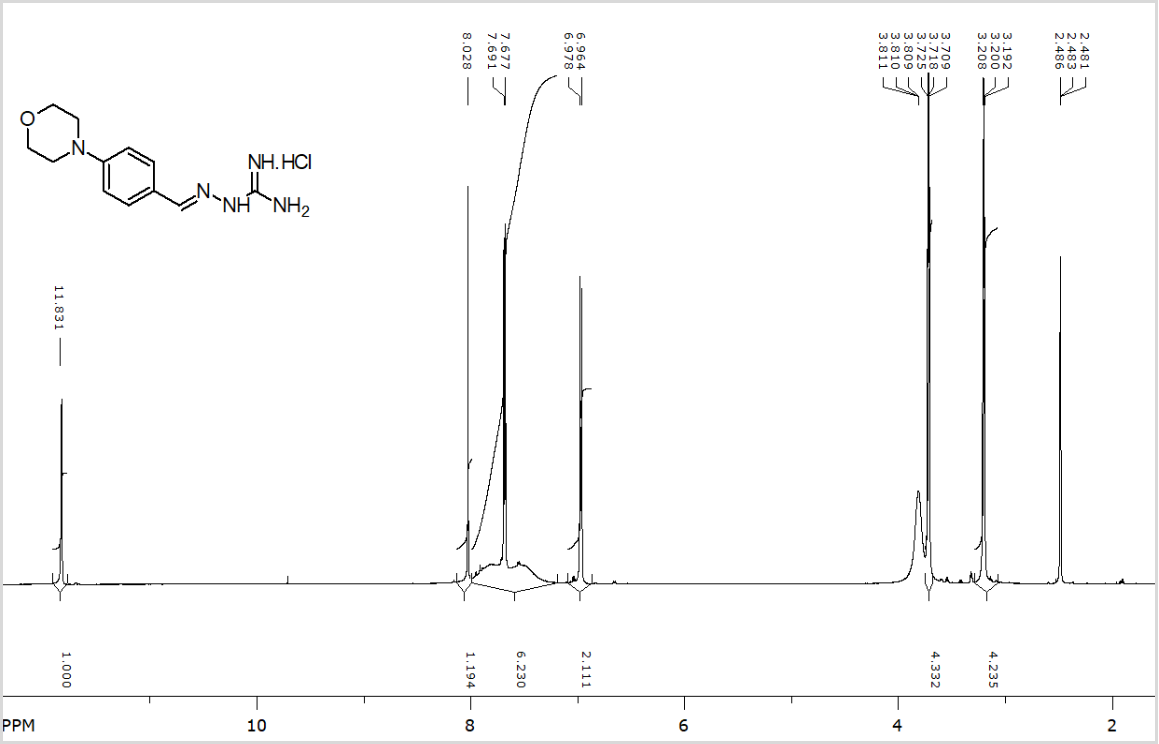

Figure S33 – ^{1}H NMR spectrum (600 MHz, DMSO-*d$_6$*) of the compound 8.

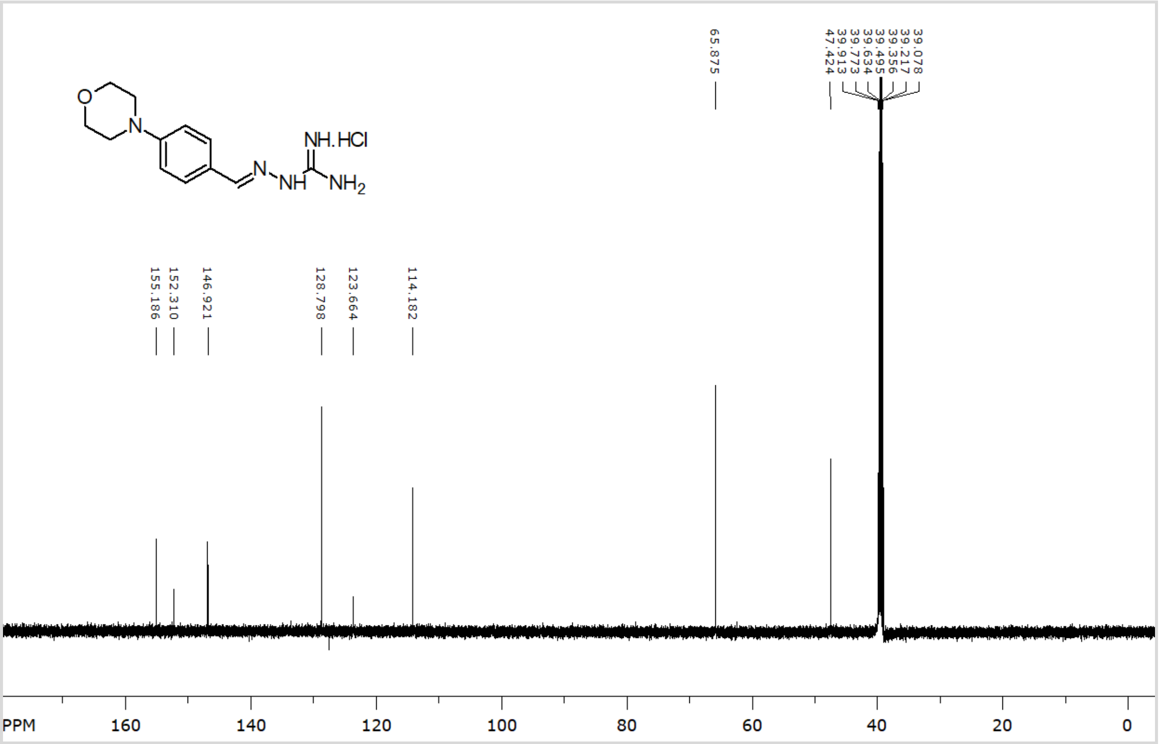

Figure S34 – ^{13}C NMR spectrum (150 MHz, DMSO-*d$_6$*) of the compound 8.

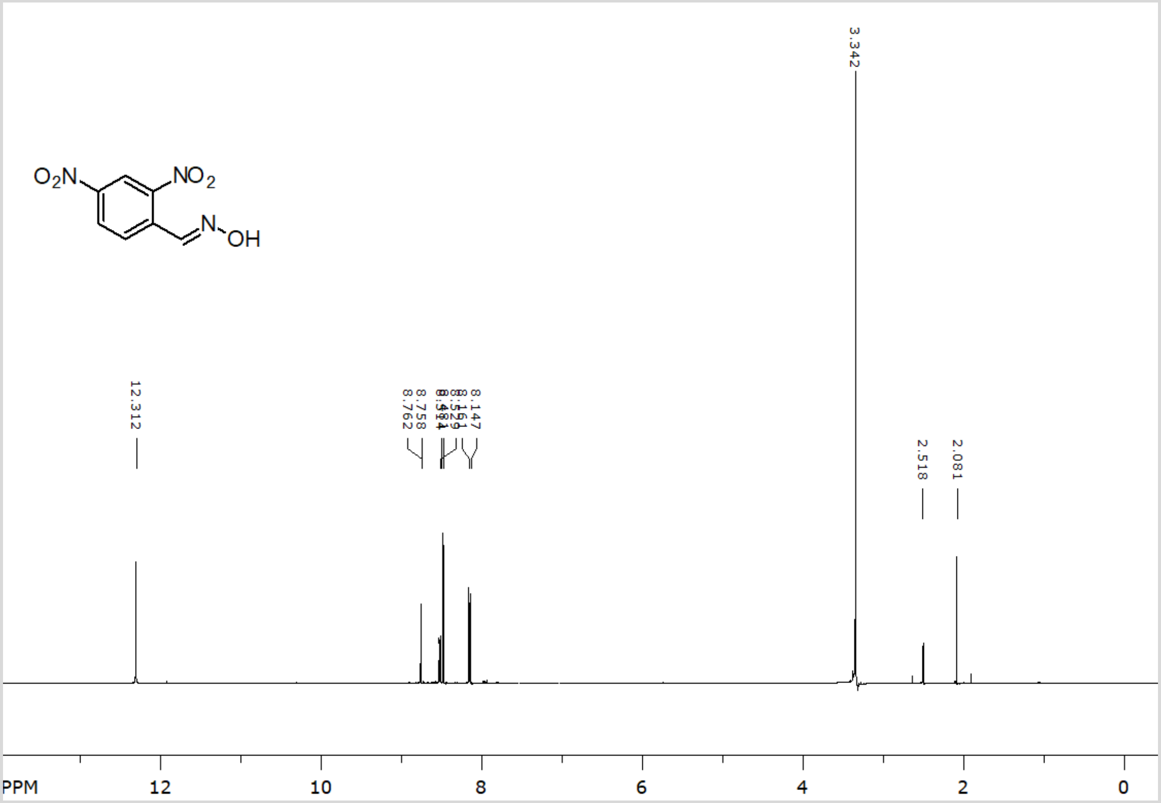

Figure S35 – ^{1}H NMR spectrum (600 MHz, DMSO-d_6) of the compound 9.

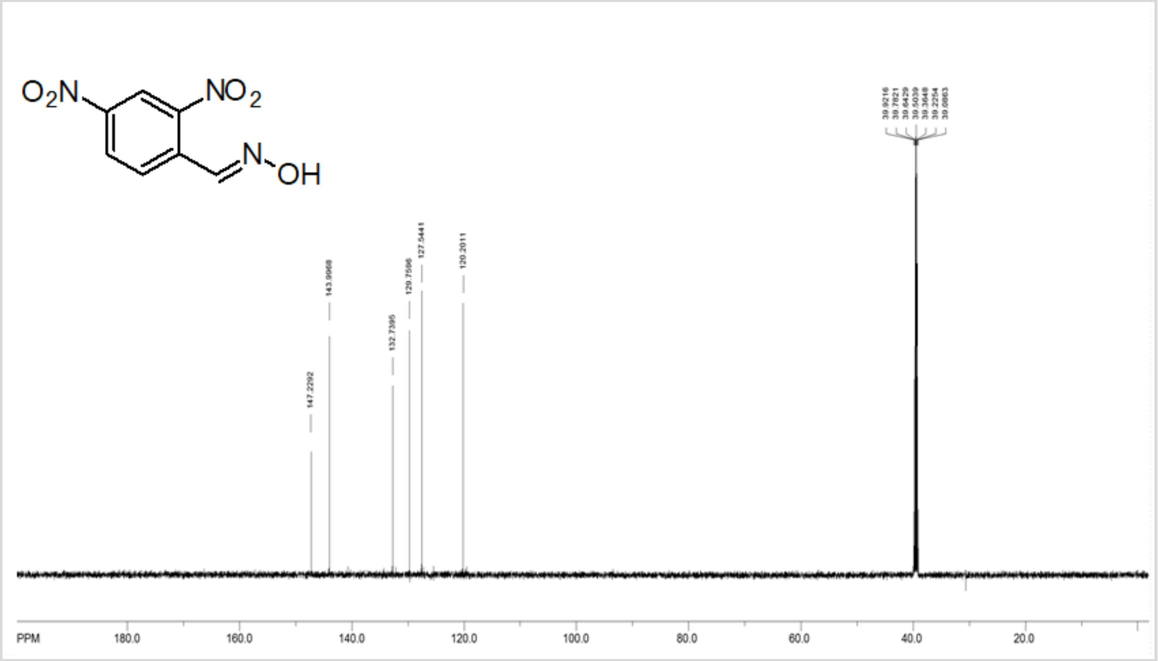

Figure S36 – ^{13}C NMR spectrum (150 MHz, DMSO-d_6) of the compound 9.

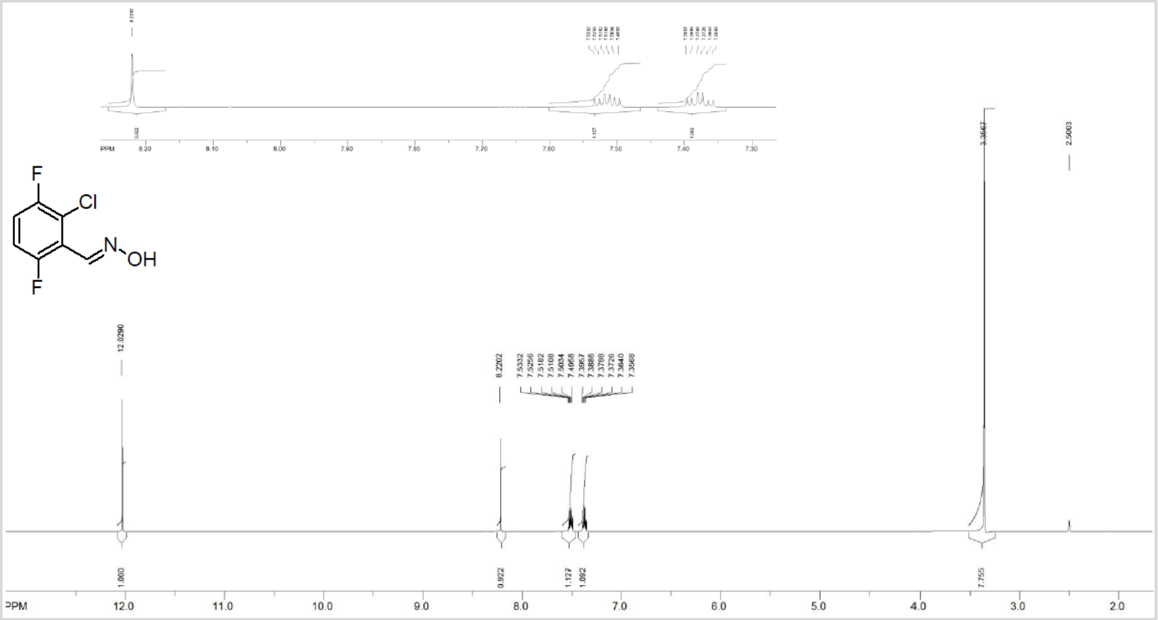

Figure S37 – ^{1}H NMR spectrum (600 MHz, DMSO-d_6) of the compound 10.

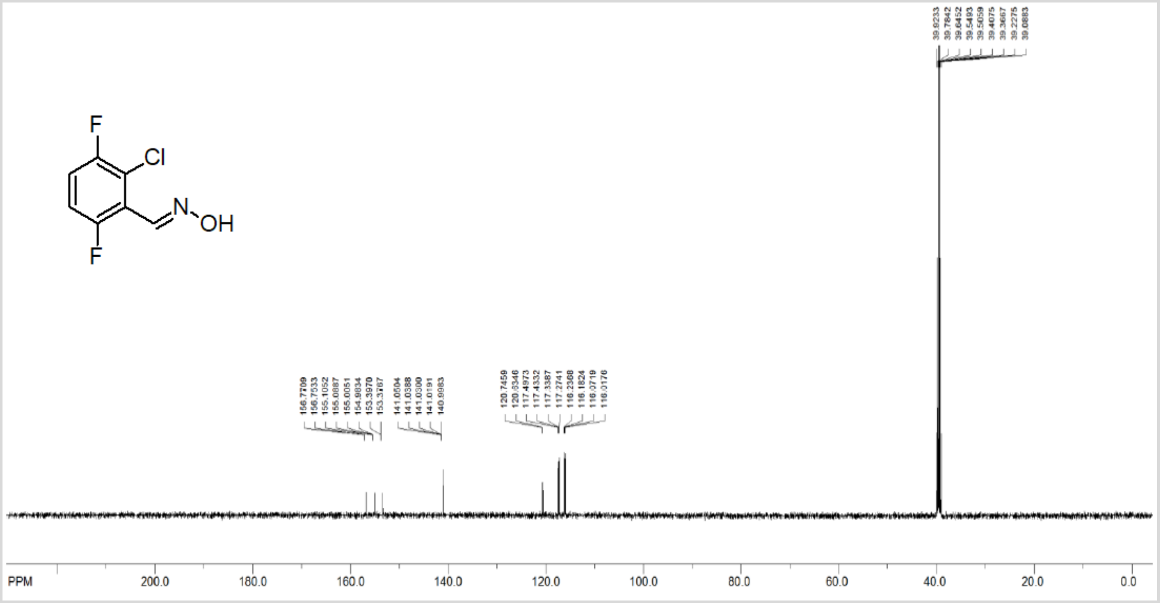

Figure S38 – ^{13}C (^{19}F-coupled) NMR spectrum (150 MHz, DMSO-d_6) of the compound 10.

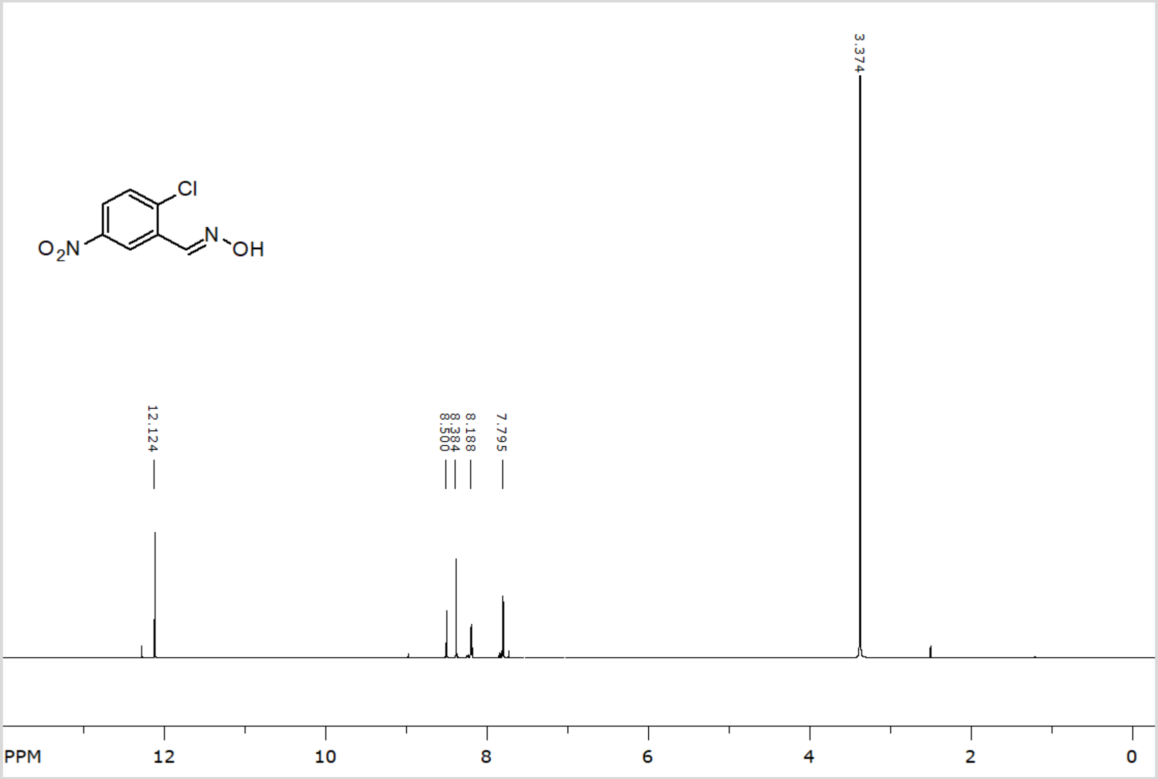

Figure S39 – ^{1}H NMR spectrum (600 MHz, DMSO-d_6) of the compound 11.

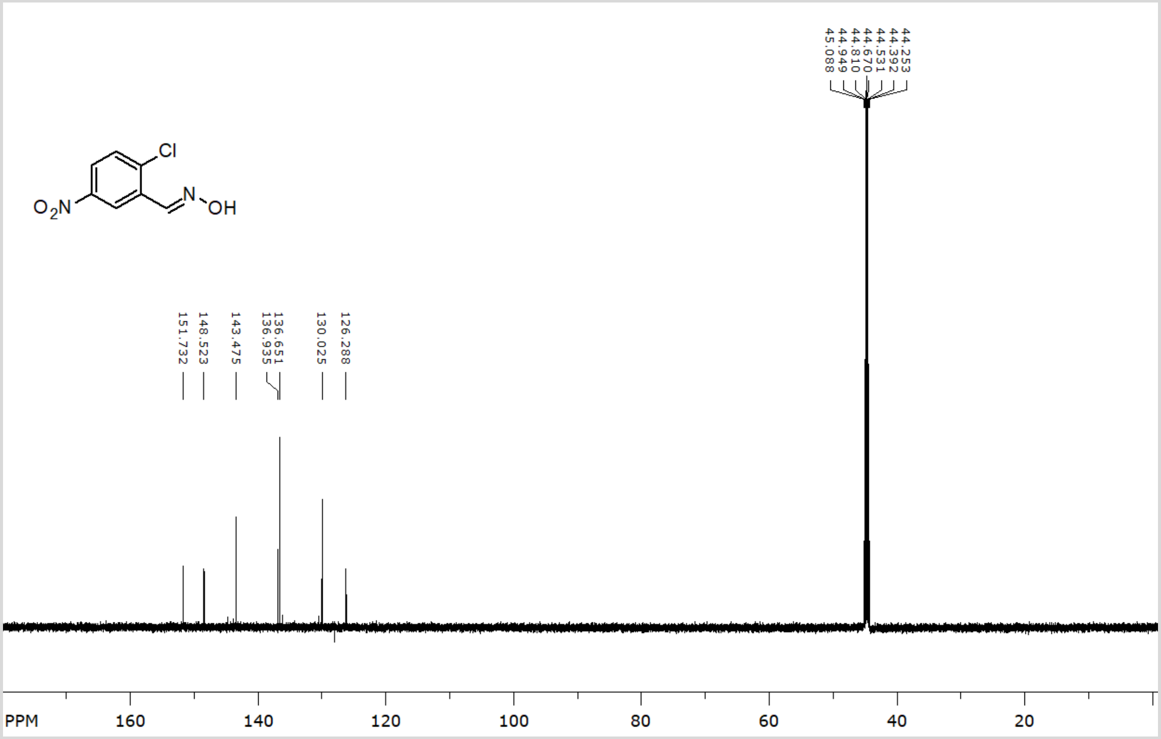

Figure S40 – ^{13}C NMR spectrum (150 MHz, DMSO-d_6) of the compound 11.

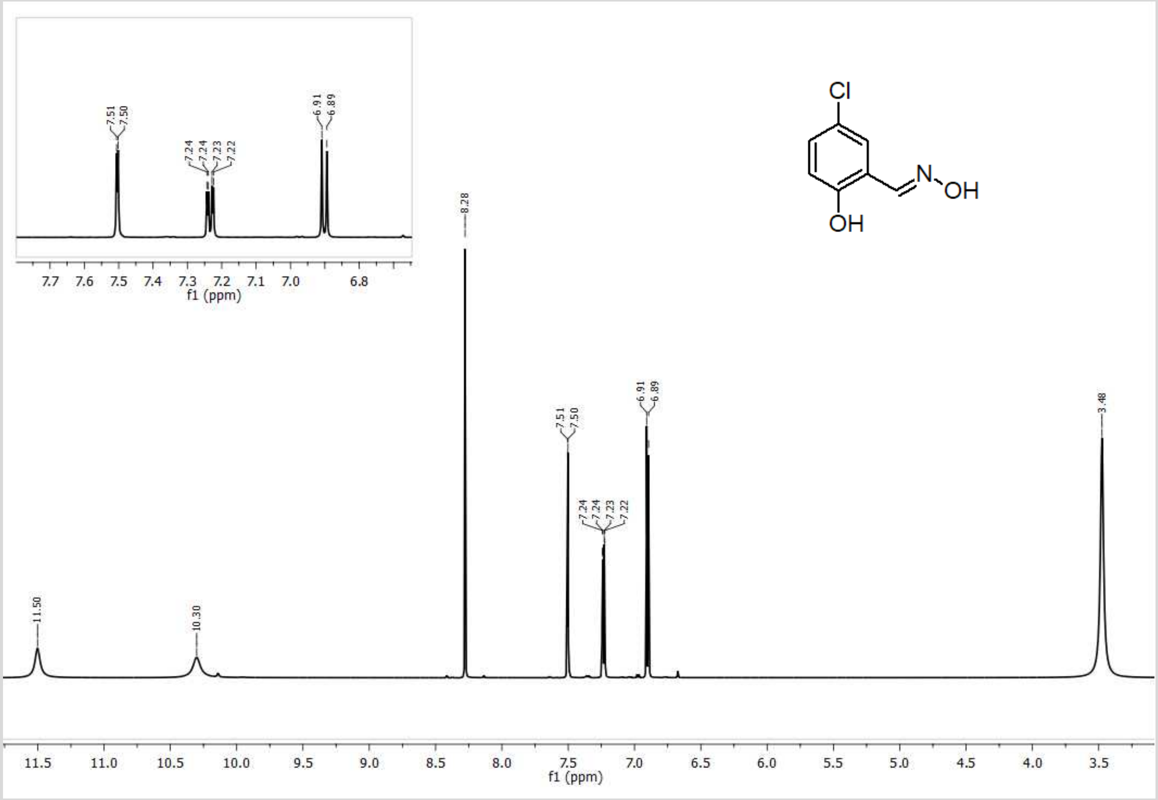

Figure S41 – ^{1}H NMR spectrum (600 MHz, DMSO-d_6) of the compound 12.

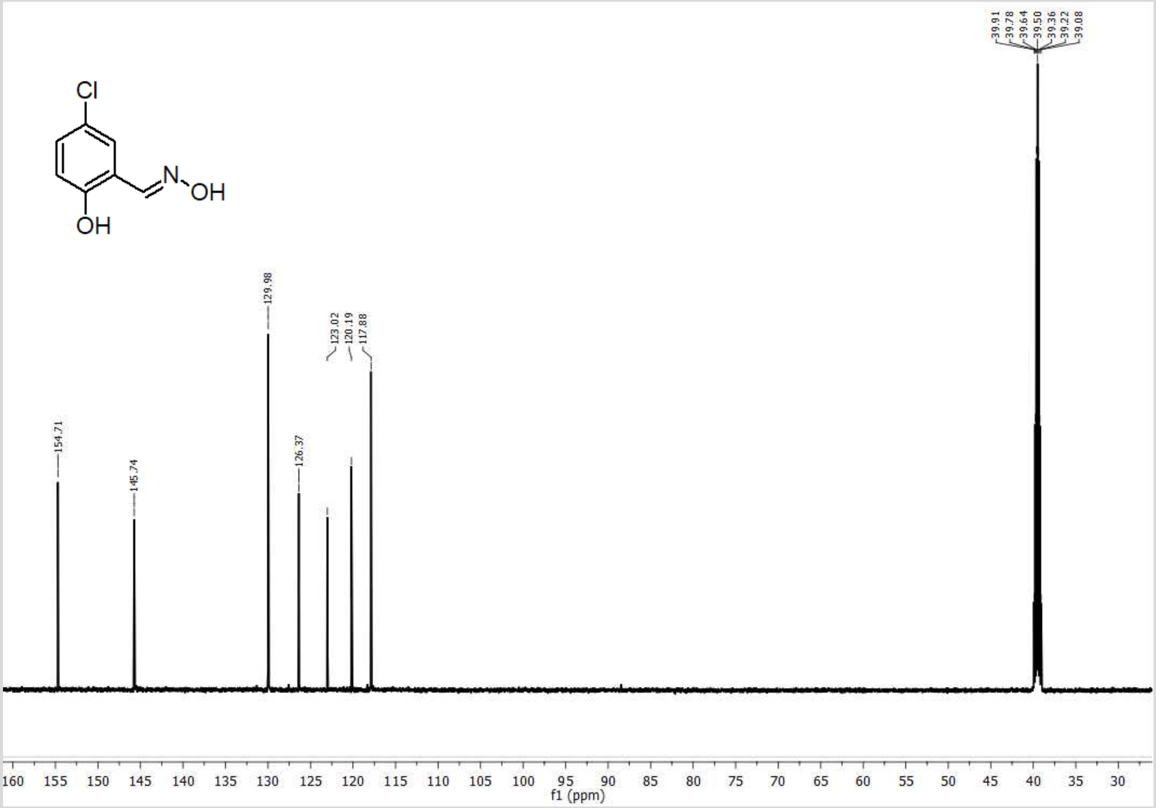

Figure S42 – ^{13}C NMR spectrum (150 MHz, DMSO-d_6) of the compound 12.

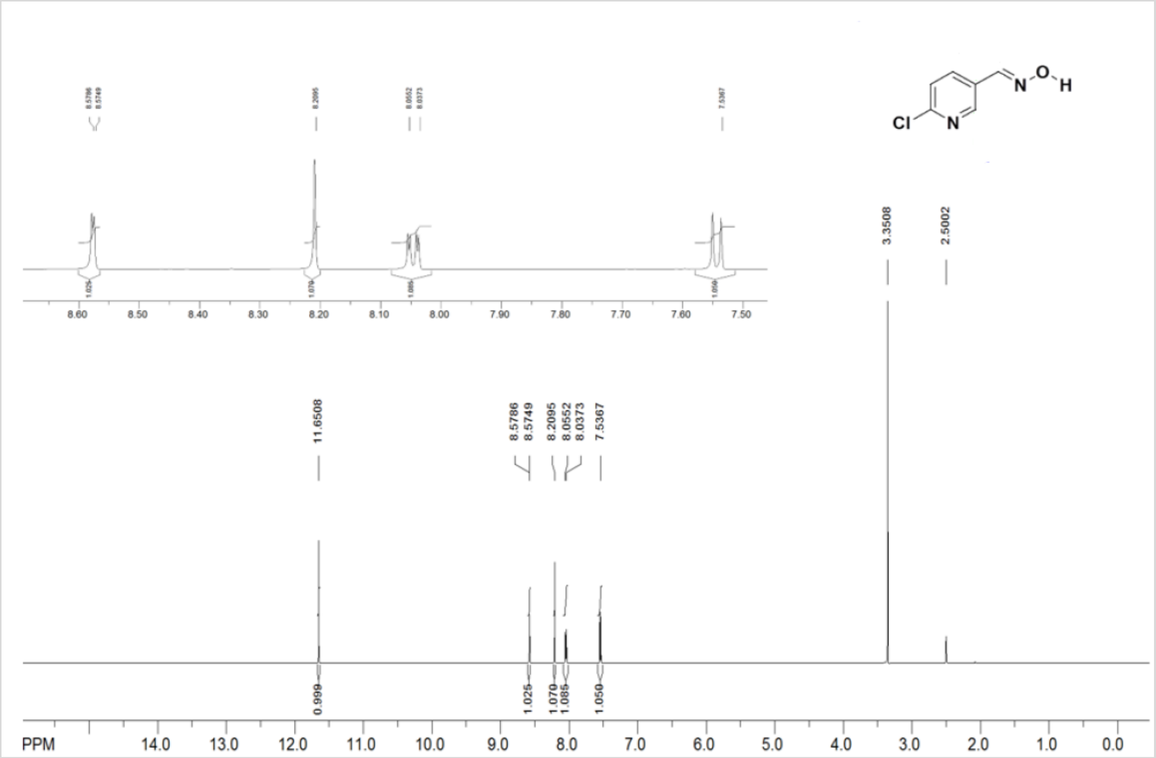

Figure S43 – ^{1}H NMR spectrum (600 MHz, DMSO-*d*$_6$) of the compound 13.

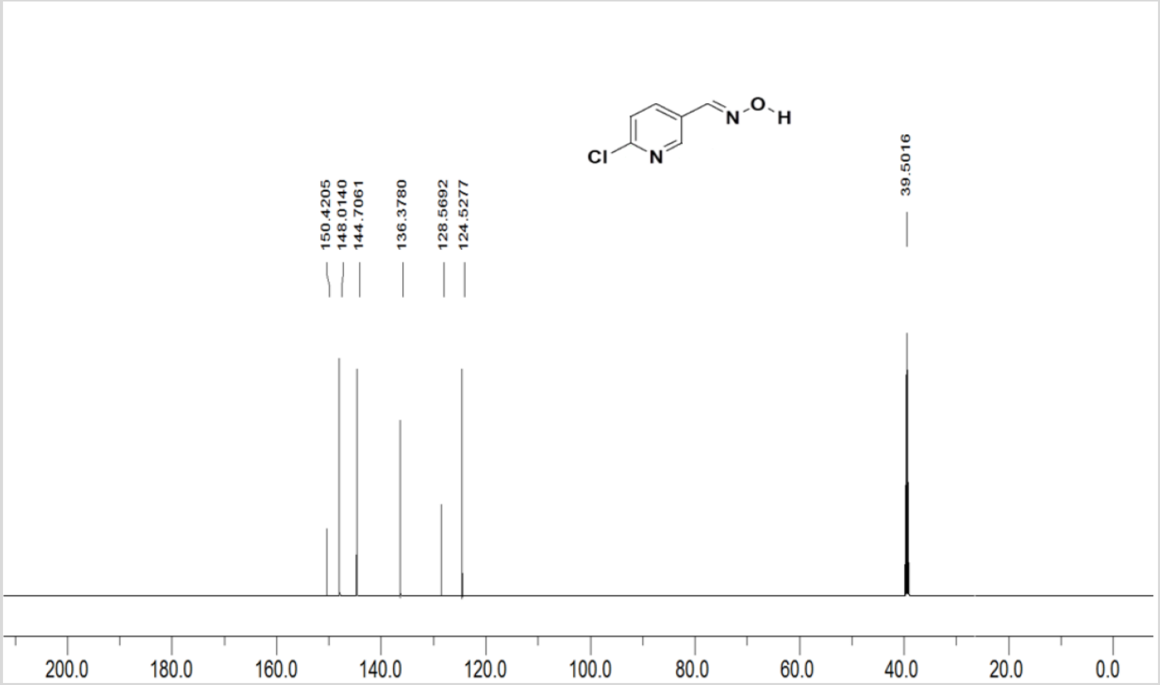

Figure S44 – ^{13}C NMR spectrum (150 MHz, DMSO-*d*$_6$) of the compound 13.

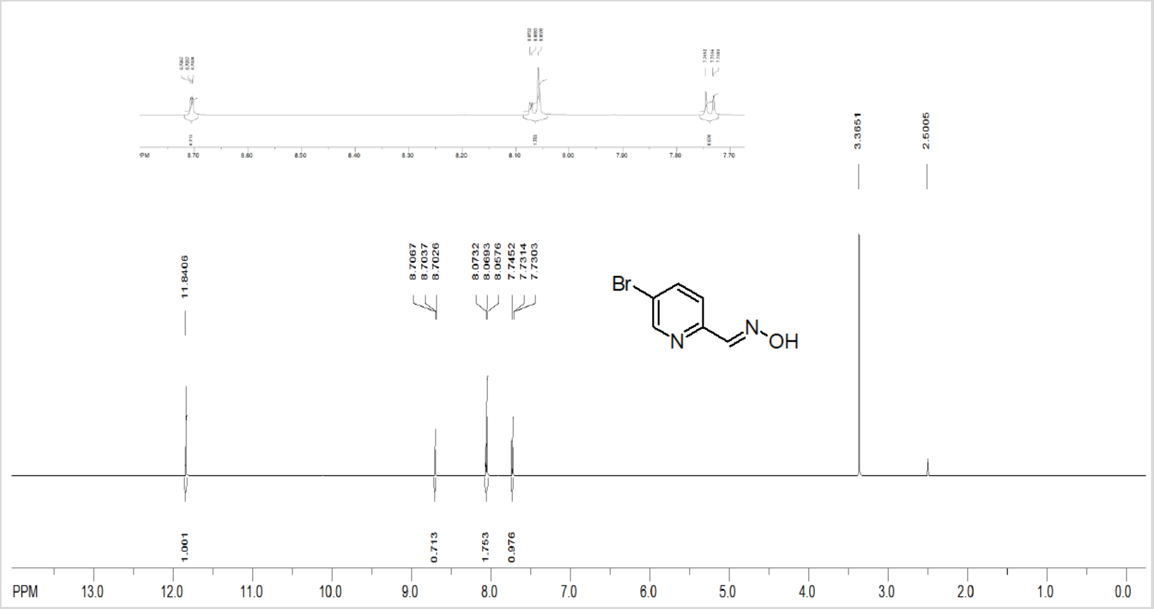

Figure S45 – ^{1}H NMR spectrum (600 MHz, DMSO-d_6) of the compound 14.

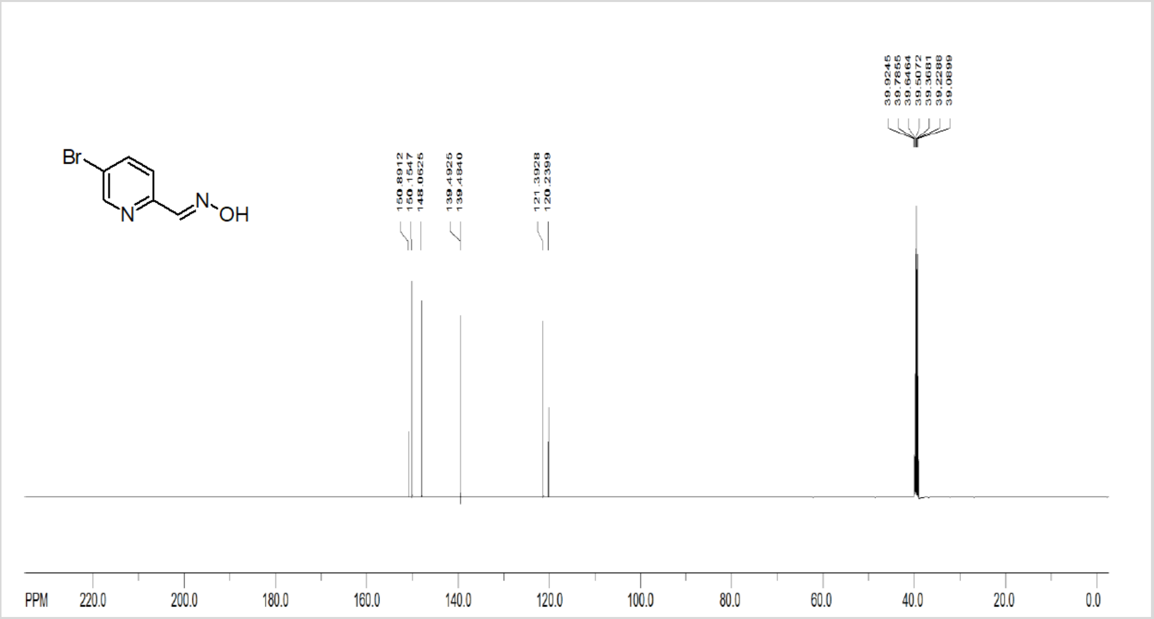

Figure S46 – ^{13}C NMR spectrum (150 MHz, DMSO-d_6) of the compound 14.

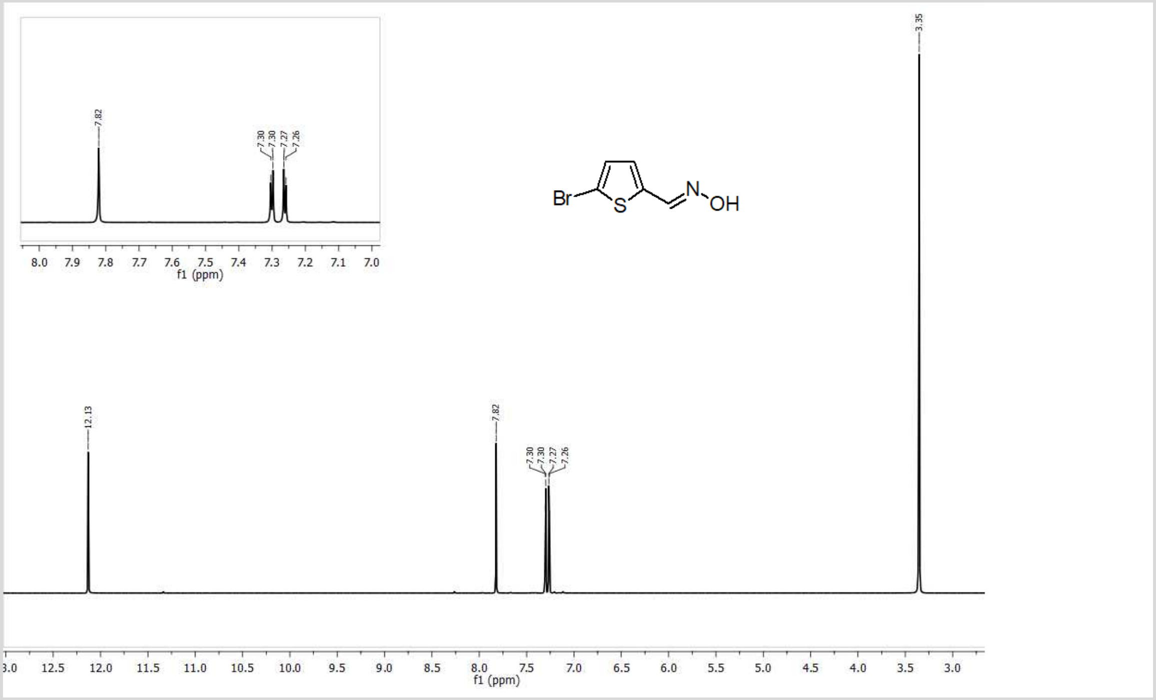

Figure S47 – ^{1}H NMR spectrum (600 MHz, DMSO-d_6) of the compound 15.

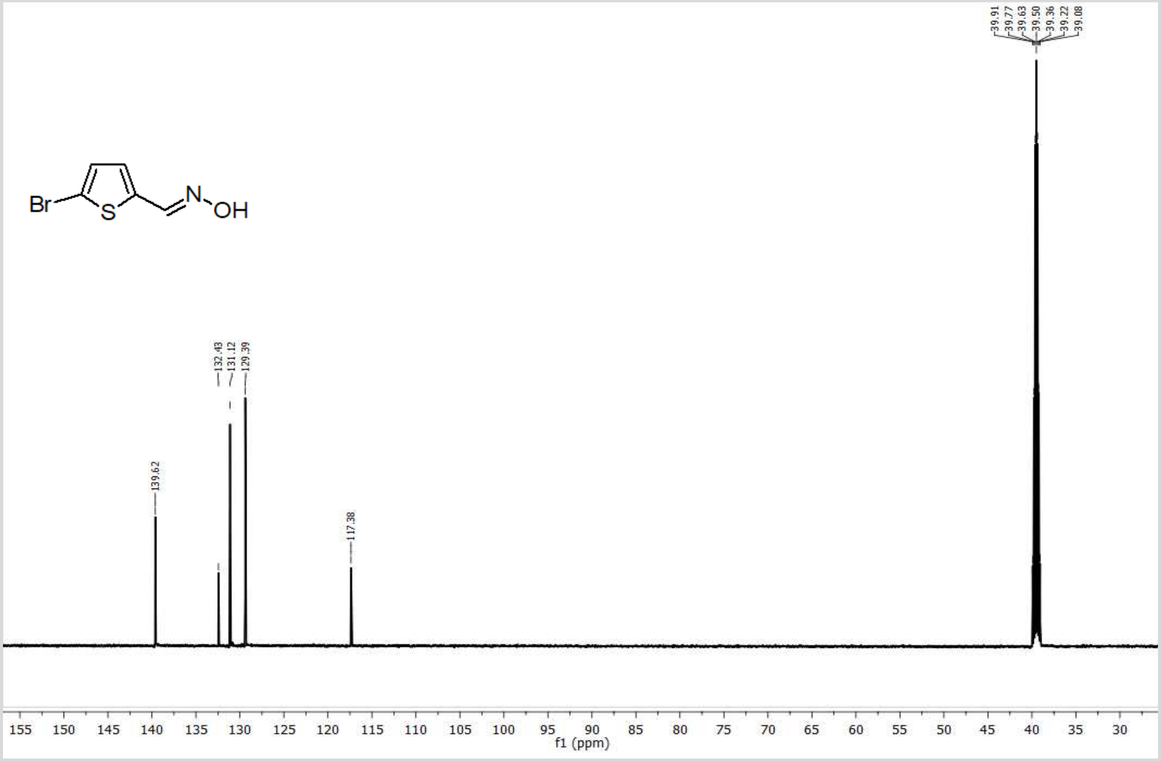

Figure S48 – ^{13}C NMR spectrum (150 MHz, DMSO-d_6) of the compound 15.

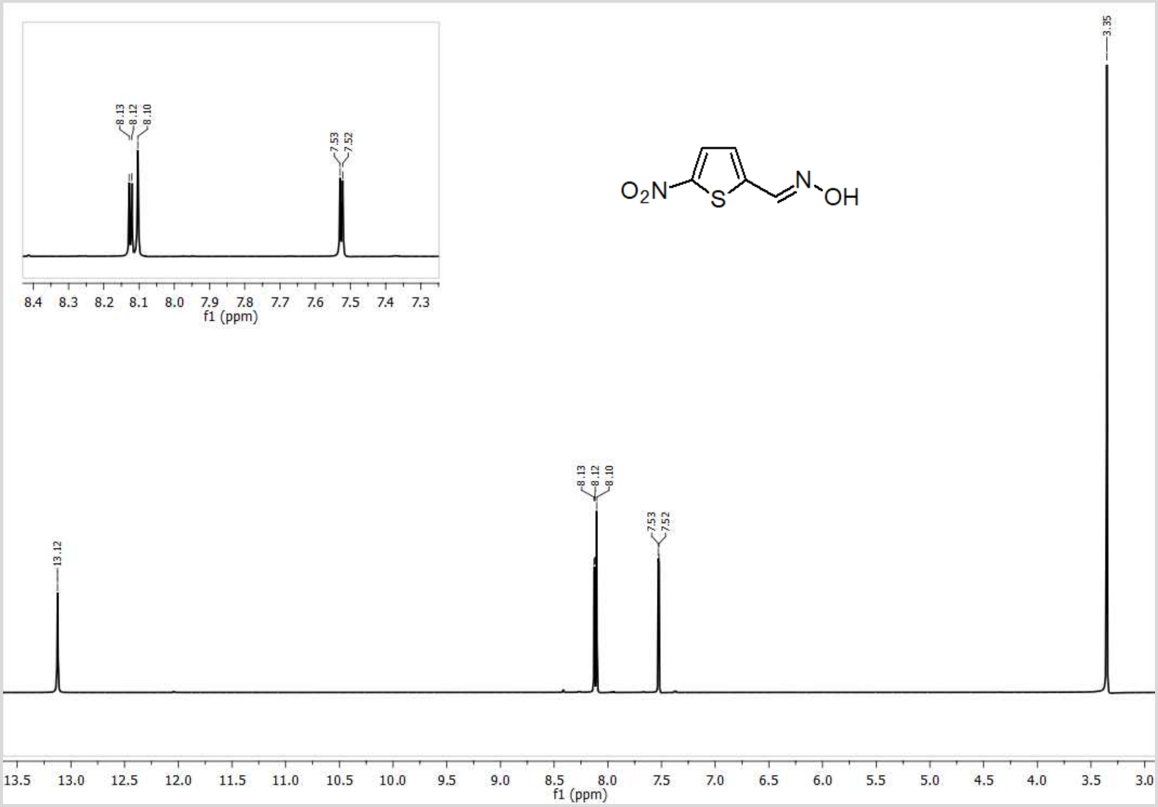

Figure S49 – ^{1}H NMR spectrum (600 MHz, DMSO-d_6) of the compound 16.

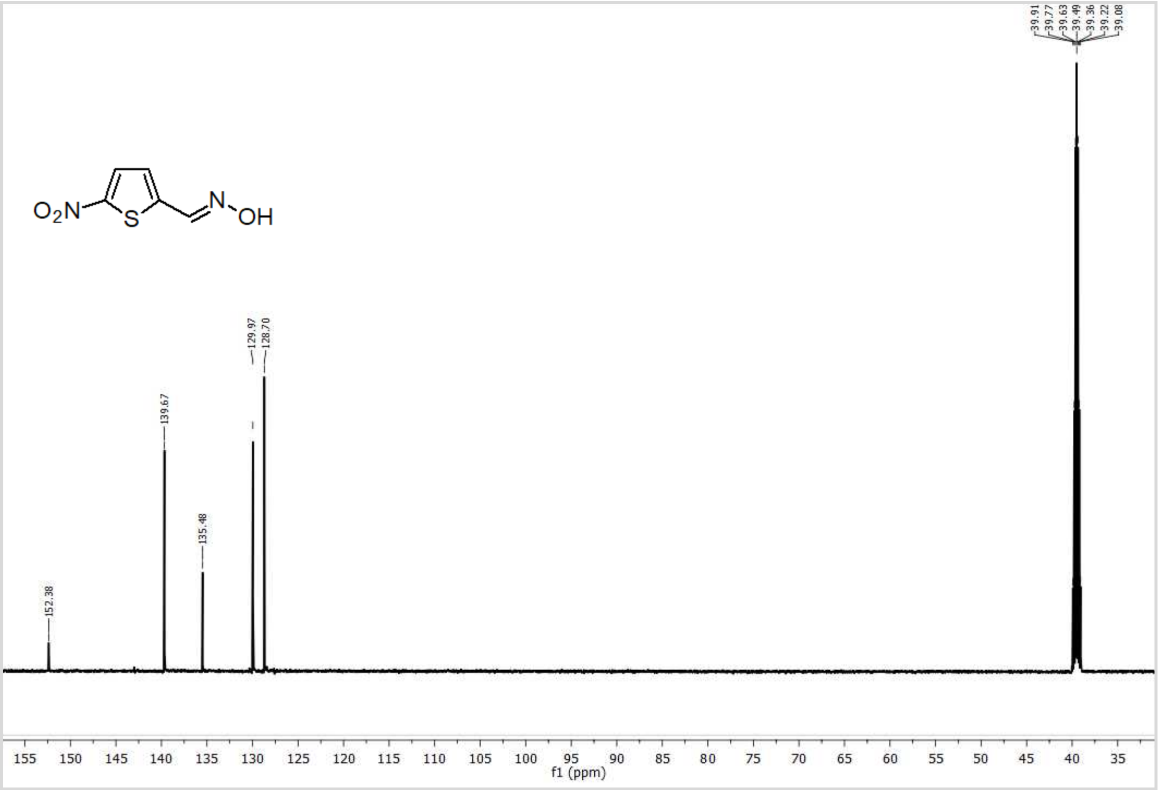

Figure S50 – ^{13}C NMR spectrum (150 MHz, DMSO-d_6) of the compound 16.

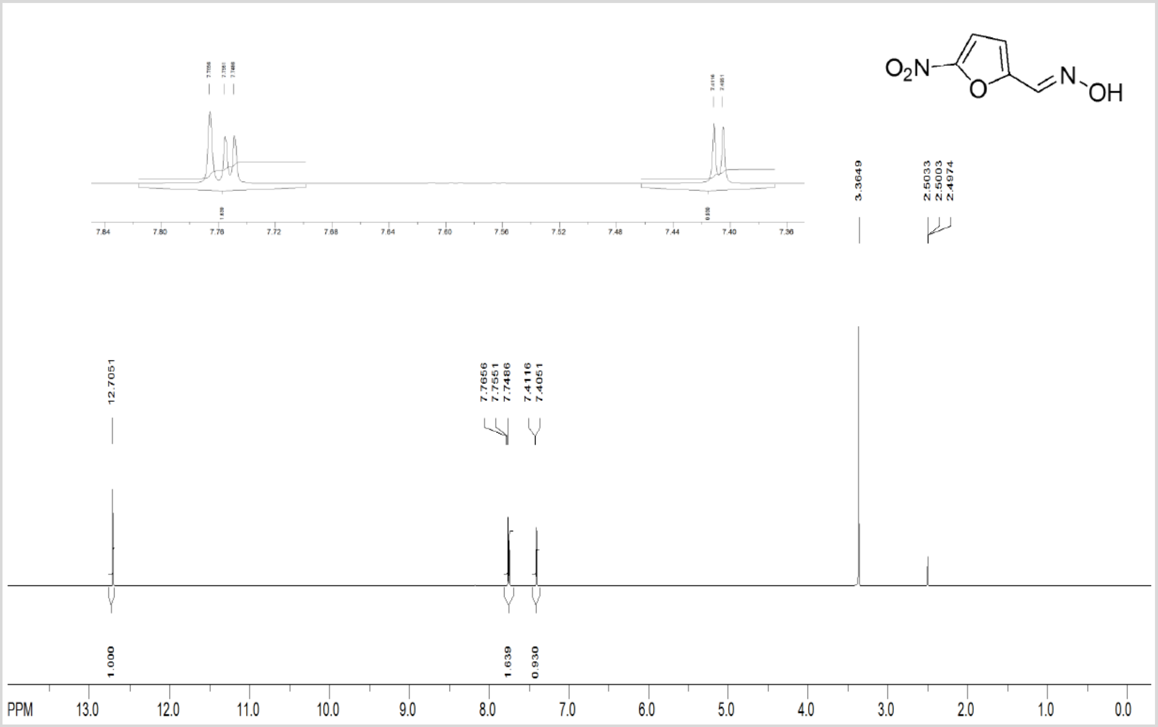

Figure S51 – ^{1}H NMR spectrum (600 MHz, DMSO-d_6) of the compound 17.

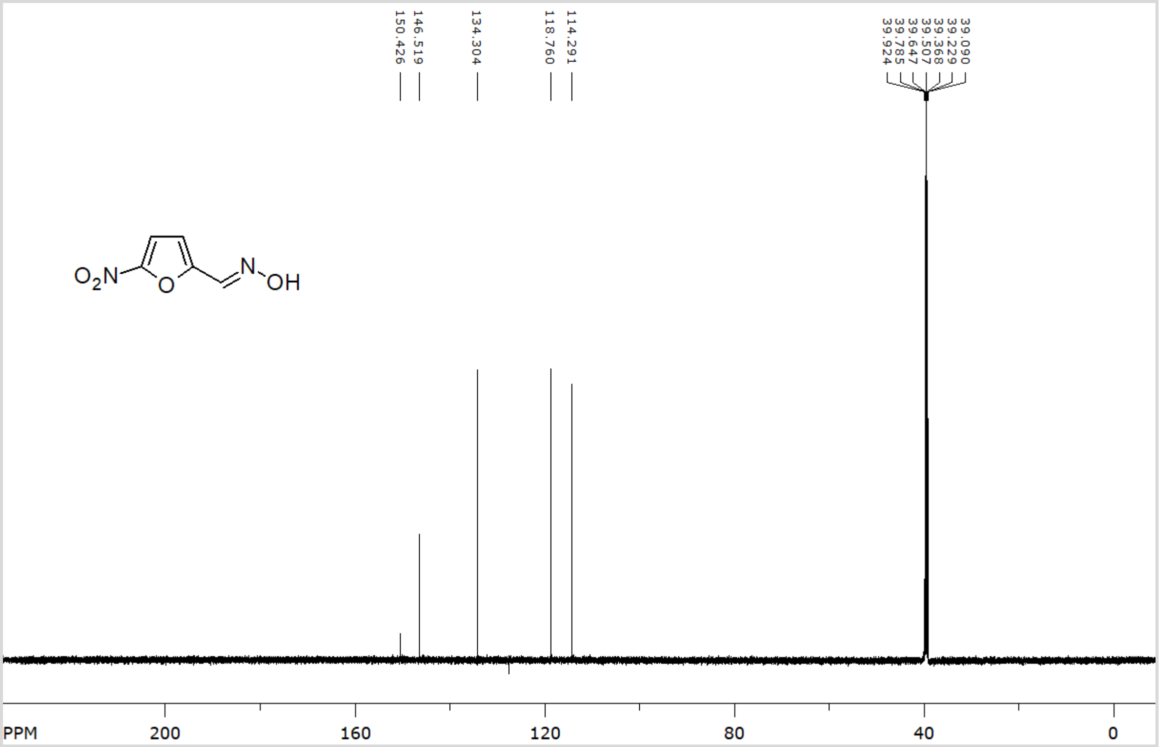

Figure S52 – ^{13}C NMR spectrum (150 MHz, DMSO-d_6) of the compound 17.

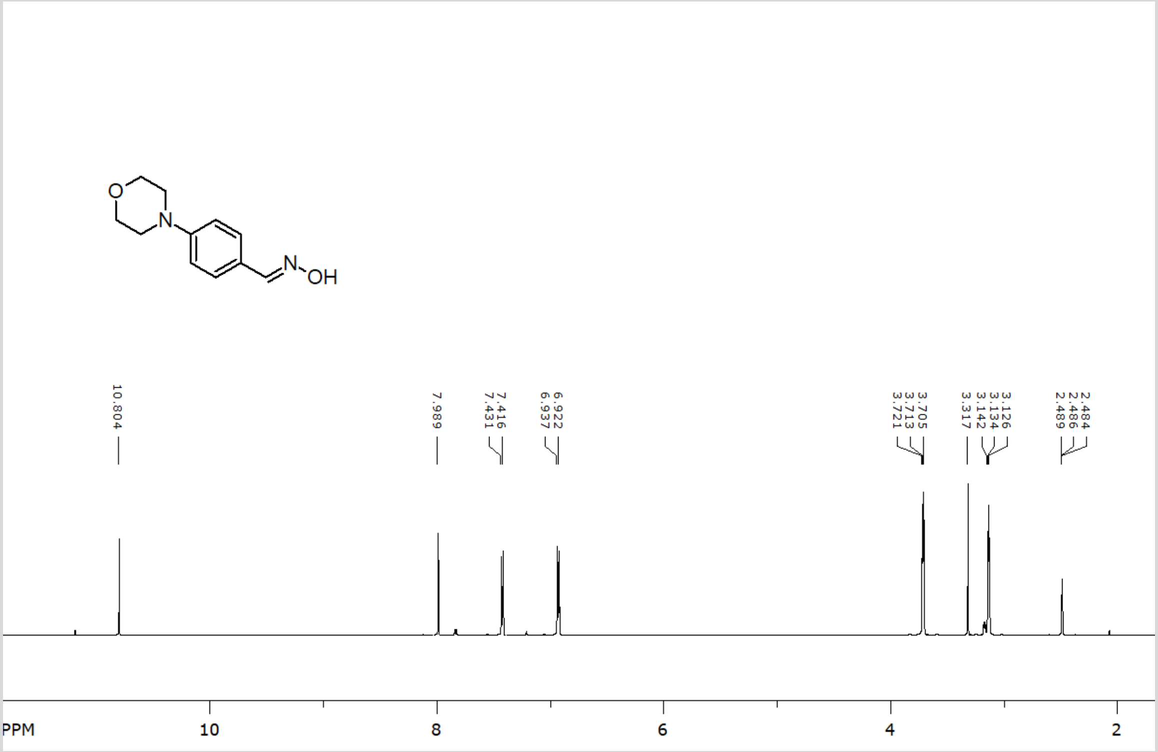

Figure S53 – ^{1}H NMR spectrum (600 MHz, DMSO-d_6) of the compound 18.

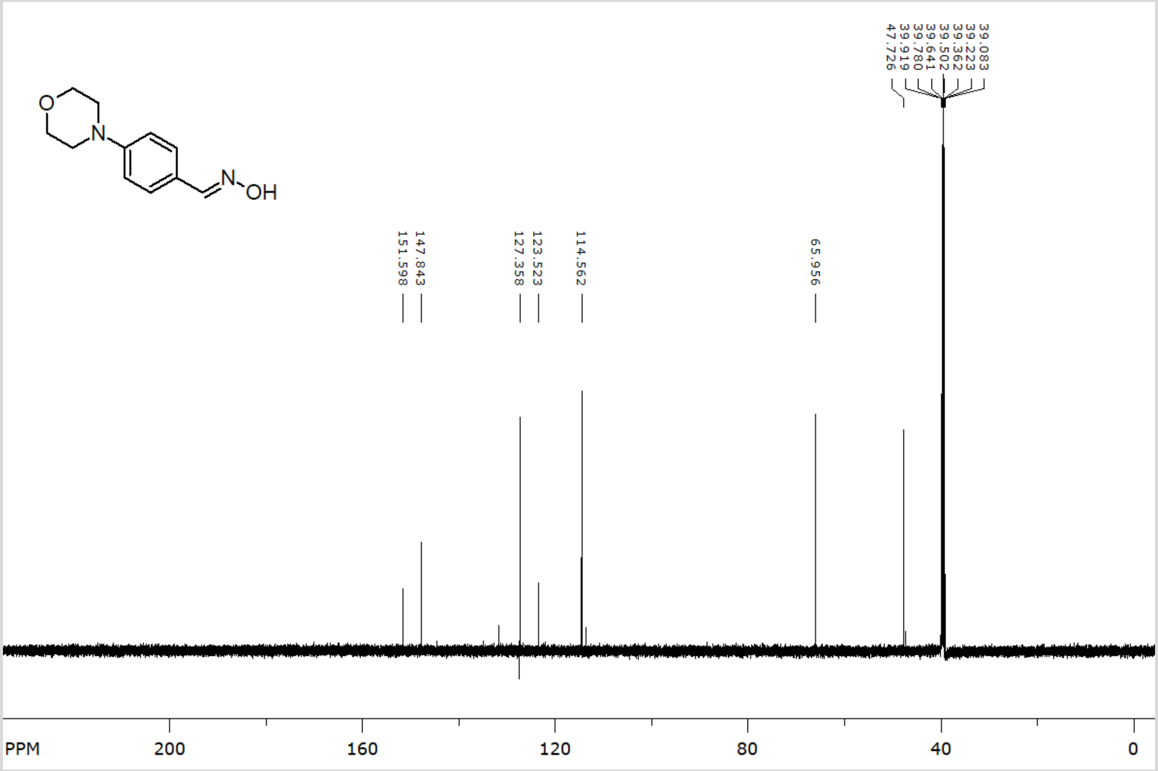

Figure S54 – ^{13}C NMR spectrum (150 MHz, DMSO-d_6) of the compound 18.

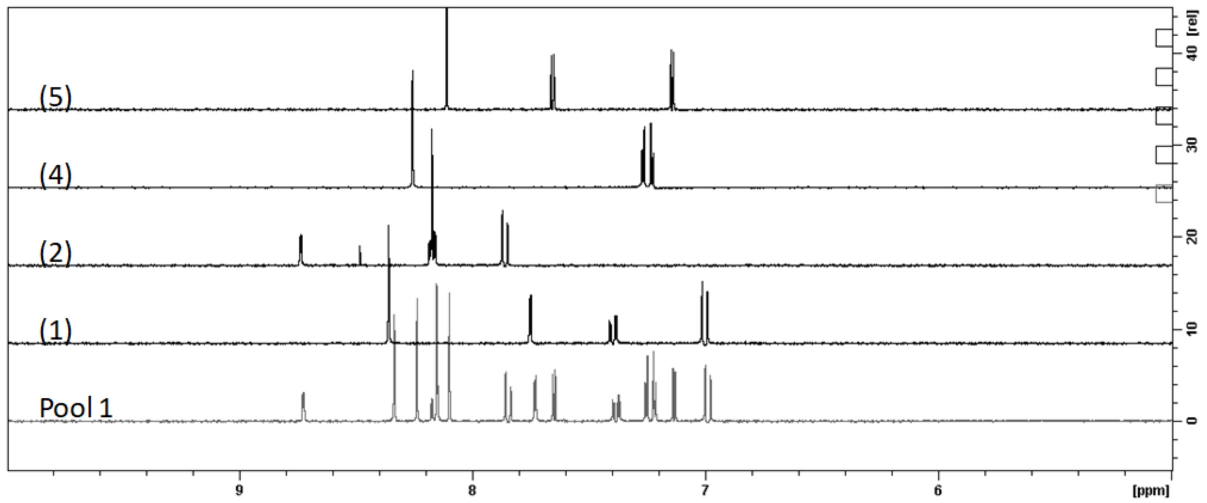

Figure S55 – ¹H spectrum for pool 1 (compounds 1, 2, 4 and 5) and the corresponding ¹H spectra DENVC in PBS buffer H_2O/D_2O (90%/10%).

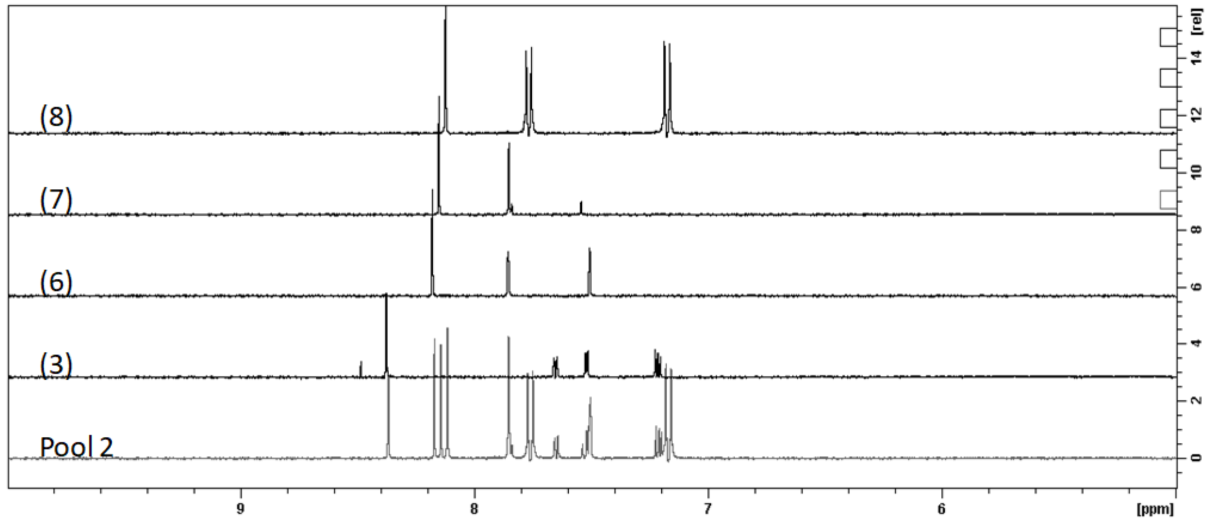

Figure S56 – ¹H spectrum for pool 2 (compounds 3, 6, 7 and 8) and the corresponding ¹H spectra in PBS buffer H_2O/D_2O (90%/10%).

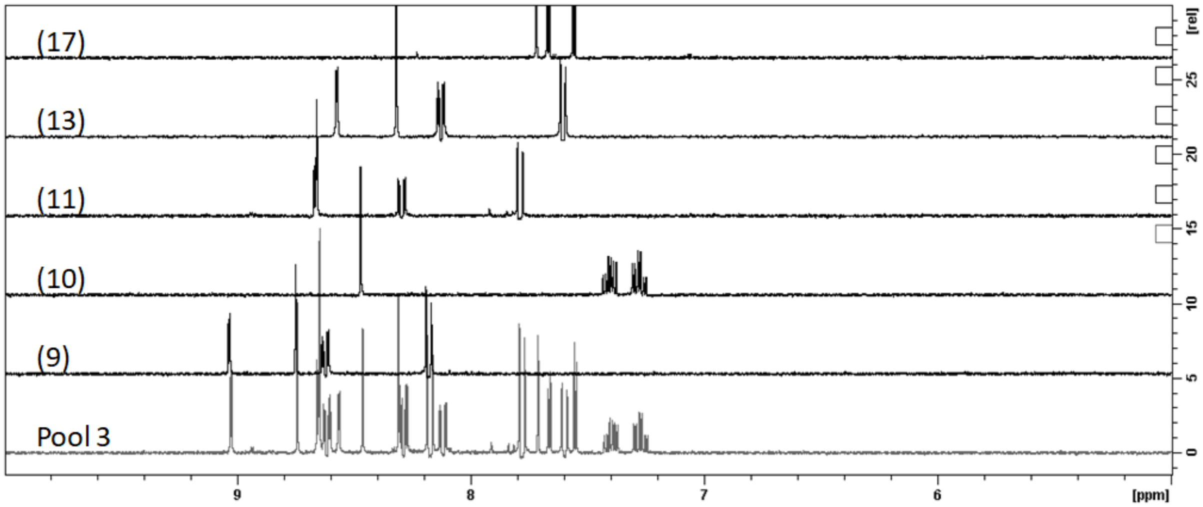

Figure S57 – ^{1}H spectrum for pool 3 (compounds 9, 10, 11, 13 and 17) and the corresponding ^{1}H spectra in PBS buffer H_2O/D_2O (90%/10%).

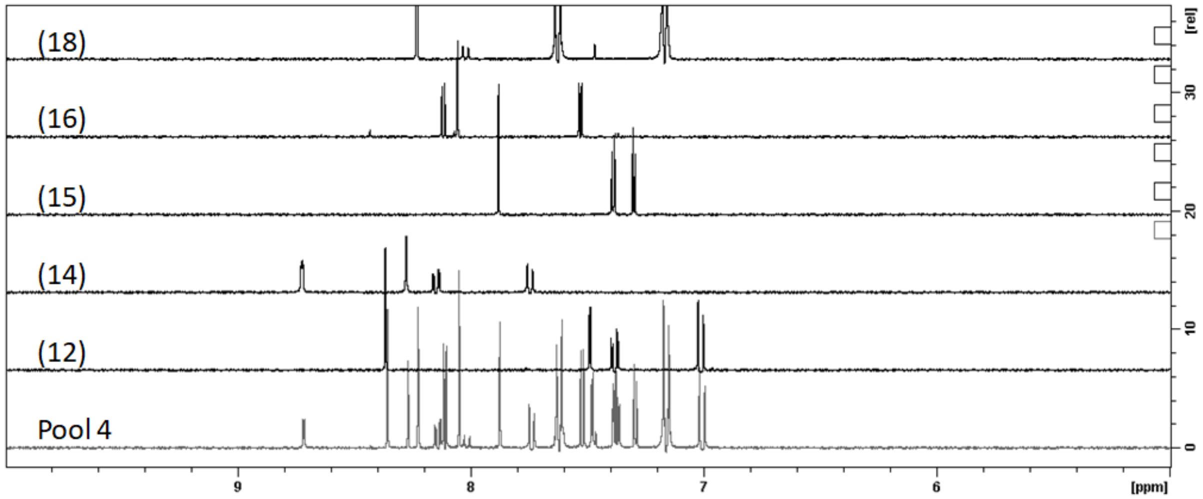

Figure S58 – ^{1}H spectrum for pool 4 (compounds 12, 14, 15, 16 and 18) and the corresponding ^{1}H spectra in PBS buffer H_2O/D_2O (90%/10%).

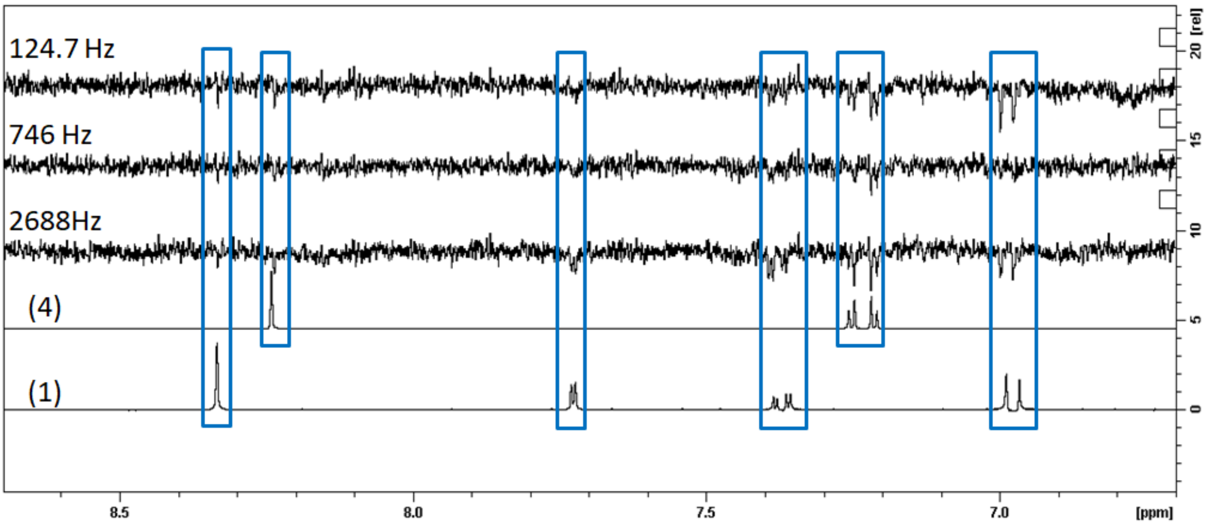

Figure S59 – STD spectra from pool 1 (compounds 1, 2, 4, 5) at different irradiation frequencies (124.7 Hz, 746 Hz and 2688 Hz), and ^{1}H spectra of compounds 1 and 4. 1 mM of each compound and 10 μM of DENVC in PBS buffer H_2O/D_2O (90%/10%) were used. The STD signals are highlighted by the blue boxes.

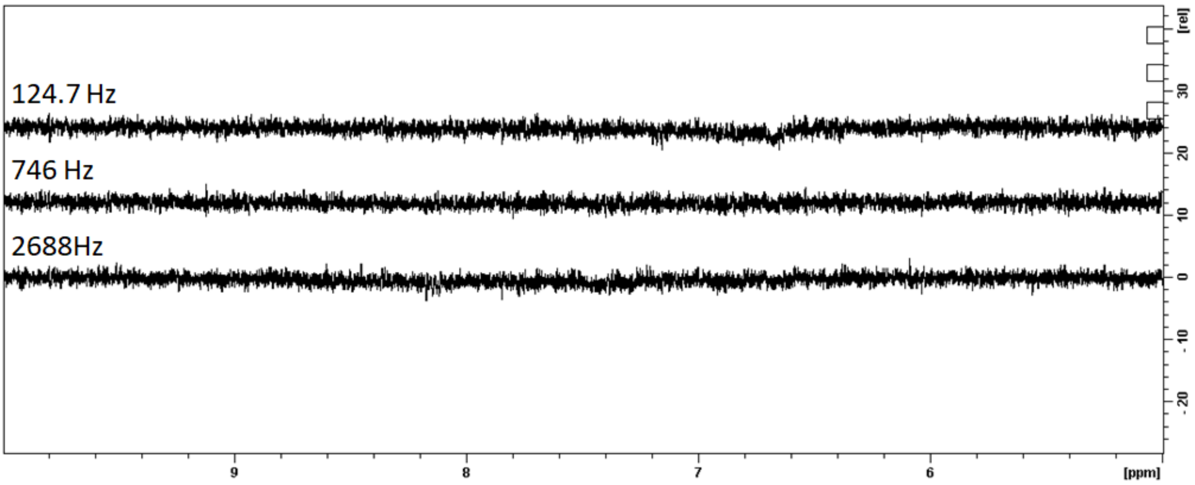

Figure S60 – STD spectra from pool 2 (compounds 3, 6, 7, 8) at different irradiation frequencies (124.7 Hz, 746 Hz and 2688 Hz). 1 mM of each compound and 10 μM of DENVC in PBS buffer H_2O/D_2O (90%/10%) were used. N

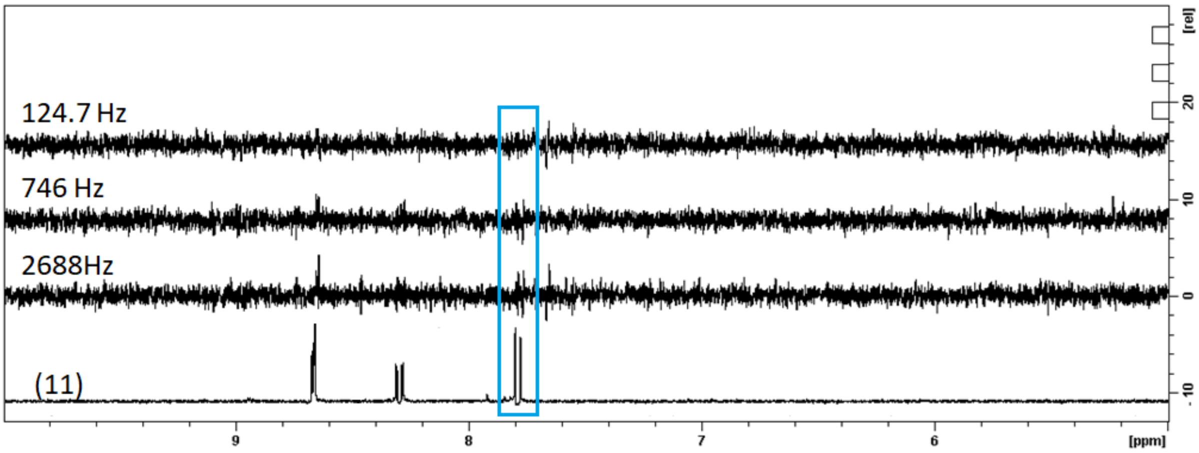

Figure S61 – STD spectra from pool 3 (compounds 9, 10, 11, 13, 17) at different irradiation frequencies (124.7 Hz, 746 Hz and 2688 Hz). 1 mM of each compound and 10 μM of DENVC in PBS buffer H_2O/D_2O (90%/10%) were used. The STD signals are highlighted by the blue boxes. Note that the STD signal for compound 11 is near the noise. For this reason, it was not selected for further studies.

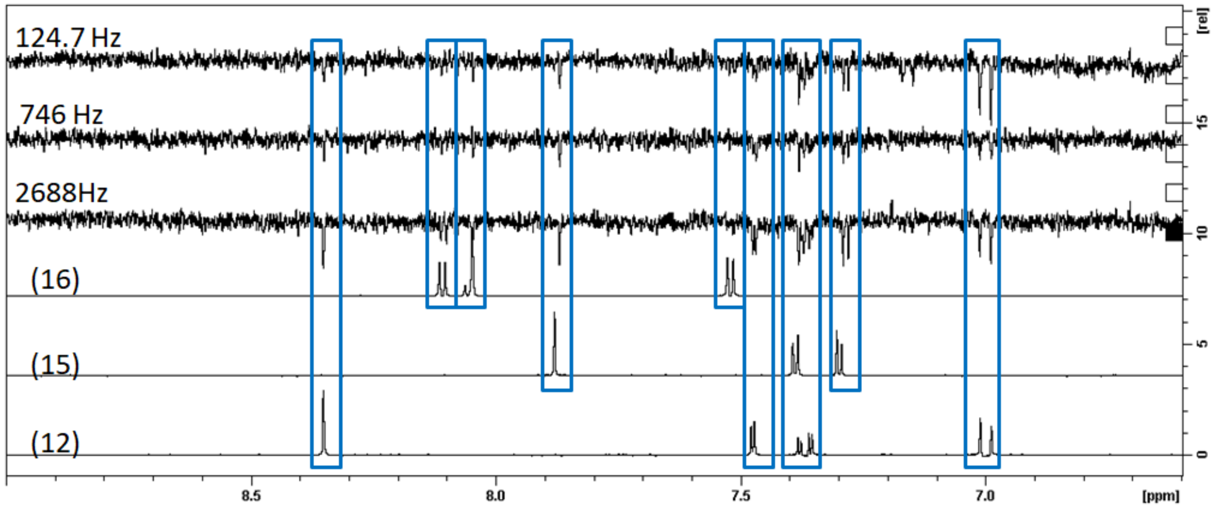

Figure S62 – STD spectra from pool 4 (compounds 12, 14, 15, 16, 18) at different irradiation frequencies (124.7 Hz, 746 Hz and 2688 Hz) and ^{1}H spectra of compounds 12, 15 and16. 1 mM of each compound and 10 μM of DENVC in PBS buffer H_2O/D_2O (90%/10%) were used. The STD signals are highlighted by the blue boxes.

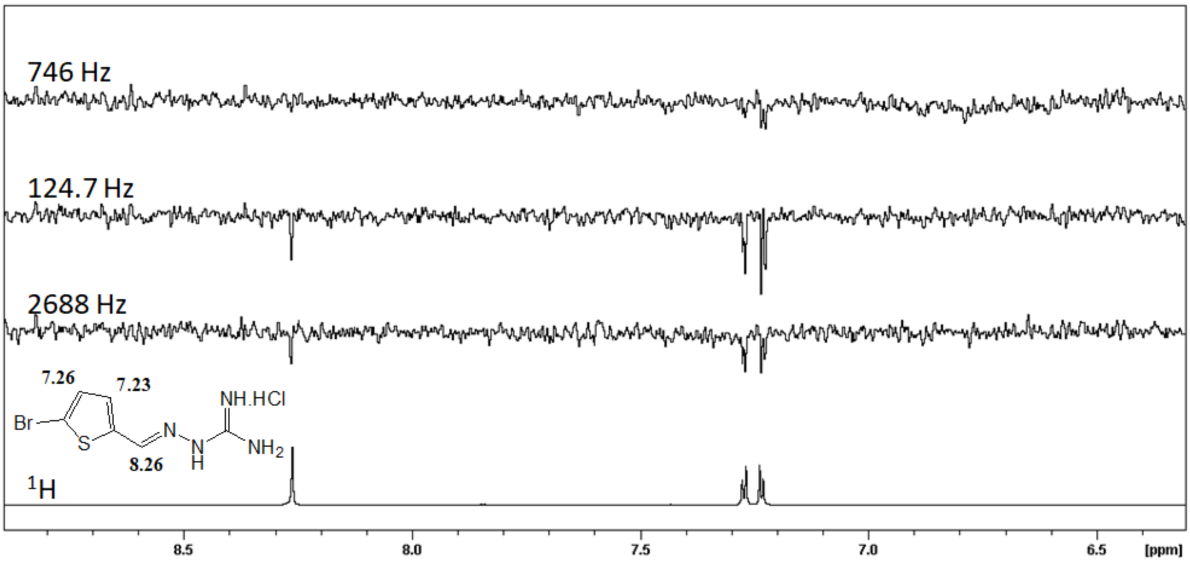

Figure S63 – STD spectrum of 1 mM of compound 4 and 10 µM of DENVC in PBS buffer H_2O/D_2O (90%/10%) at different frequencies of irradiation. At the bottom, the ^{1}H-NMR spectrum of the compound.

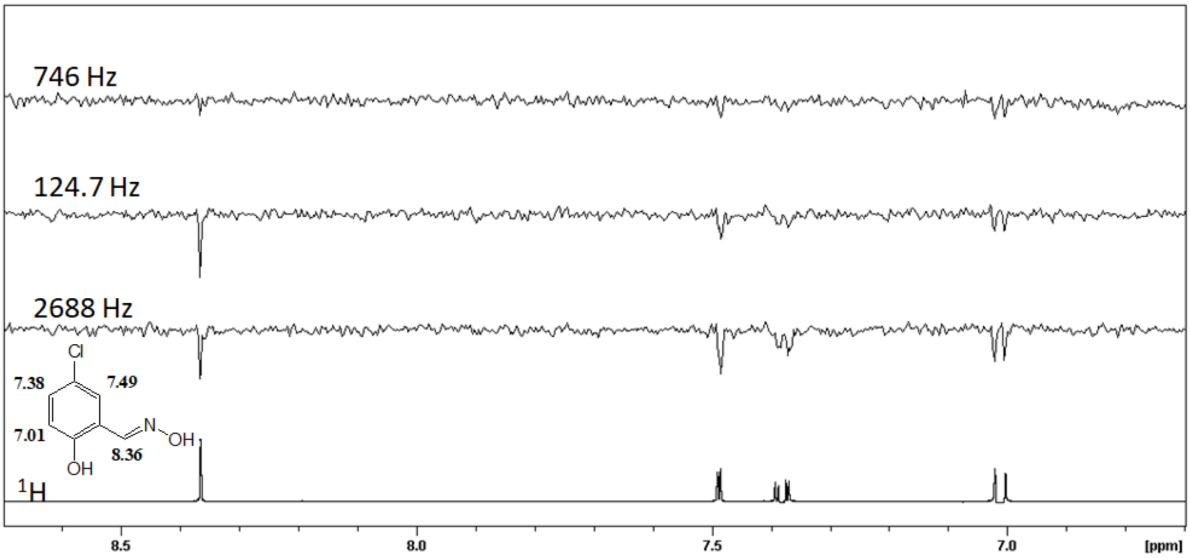

Figure S64 – STD spectrum of 1 mM of compound 12 and 10 µM of DENVC in PBS buffer H_2O/D_2O (90%/10%) at different frequencies of irradiation. At the bottom, the ^{1}H-NMR spectrum of the compound.

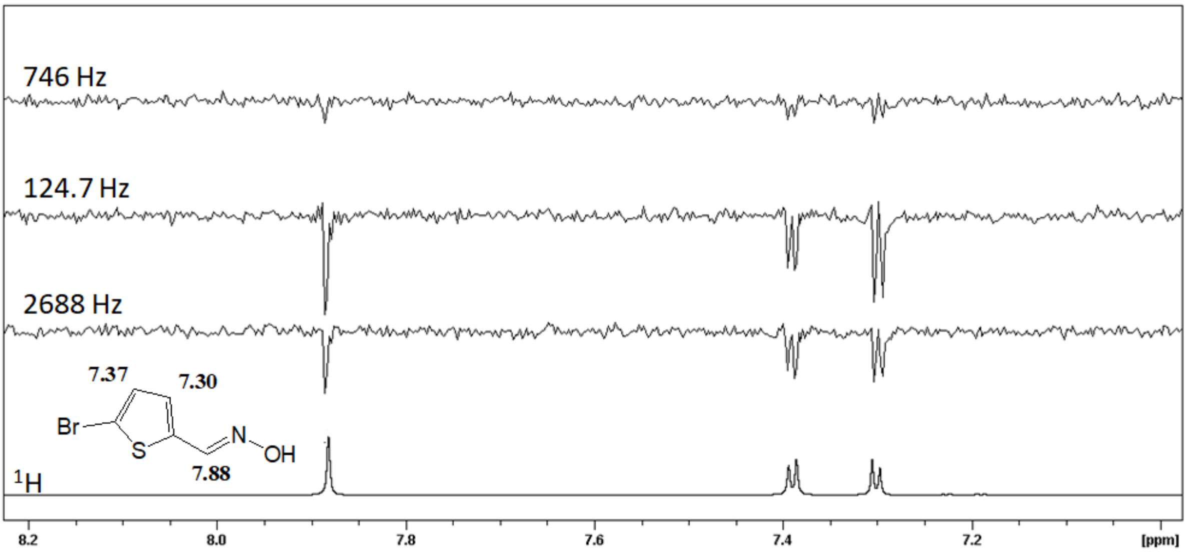

Figure S65 – STD spectrum of 1 mM of compound 15 and 10 µM of DENVC in PBS buffer H_2O/D_2O (90%/10%) at different frequencies of irradiation. At the bottom, the ^1H-NMR spectrum of the compound.

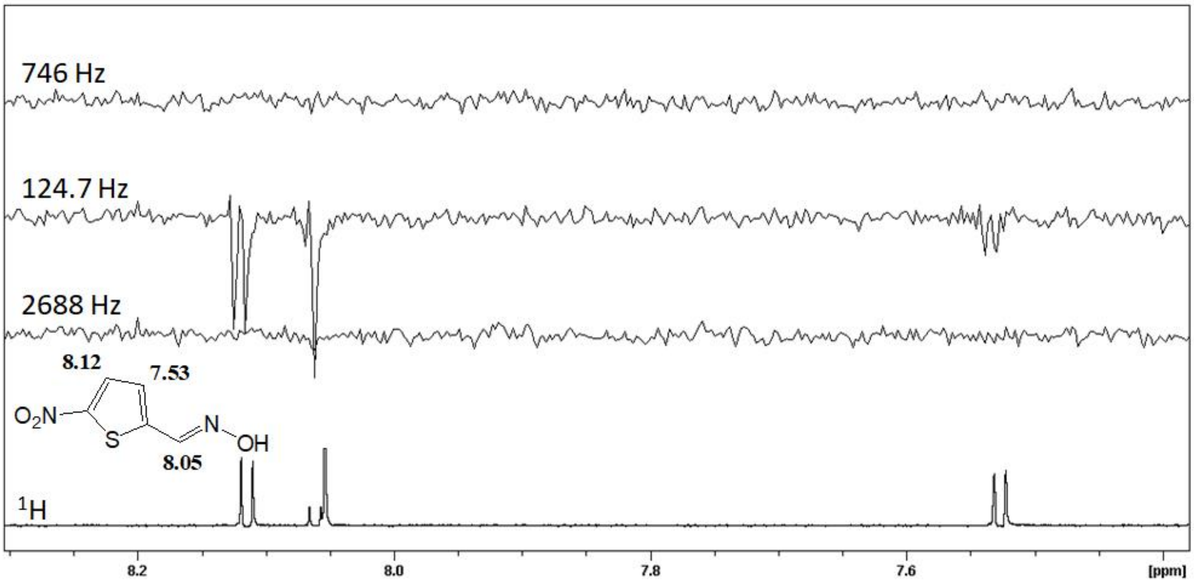

Figure S66 – STD spectrum of 1 mM of compound 16 and 10 µM of DENVC in PBS buffer H_2O/D_2O (90%/10%) at different frequencies of irradiation. At the bottom, the ^1H-NMR spectrum of the compound.

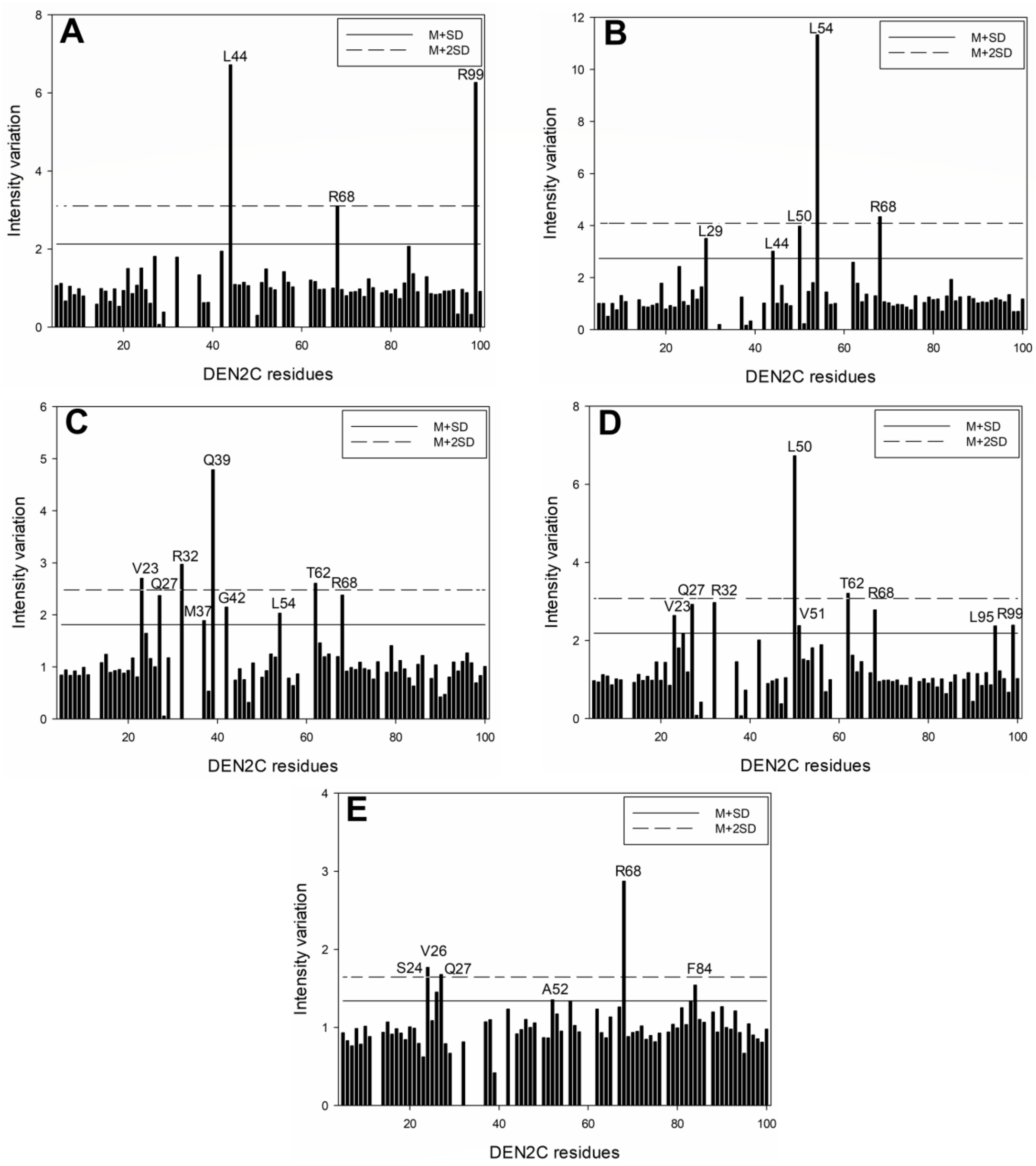

Figure S67 – DENVC (200 μM) changes in intensity mapped by HSQC ^{1}H-^{15}N in the absence and presence of 1.8 mM of (A) compound 1; (B) compound 4; (C) compound 12; (D) compound 15; (E) compound 16.

Table S1 – The residues that most interacted according to the average plus standard deviation (M + SD) and the average plus twice standard deviation (M + 2SD). Changes in chemical shift (CSP) are shown in black, changes in intensity are shown in red and the changes in both are shown in blue.

Compound	Residues (M + SD)	Residues (M + 2SD)
**1**	V23, L29, R32, Q39, G42, L44, L50, L54, R68, W69, I72, L95, R99	Q39, L44, L50, L54, R99
**4**	V23, Q28, L29, L38, Q39, L44, L50, L54, F56, R68, W69, I72	V23, Q39, L54, R68, W69
**12**	E19, V23, Q27, L29, R32, M37, Q39, G42, L44, L50, A52, L54, T62, R68, W69	V23, L29, R32, Q39, L44, T62, W69
**15**	V23, Q27, Q28, L29, R32, L38, L44, L50, V51, T62, R68, W69, L95, R99	Q28, L29, L44, L50, T62, W69
**16**	S24, V26, Q27, Q39, F47, L50, A52, L54, R68, W69, F84, N96, R97, R98, R99, R100	S24, Q27, Q39, L54, R68, R98

Tables S2-S6 – Structural details of the non-covalent interactions determined from PLIP server for the structural models of the DENVC/compound complexes.

Table S2 – **Compound 1.**

Hydrophobic interactions								
Run 1			Run 2			Run 3		
Residue	AA	Distance (Å)	Residue	AA	Distance (Å)	Residue	AA	Distance (Å)
29A	LEU	3.58	43A	PRO	3.66	43B	PRO	3.55
32A	ARG	3.47	46A	LEU	3.62	46B	LEU	3.63
33A	PHE	3.49				46B	LEU	3.72
44A	LEU	3.18						
46A	LEU	3.69						
47A	PHE	3.76						

Hydrogen bonds						
Residue	AA	Distance H-A (Å)	Distance D-A (Å)	Donor Angle (°)	Protein donor?	Sidechain
**Run 1**						
41A	ARG	1.93	2.89	163.63	✓	✓
**Run 2**						
29A	LEU	2.93	3.54	120.60	✗	✗
41A	ARG	3.14	3.86	130.61	✓	✓
44A	LEU	3.57	3.91	103.24	✓	✗
47A	PHE	3.63	4.05	108.31	✓	✗

			Run 3			
29B	LEU	2.61	3.34	130.60	✗	✗
41B	ARG	2.97	3.72	133.73	✓	✓
44B	LEU	3.47	3.86	106.03	✓	✗

π-cation interactions					
**Residue**	**AA**	**Distance (Å)**	**Offset (Å)**	**Protein charged?**	**Ligand Group**
			**Run 2**		
41A	ARG	4.28	1.43	✓	Aromatic
			**Run 3**		
41B	ARG	4.38	1.73	✓	Aromatic

Table S3 – Compound 4.

Hydrophobic interactions								
**Run 1**			**Run 2**			**Run 3**		
**Residue**	**AA**	**Distance (Å)**	**Residue**	**AA**	**Distance (Å)**	**Residue**	**AA**	**Distance (Å)**
43B	PRO	3.50	44A	LEU	3.37	43A	PRO	3.58
46B	LEU	3.89	46A	LEU	3.71			
			47A	PHE	3.58			

Hydrogen bonds						
**Residue**	**AA**	**Distance H-A (Å)**	**Distance D-A (Å)**	**Donor Angle (°)**	**Protein donor?**	**Sidechain**
			**Run 1**			
29B	LEU	2.41	3.25	142.85	✗	✗
			**Run 2**			
41A	ARG	2.10	3.04	158.62	✓	✓
			**Run 3**			
29A	LEU	2.55	3.28	131.12	✗	✗
47A	PHE	3.61	4.08	112.14	✓	✗

π -cation interactions					
**Residue**	**AA**	**Distance (Å)**	**Offset (Å)**	**Protein charged?**	**Ligand Group**
			**Run 1**		
41B	ARG	4.30	0.73	✓	Aromatic
			**Run 3**		
41A	ARG	4.21	0.66	✓	Aromatic

Halogen Bonds				
Residue	AA	Distance (Å)	Donor Angle (°)	Acceptor Angle (°)
**Run 2**				
68A	ARG	3.83	175.07	107.67

Table S4 – Compound 12.

Hydrophobic interactions								
Run 1			Run 2			Run 3		
Residue	AA	Distance (Å)	Residue	AA	Distance (Å)	Residue	AA	Distance (Å)
29A	LEU	3.47	29A	LEU	3.59	29A	LEU	3.48
32A	ARG	3.49	32A	ARG	3.86	32A	ARG	3.49
33A	PHE	3.61	33A	PHE	3.57	33A	PHE	3.65
44A	LEU	3.16	44A	LEU	3.22	44A	LEU	3.17
46A	LEU	3.81	46A	LEU	3.77	46A	LEU	3.80
47A	PHE	3.70	47A	PHE	3.33	47A	PHE	3.71

Hydrogen bonds						
Residue	AA	Distance H-A (Å)	Distance D-A (Å)	Donor Angle (°)	Protein donor?	Sidechain
**Run 1**						
41A	ARG	1.77	2.71	159.93	✓	✓
41A	ARG	2.27	3.12	139.72	✗	✓
**Run 2**						
28A	GLN	3.22	3.90	127.41	✓	✓
28A	GLN	2.31	2.81	112.79	✗	✓
32A	ARG	3.32	4.06	133.03	✓	✓
41A	ARG	2.63	3.38	132.87	✓	✓
**Run 3**						
41A	ARG	1.76	2.71	159.46	✓	✓

Table S5 – Compound 15.

Hydrophobic interactions								
Run 1			Run 2			Run 3		
Residue	AA	Distance (Å)	Residue	AA	Distance (Å)	Residue	AA	Distance (Å)
29B	LEU	3.25	29B	LEU	3.30	29B	LEU	3.25
32B	ARG	3.71	32B	ARG	3.72	32B	ARG	3.63
46B	LEU	3.49	46B	LEU	3.39	46B	LEU	3.56
47B	PHE	3.86	47B	PHE	3.86	47B	PHE	3.96

Hydrogen bonds						
Residue	AA	Distance H-A (Å)	Distance D-A (Å)	Donor Angle (°)	Protein donor?	Sidechain
**Run 1**						
41B	ARG	2.72	3.05	101.68	✗	✓
41B	ARG	2.21	3.08	147.10	✓	✓
44B	LEU	3.01	3.90	151.01	✓	✗
**Run 2**						
41B	ARG	2.22	3.11	149.58	✓	✓
44B	LEU	3.12	4.02	152.77	✓	✗
**Run 3**						
41B	ARG	2.70	3.05	102.53	✗	✓
41B	ARG	2.19	3.06	146.62	✓	✓
44B	LEU	2.99	3.88	150.93	✓	✗

Halogen Bonds				
Residue	AA	Distance (Å)	Donor Angle (°)	Acceptor Angle (°)
**Run 1**				
68B	ARG	3.83	169.13	108.09
**Run 2**				
68B	ARG	3.94	169.86	107.81
**Run 3**				
68B	ARG	3.83	170.08	107.75

Table S6 – Compound 16.

Hydrophobic interactions								
Run 1			Run 2			Run 3		
Residue	AA	Distance (Å)	Residue	AA	Distance (Å)	Residue	AA	Distance (Å)
29A	LEU	3.25	29A	LEU	3.33	29A	LEU	3.28
32A	ARG	3.74	32A	ARG	3.64	32A	ARG	3.69
46A	LEU	3.71	33A	PHE	3.85	33A	PHE	3.93
47A	PHE	3.69	46A	LEU	3.87	46A	LEU	3.81
			47A	PHE	3.85	47A	PHE	3.77

Hydrogen bonds						
Residue	AA	Distance H-A (Å)	Distance D-A (Å)	Donor Angle (°)	Protein donor?	Sidechain
**Run 1**						
41A	ARG	2.25	3.12	147.17	✓	✓
44A	LEU	3.14	4.03	150.20	✓	✗

68A	ARG	3.44	4.10	127.05	✘	✔
			**Run 2**			
41A	ARG	2.19	3.06	146.56	✔	✔
41A	ARG	2.76	3.12	104.09	✘	✔
44A	LEU	3.11	3.99	150.78	✔	✘
			**Run 3**			
41A	ARG	2.21	3.08	146.68	✔	✔
41A	ARG	2.75	3.10	103.20	✘	✔
44A	LEU	3.12	4.01	150.28	✔	✘

References

[1] Prasad RN, McKay AF. Acylation of guanidines and guanylhydrazones. Can J Chem. 1967;45:2247–2252.

[2] Amidi S, Esfahanizadeh M, Tabib K, et al. Rational Design and Synthesis of 1-(Arylideneamino)-4-aryl-1 H -imidazole-2-amine Derivatives as Antiplatelet Agents. ChemMedChem. 2017;12:962–971.

[3] Bonomi P, Servant A, Resmini M. Modulation of imprinting efficiency in nanogels with catalytic activity in the Kemp elimination. J Mol Recognit. 2012;25:352–360.

[4] Jiménez-Juárez R, Cruz-Chávez W, de Jesús-Ramírez N, et al. Synthesis and Antimycobacterial Activity of 2,5-Disubstituted and 1,2,5-Trisubstituted Benzimidazoles. Front Chem. 2020;8.

[5] Irfan I, Sawangjaroen N, Bhat AR, et al. New dioxazole derivatives: Synthesis and effects on the growth of Entamoeba histolytica and Giardia intestinalis. Eur J Med Chem. 2010;45:1648–1653.

[6] Trefzger OS, Barbosa N V., Scapolatempo RL, et al. Design, synthesis, antileishmanial, and antifungal biological evaluation of novel 3,5-disubstituted isoxazole compounds based on 5-nitrofuran scaffolds. Arch Pharm (Weinheim). 2020;353:1900241.
